# Supplementary material for: Novel mechanism for tubular injury in nephropathic cystinosis
Source: eLife. 2025 Mar 20;13:RP94169. doi: 10.7554/eLife.94169 (PMC11925453; doi:10.7554/eLife.94169)
Supplement: Table 2—source data 1. [file elife-94169-table2-data1.docx]

**Table 2 – source data 1:** List of the 10 v-ATPases showing no significant changes in its expression in cystinosis fibroblasts, CDME-treated fibroblasts and CDME-treated RPTECs compared to their respective controls.

| **Gene Name** | **Fold Change (Fibroblasts)** | **q-value (Fibroblasts)** | **Fold Change (CDME treated Fibroblast)** | **q-value (CDME treated Fibroblast)** | **Fold Change (CDME treated RPTECs)** | **q-value (CDME treated RPTECs)** |
| --- | --- | --- | --- | --- | --- | --- |
| ATP6V0A1 |  |  | - | - | - | - |
| ATP6V1C1 |  |  | - | - | - | - |
| ATP6V1B2 | 0.39 | 0.00 | - | - | - | - |
| ATP6VIE1 |  |  | - | - | - | - |
| ATP6V1H |  |  | - | - | - | - |
| ATP6V0E1 | 1.66 | 0.52 | - | - | 1.58 | 0.45 |
| ATP6V1E2 |  |  | - | - | 1.25 | 2.79 |
| ATP6AP1 | 0.72 | 4.50 | - | - | - | - |
| ATP6V1A |  |  | - | - | - | - |
| ATP6V0D1 |  |  | - | - | - | - |
