# Supplementary figures and images for: Novel mechanism for tubular injury in nephropathic cystinosis
Source: eLife. 2025 Mar 20;13:RP94169. doi: 10.7554/eLife.94169 (PMC11925453; doi:10.7554/eLife.94169)

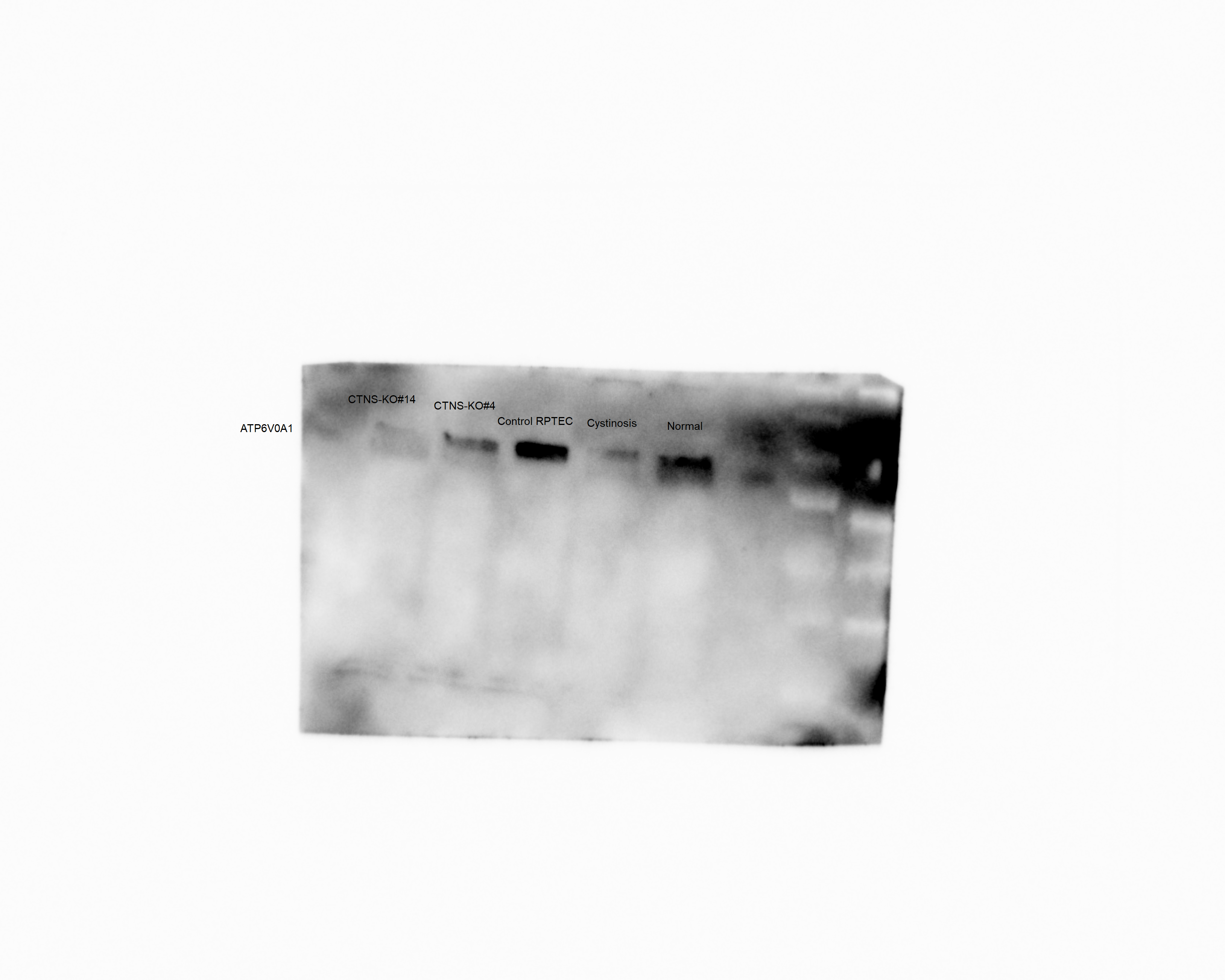

Supplement: Figure 4—source data 2. [file elife-94169-fig4-data2.zip › Figure 4-source data 2/Figure4A/Figure4A_Gel1_ATP6V0A1.tif]

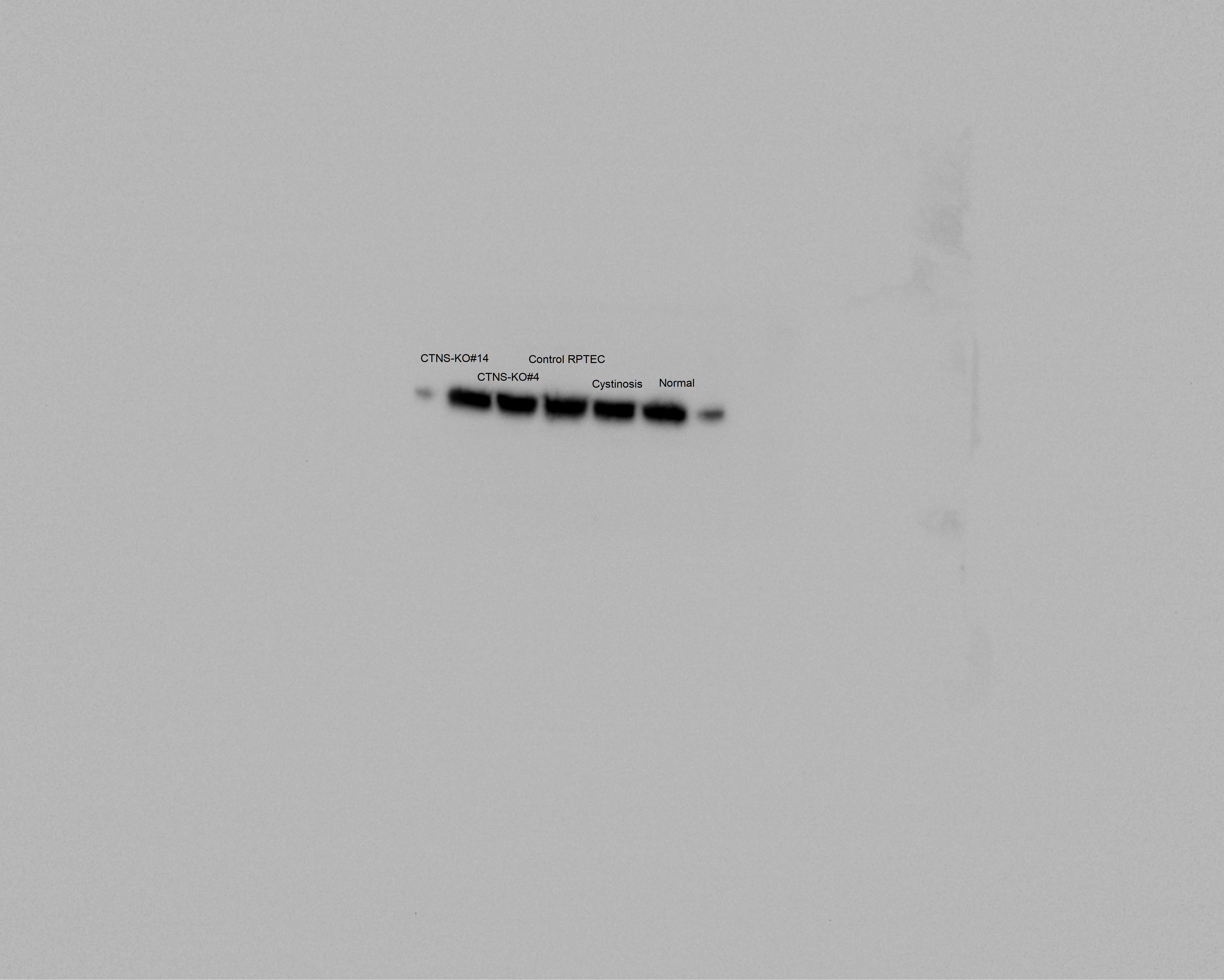

Supplement: Figure 4—source data 2. [file elife-94169-fig4-data2.zip › Figure 4-source data 2/Figure4A/Figure4A_Gel1_BetaTubulin.tif]

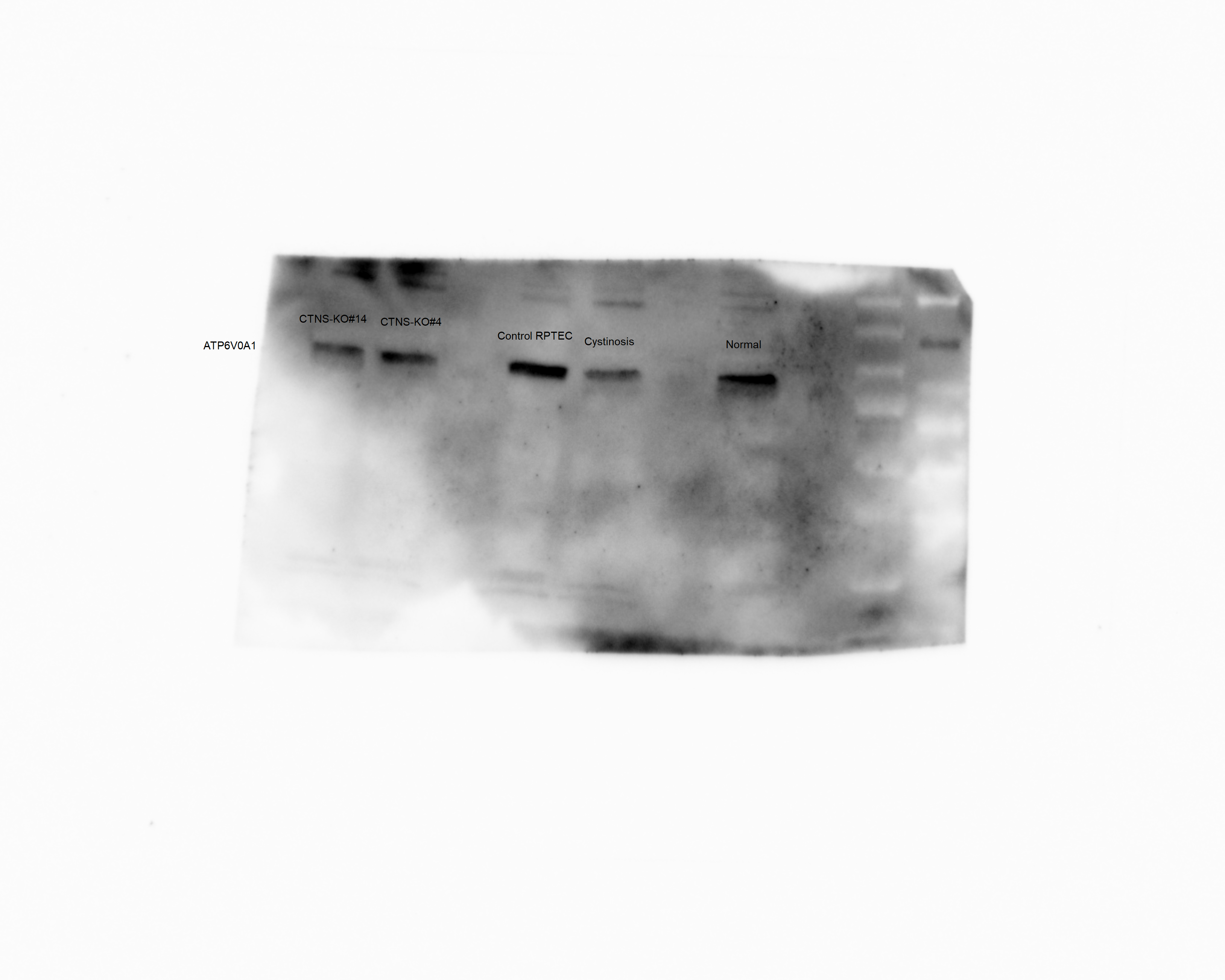

Supplement: Figure 4—source data 2. [file elife-94169-fig4-data2.zip › Figure 4-source data 2/Figure4A/Figure4A_Gel2_ATP6V0A1.tif]

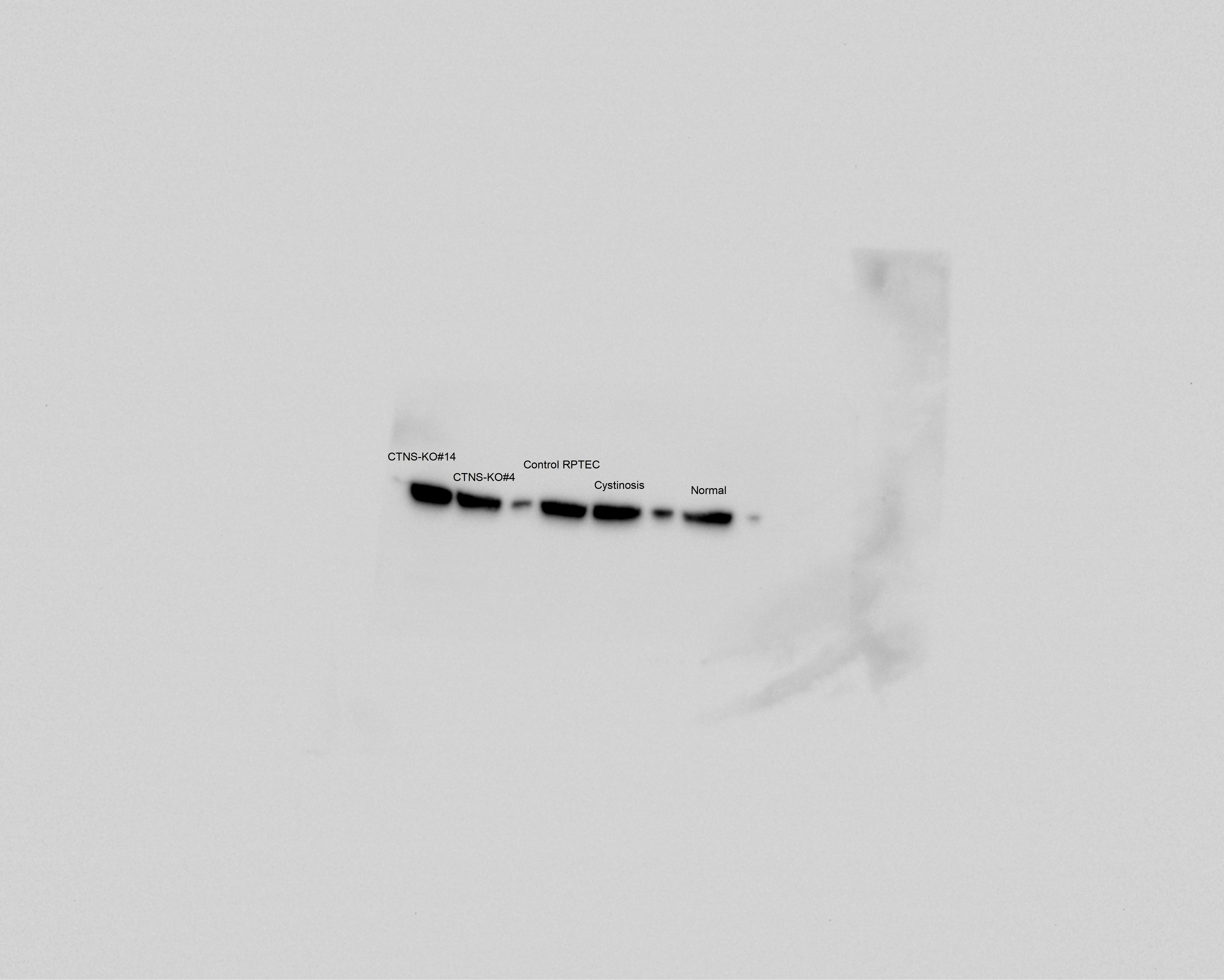

Supplement: Figure 4—source data 2. [file elife-94169-fig4-data2.zip › Figure 4-source data 2/Figure4A/Figure4A_Gel2_BetaTubulin.tif]

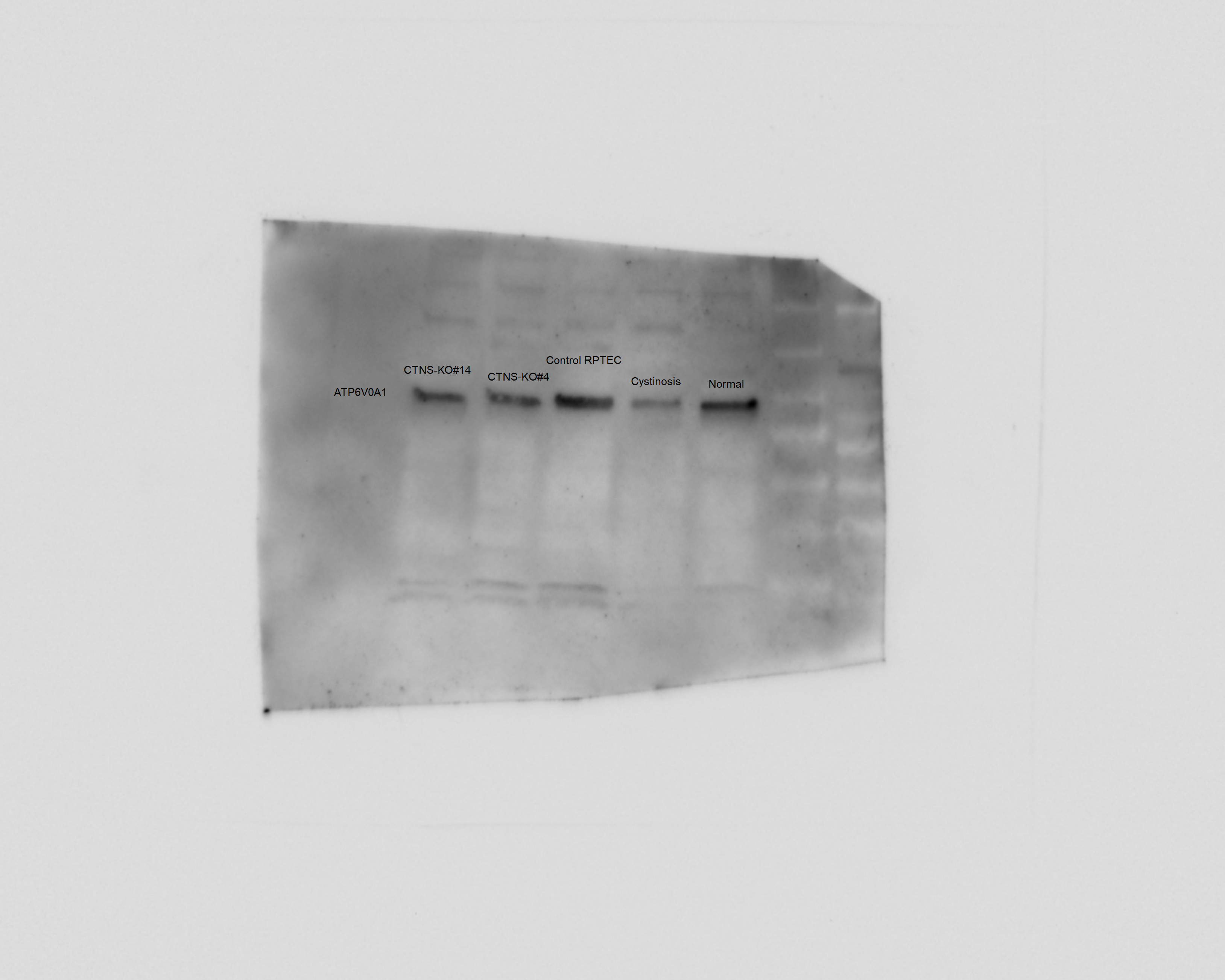

Supplement: Figure 4—source data 2. [file elife-94169-fig4-data2.zip › Figure 4-source data 2/Figure4A/Figure4A_Gel3_ATP6V0A1.tif]

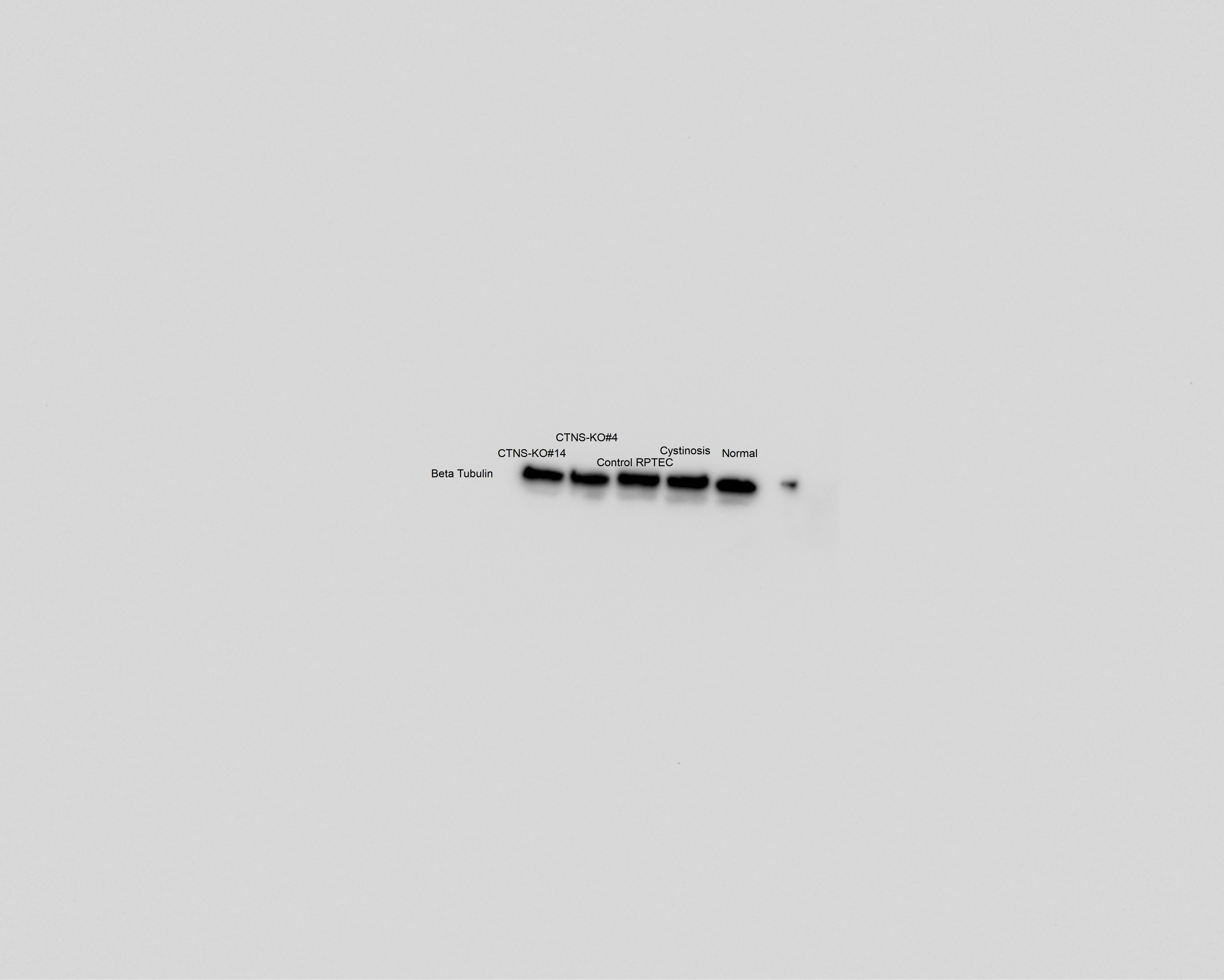

Supplement: Figure 4—source data 2. [file elife-94169-fig4-data2.zip › Figure 4-source data 2/Figure4A/Figure4A_Gel3_BetaTubulin.tif]

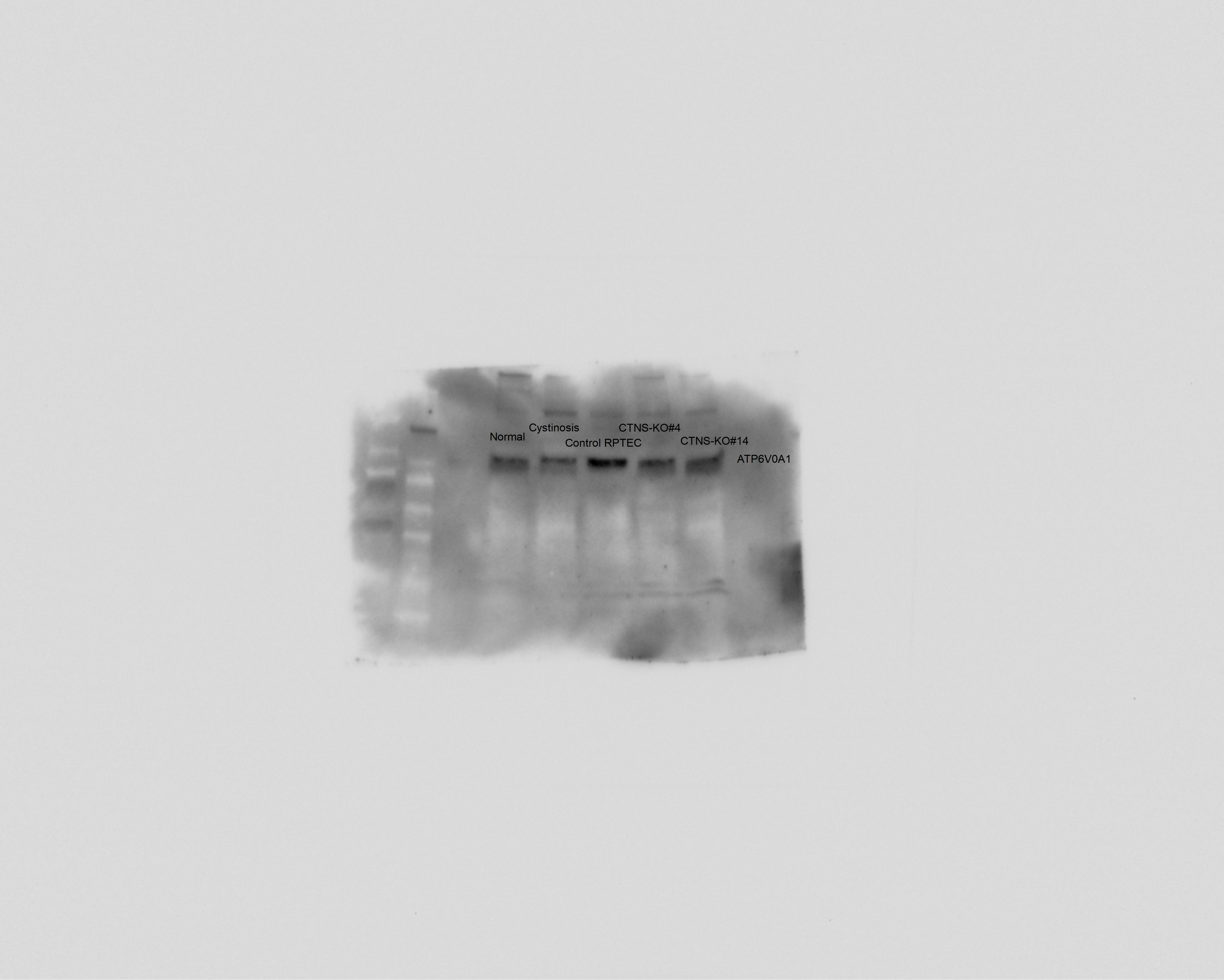

Supplement: Figure 4—source data 2. [file elife-94169-fig4-data2.zip › Figure 4-source data 2/Figure4A/Figure4A_Gel4_ATP6V0A1.tif]

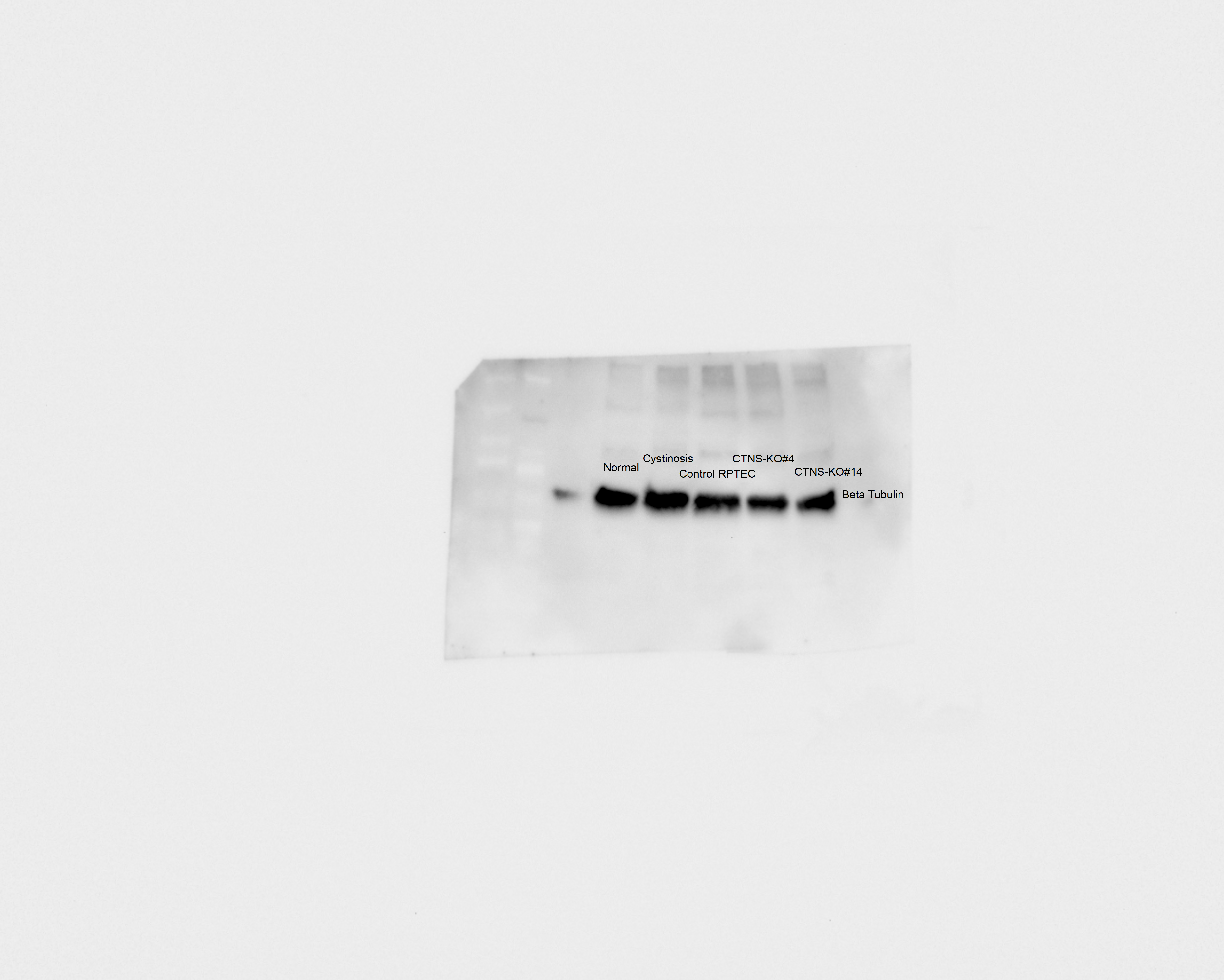

Supplement: Figure 4—source data 2. [file elife-94169-fig4-data2.zip › Figure 4-source data 2/Figure4A/Figure4A_Gel4_BetaTubulin.tif]

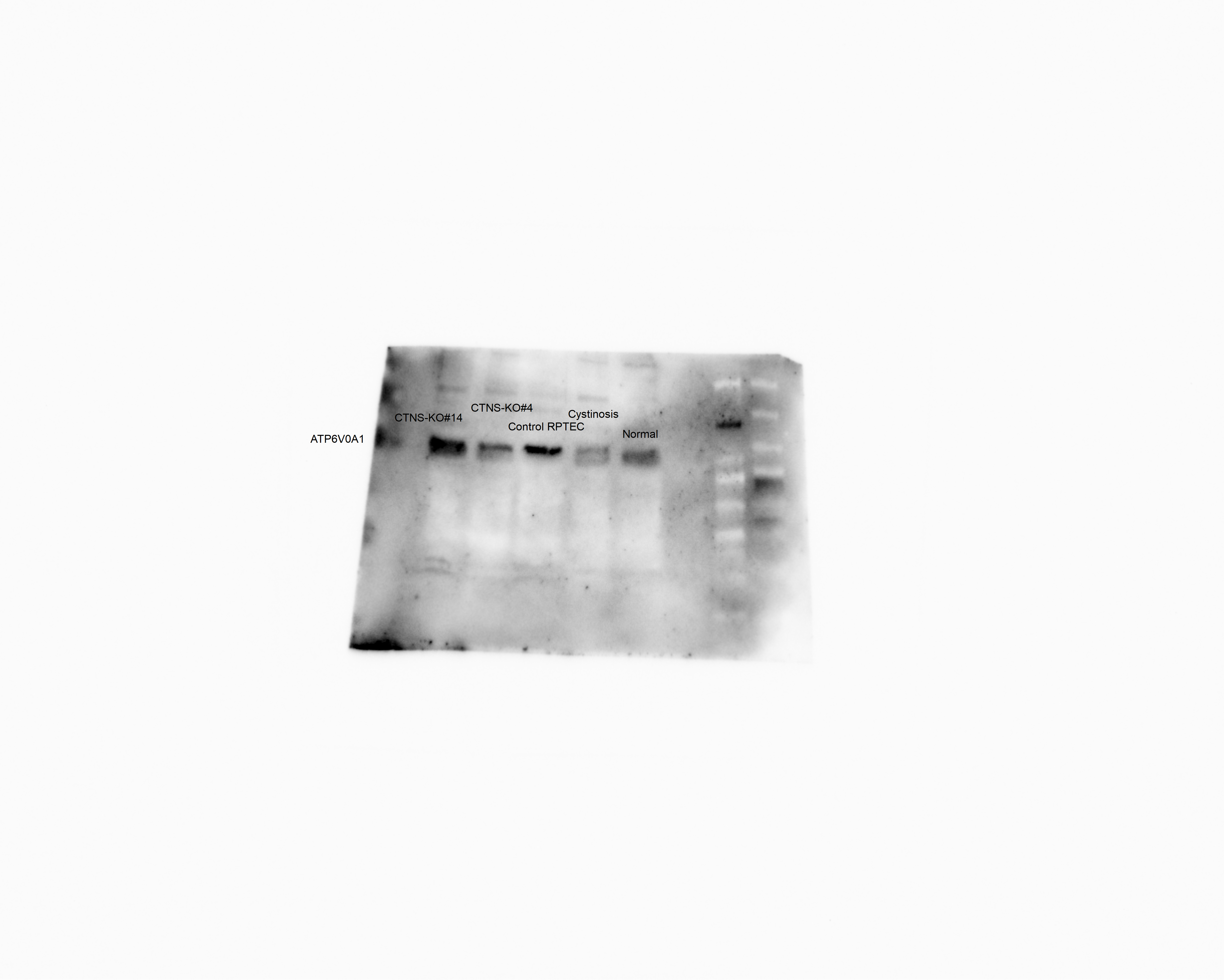

Supplement: Figure 4—source data 2. [file elife-94169-fig4-data2.zip › Figure 4-source data 2/Figure4A/Figure4A_Gel5_ATP6V0A1.tif]

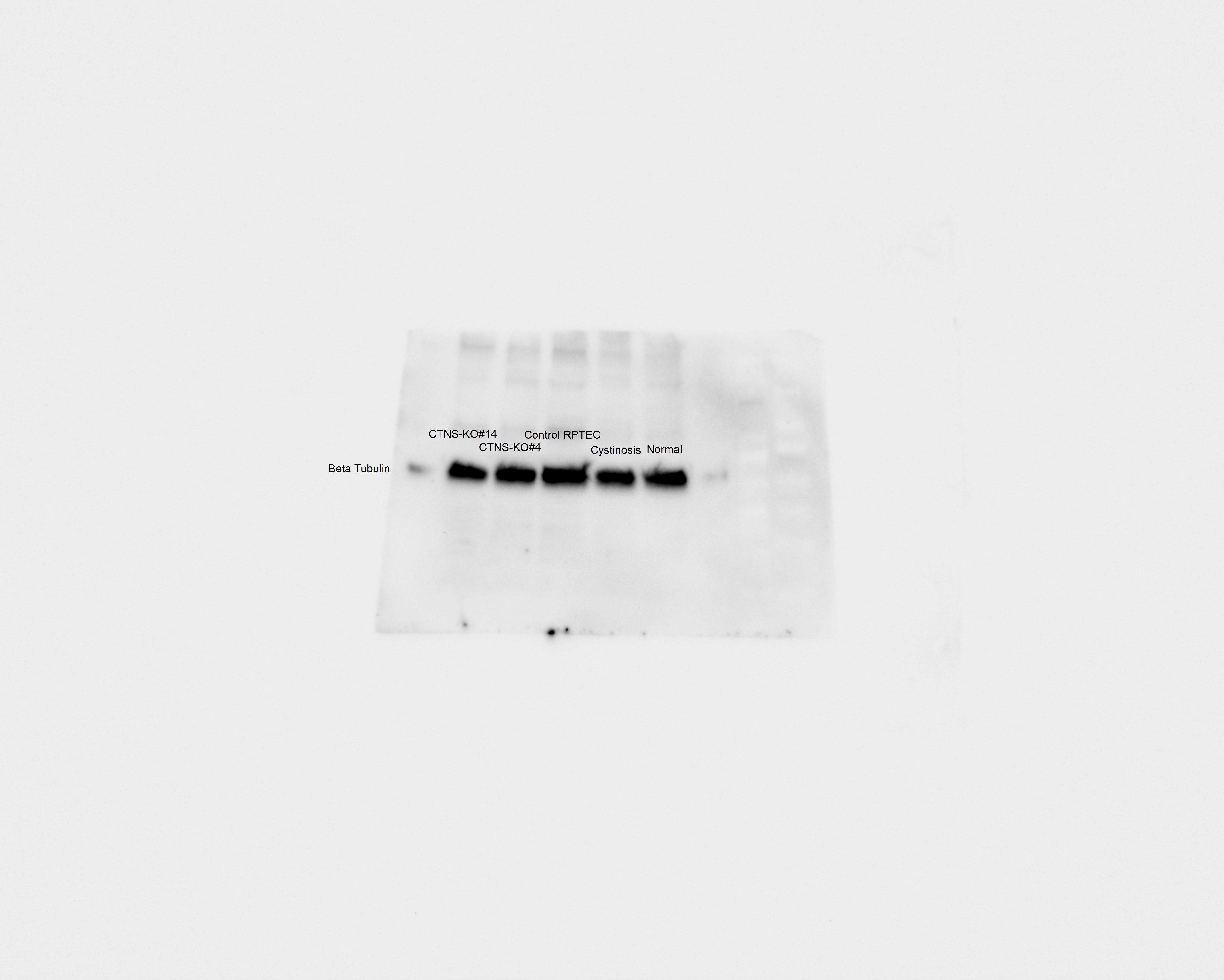

Supplement: Figure 4—source data 2. [file elife-94169-fig4-data2.zip › Figure 4-source data 2/Figure4A/Figure4A_Gel5_BetaTubulin.tif]

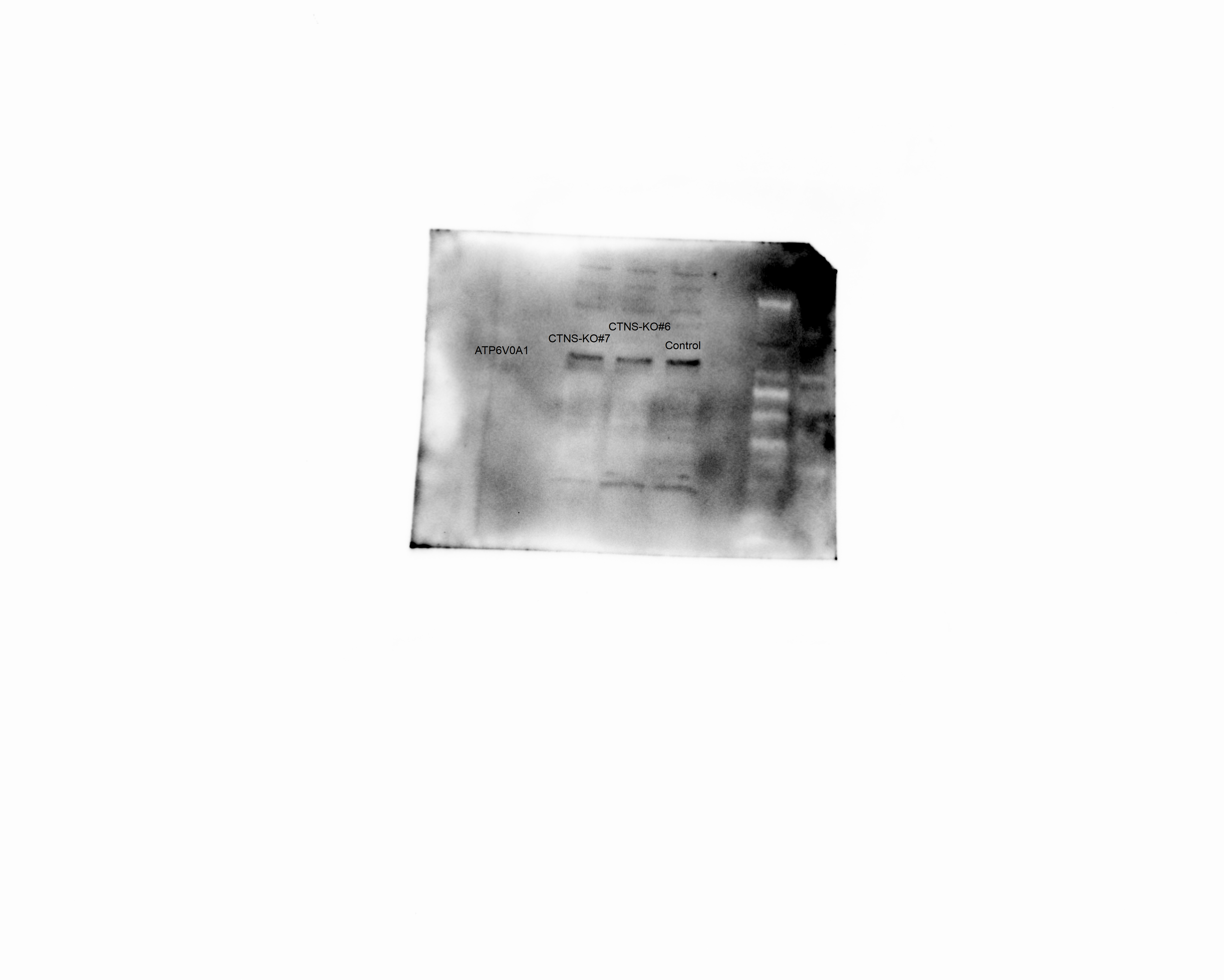

Supplement: Figure 4—source data 2. [file elife-94169-fig4-data2.zip › Figure 4-source data 2/Figure4B/Figure4B_Gel1_ATP6V0A1.tif]

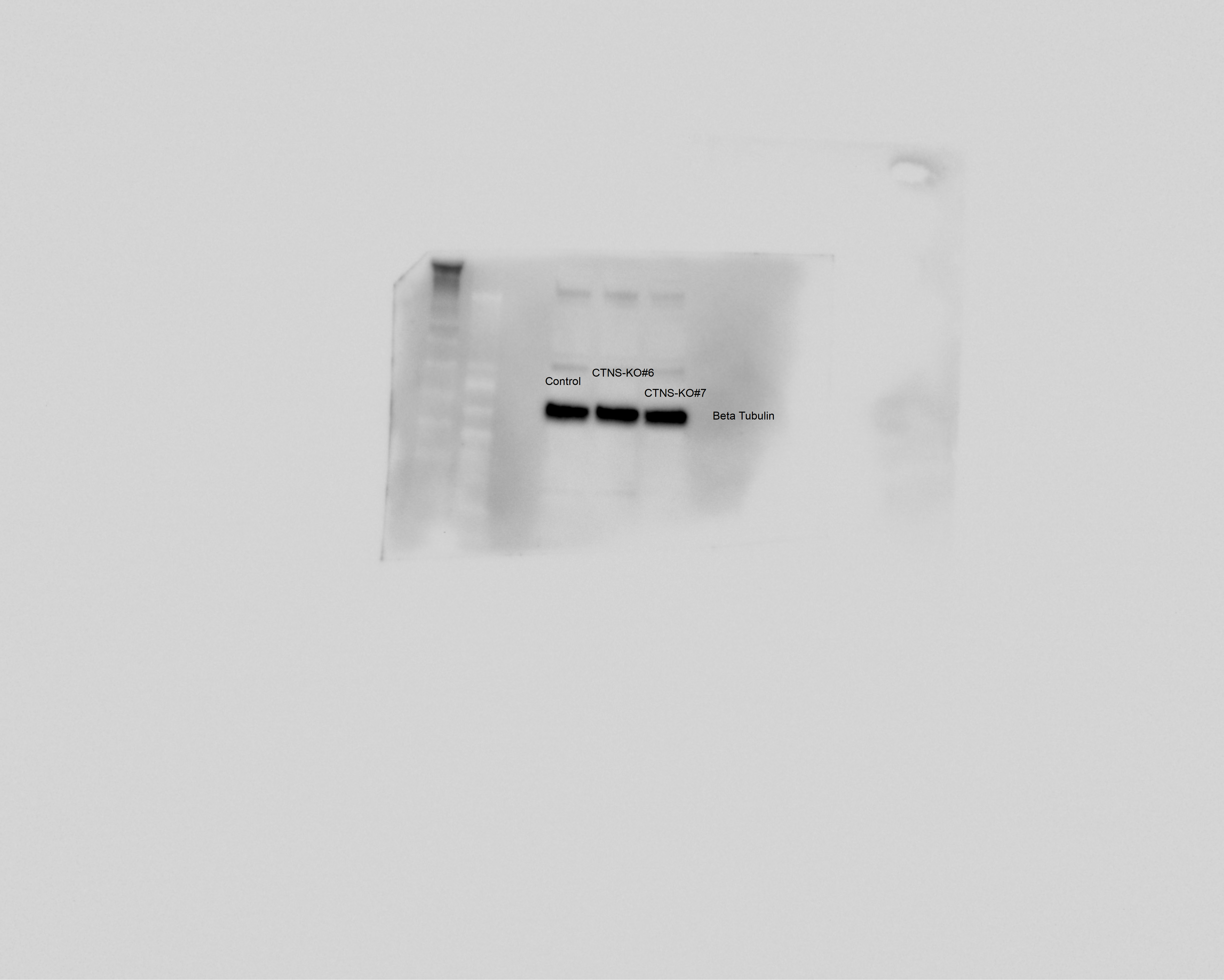

Supplement: Figure 4—source data 2. [file elife-94169-fig4-data2.zip › Figure 4-source data 2/Figure4B/Figure4B_Gel1_Beta Tubulin.tif]

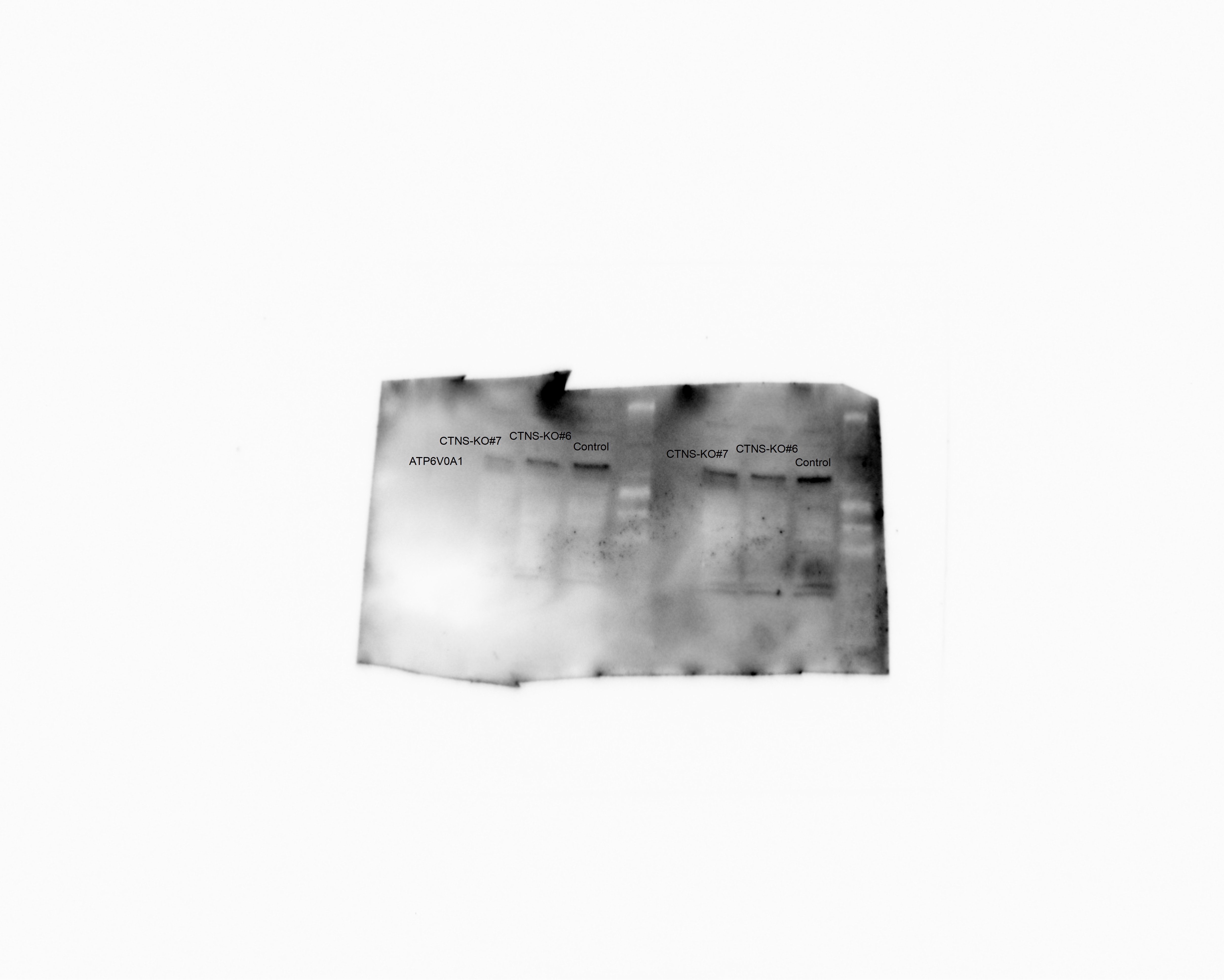

Supplement: Figure 4—source data 2. [file elife-94169-fig4-data2.zip › Figure 4-source data 2/Figure4B/Figure4B_Gel2and3_ATP6V0A1.tif]

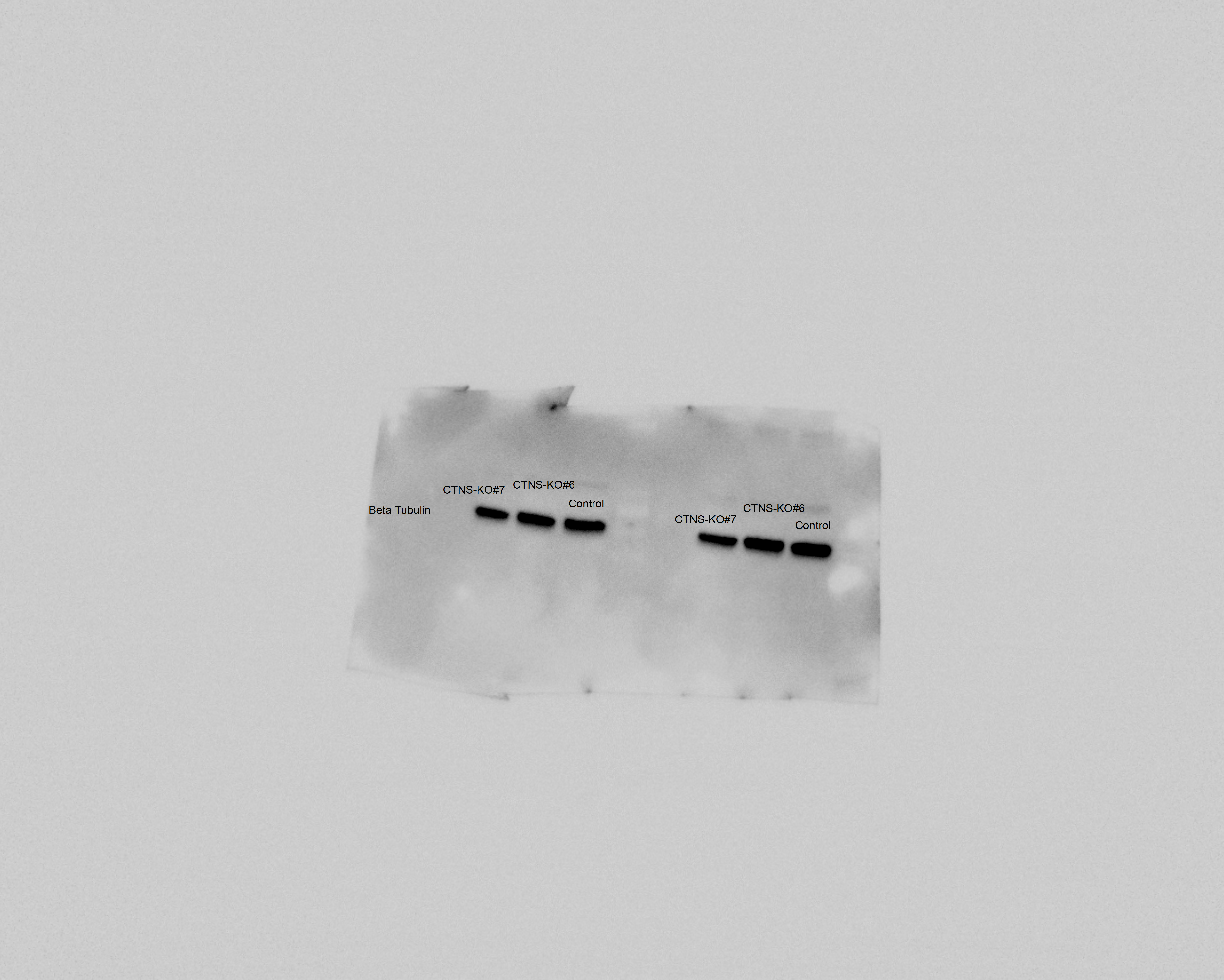

Supplement: Figure 4—source data 2. [file elife-94169-fig4-data2.zip › Figure 4-source data 2/Figure4B/Figure4B_Gel2and3_Beta Tubulin.tif]

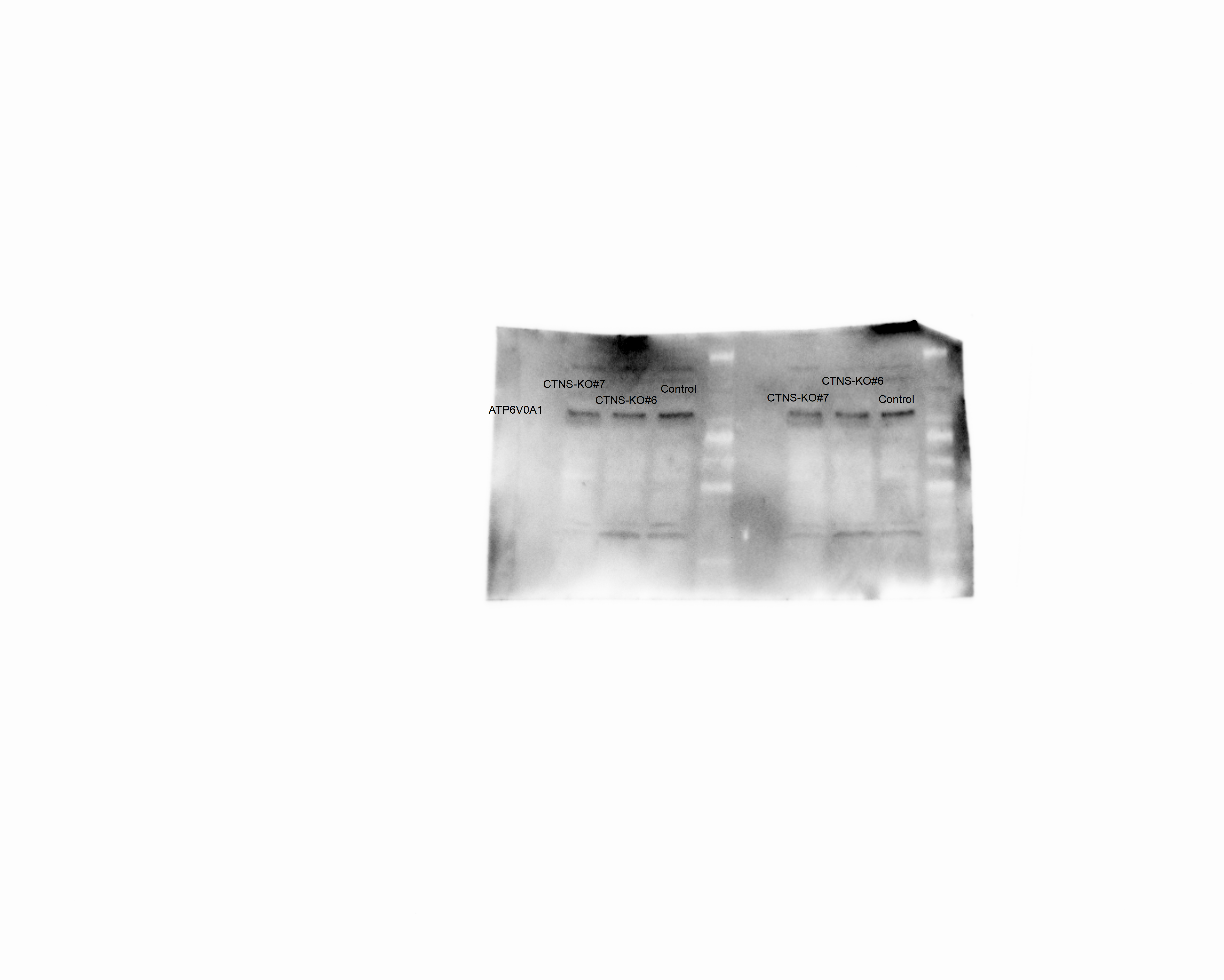

Supplement: Figure 4—source data 2. [file elife-94169-fig4-data2.zip › Figure 4-source data 2/Figure4B/Figure4B_Gel4and5_ATP6V0A1.tif]

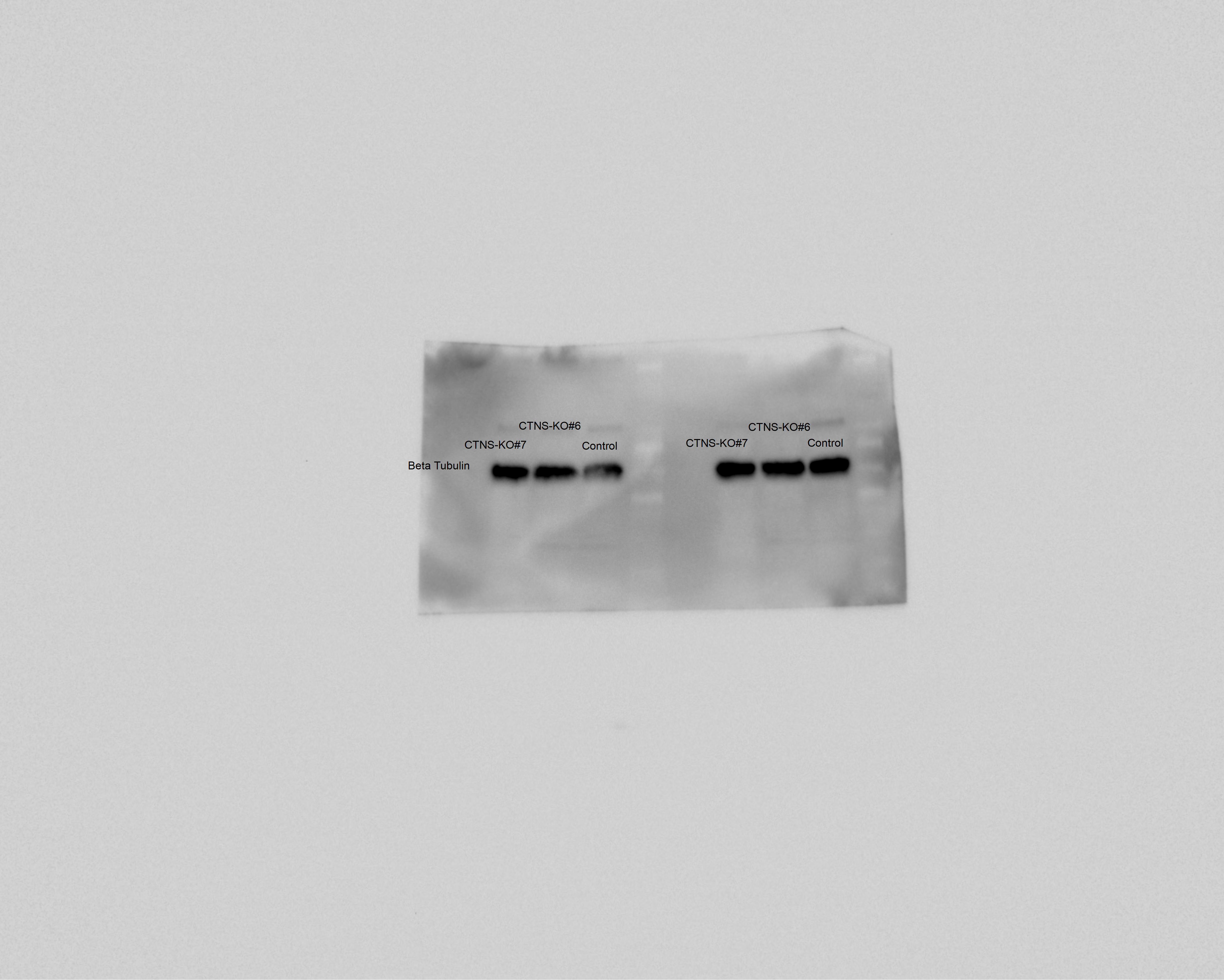

Supplement: Figure 4—source data 2. [file elife-94169-fig4-data2.zip › Figure 4-source data 2/Figure4B/Figure4B_Gel4and5_Beta Tubulin.tif]

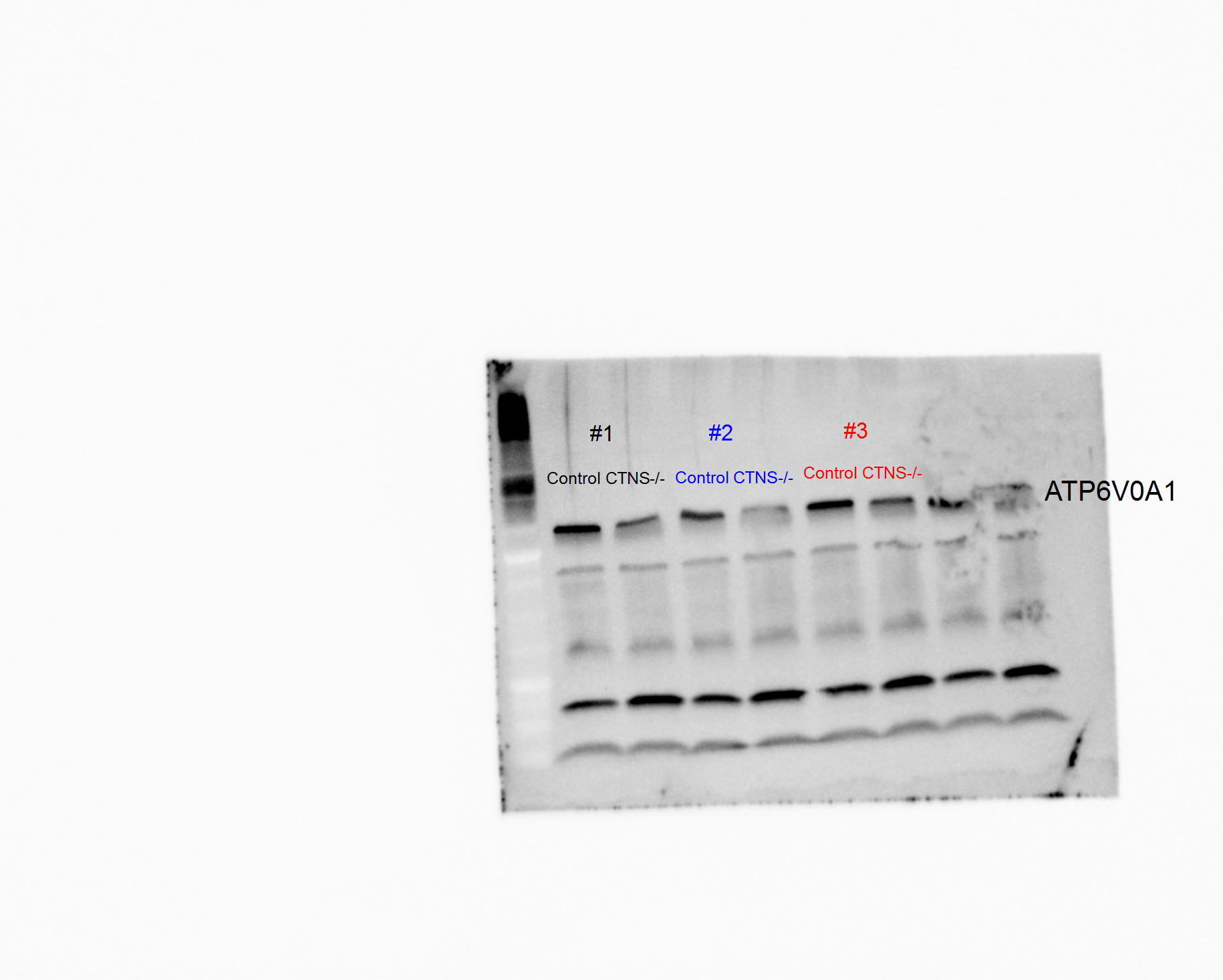

Supplement: Figure 4—source data 2. [file elife-94169-fig4-data2.zip › Figure 4-source data 2/Figure4C/4C_Gel 1,2,3_ATP6V0A1.tif]

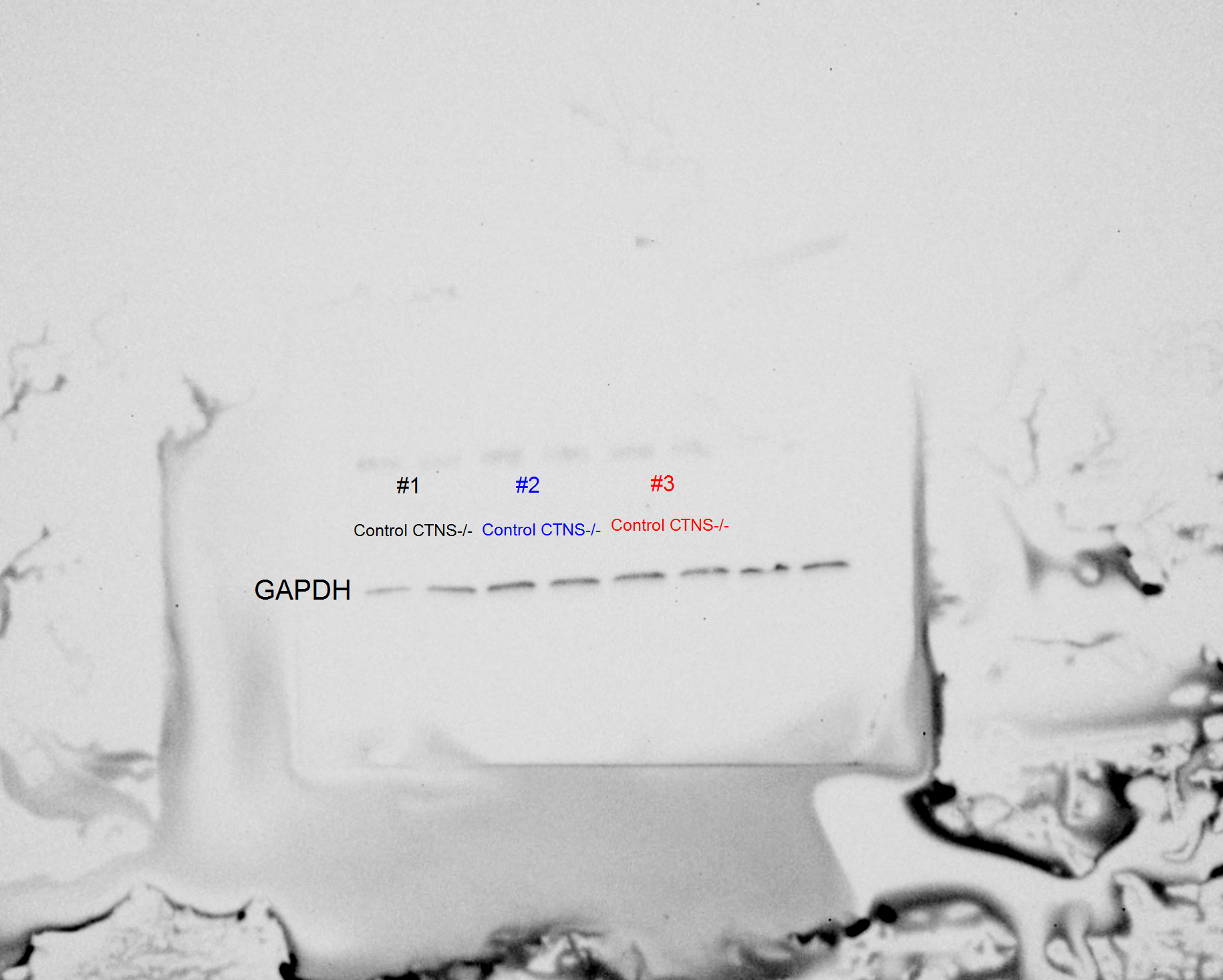

Supplement: Figure 4—source data 2. [file elife-94169-fig4-data2.zip › Figure 4-source data 2/Figure4C/4C_Gel 1,2,3_GAPDH.tif]

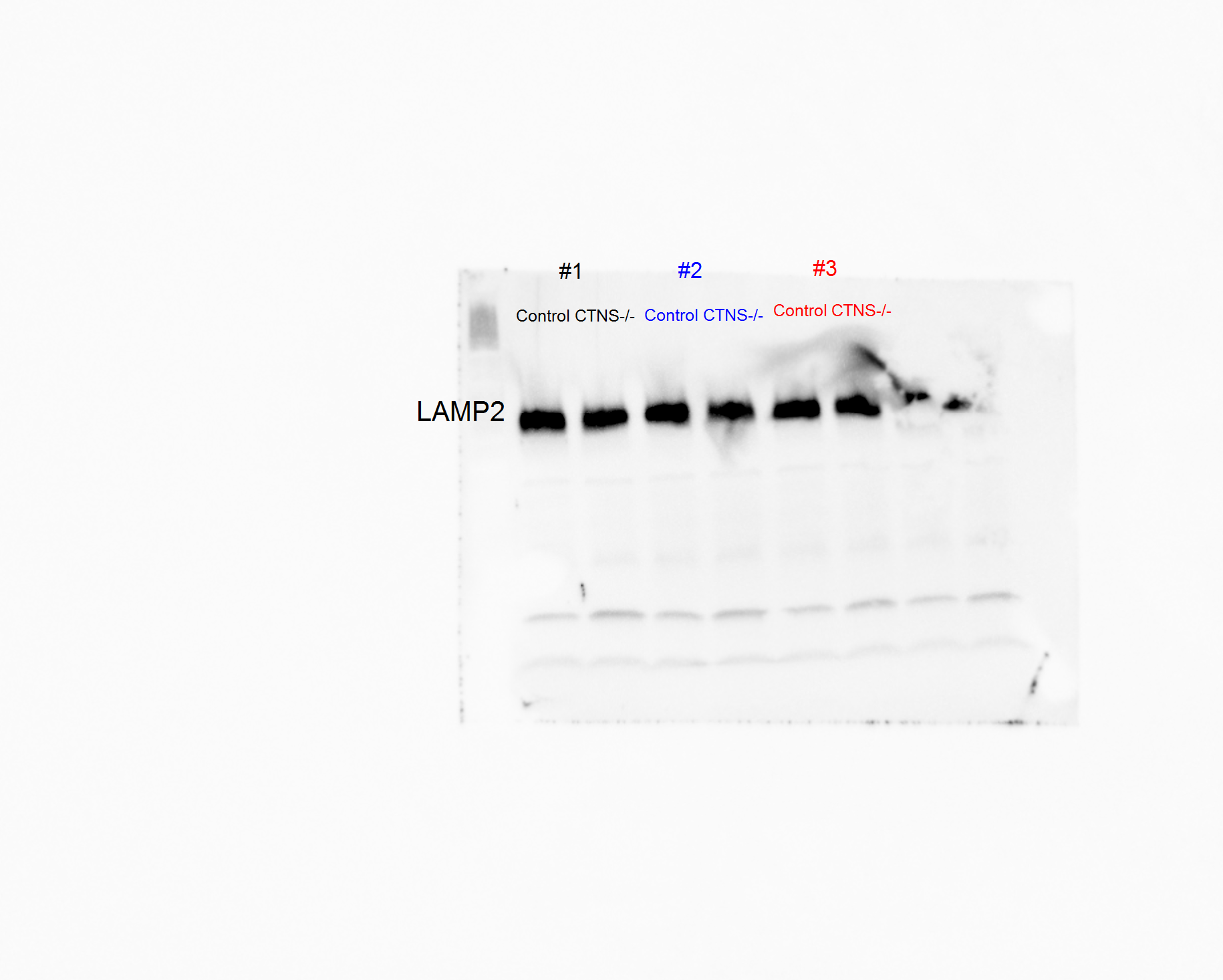

Supplement: Figure 4—source data 2. [file elife-94169-fig4-data2.zip › Figure 4-source data 2/Figure4C/4C_Gel 1,2,3_LAMP2.tif]

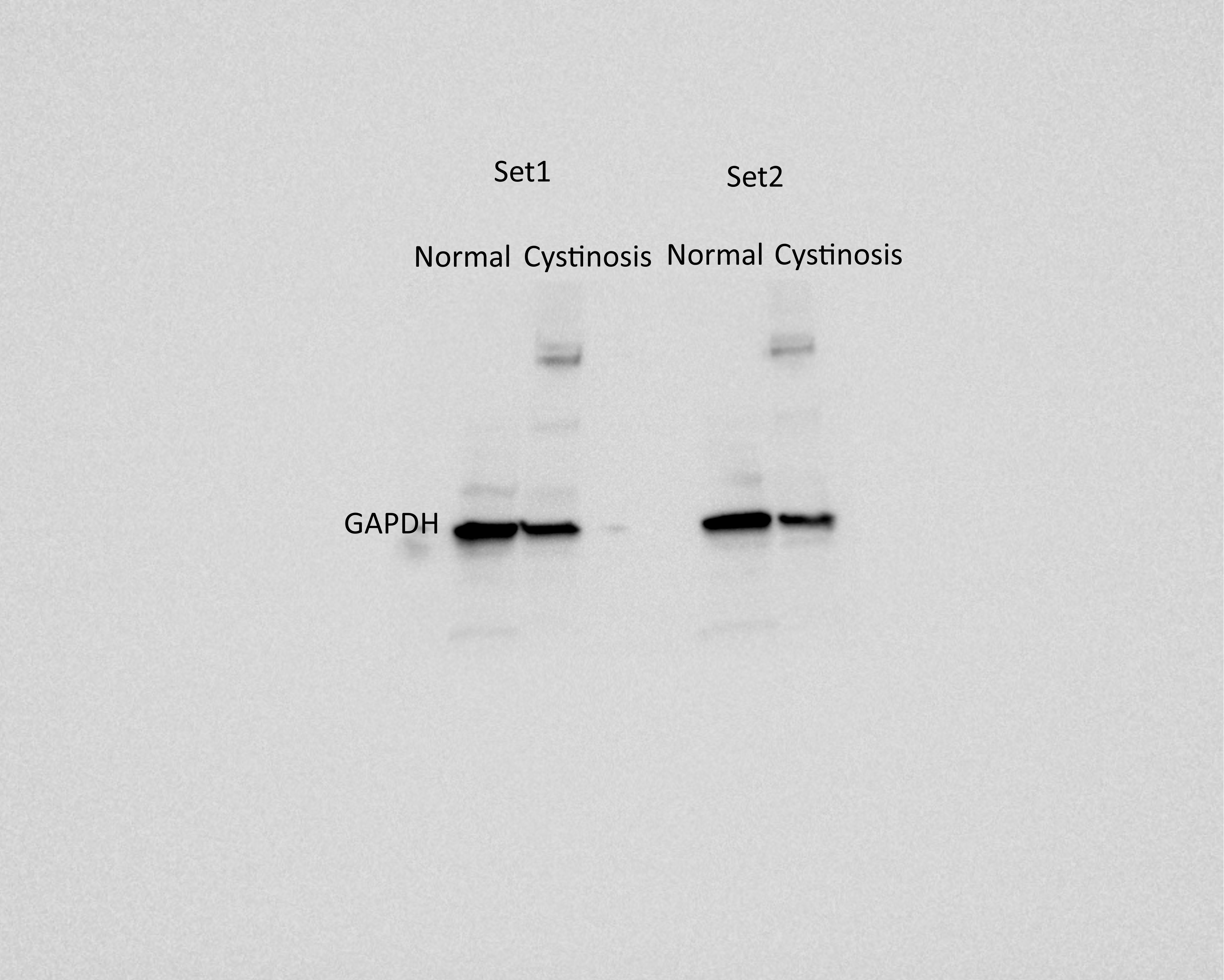

Supplement: Figure 5—source data 2. [file elife-94169-fig5-data2.zip › Figure 5-source data 2/Figure 5C/5C Gel 1_GAPDH.tif]

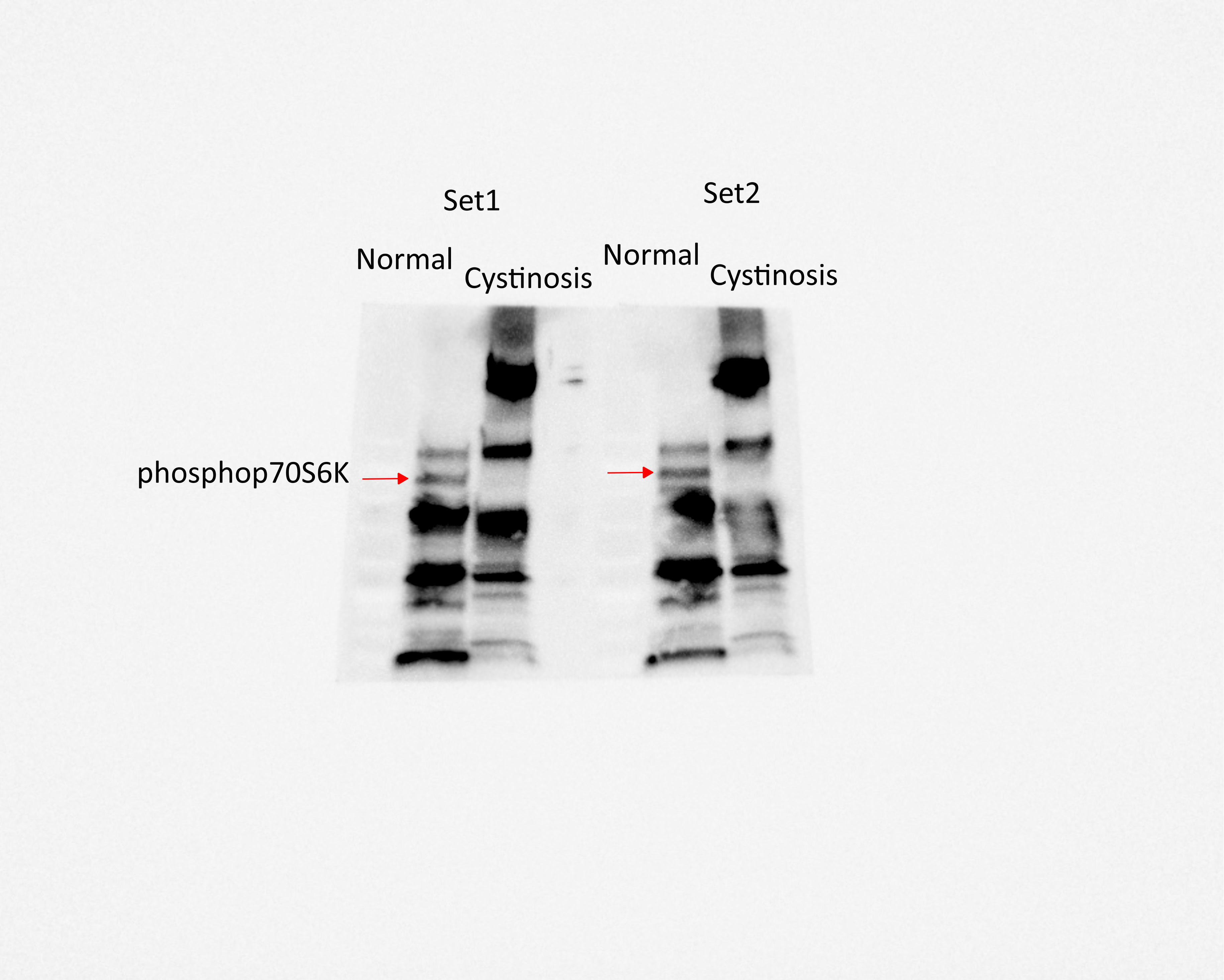

Supplement: Figure 5—source data 2. [file elife-94169-fig5-data2.zip › Figure 5-source data 2/Figure 5C/5C Gel 1_Phosphop70S6K.tif]

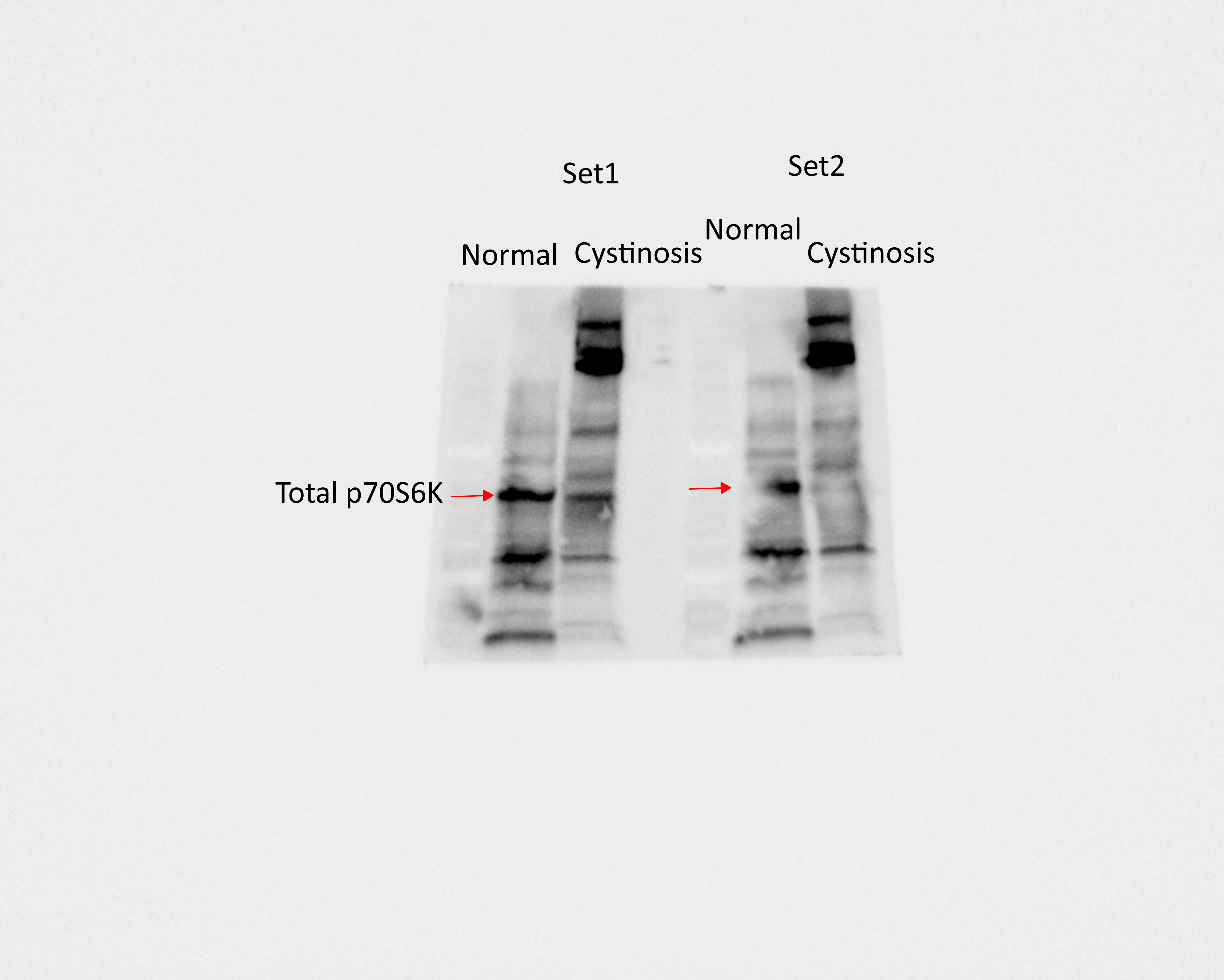

Supplement: Figure 5—source data 2. [file elife-94169-fig5-data2.zip › Figure 5-source data 2/Figure 5C/5C Gel 1_Totalp70S6K.tif]

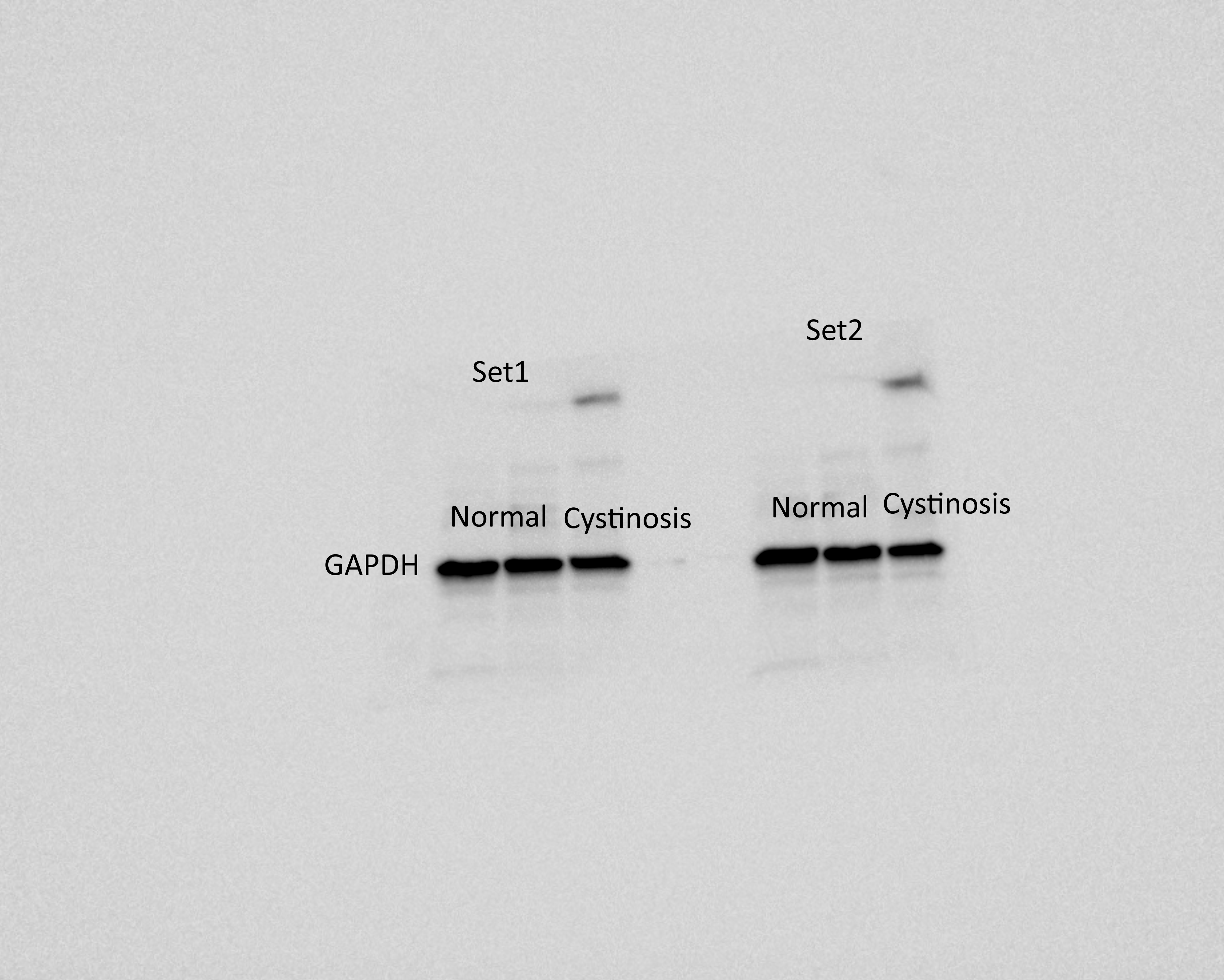

Supplement: Figure 5—source data 2. [file elife-94169-fig5-data2.zip › Figure 5-source data 2/Figure 5C/5C Gel 2_GAPDH.tif]

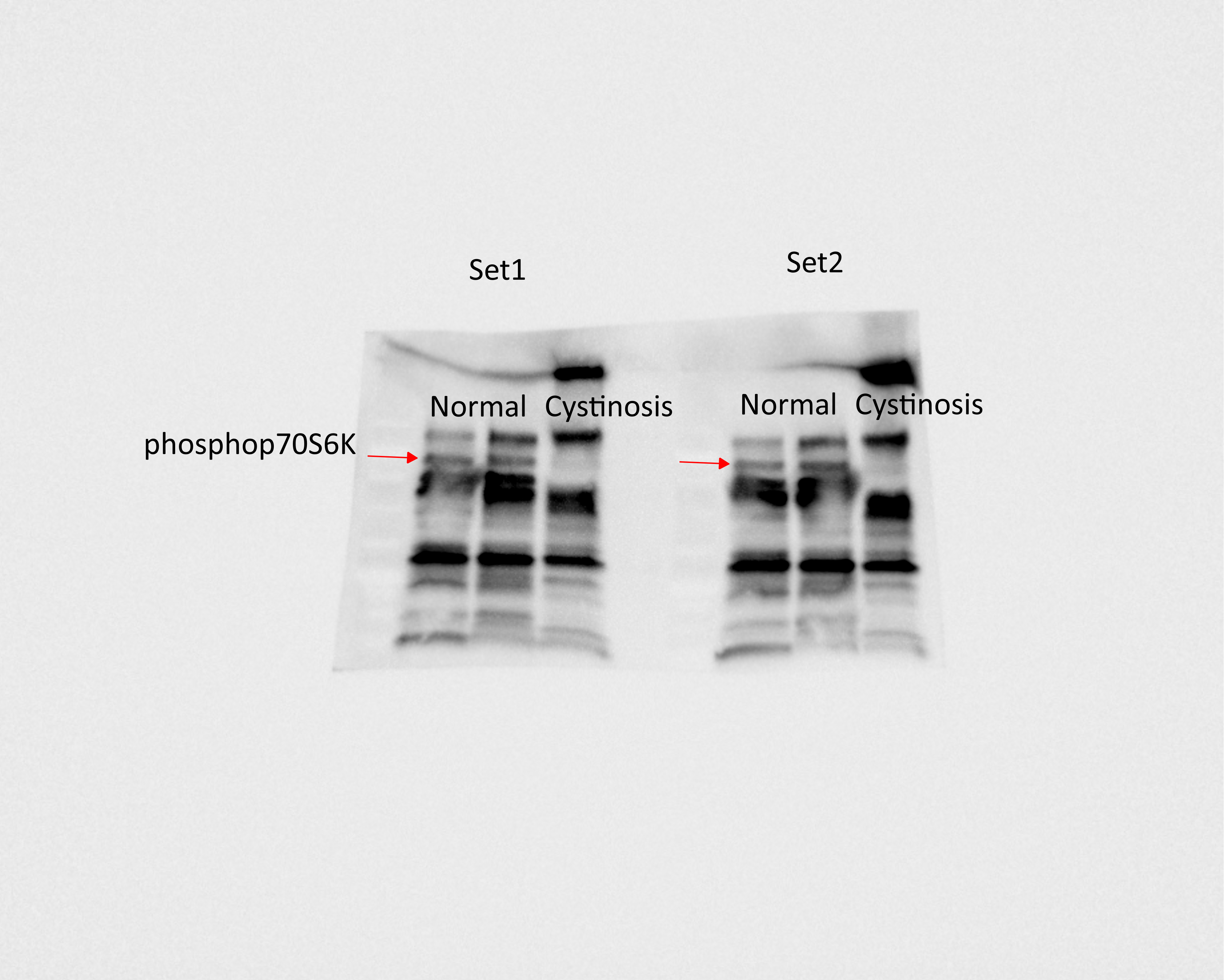

Supplement: Figure 5—source data 2. [file elife-94169-fig5-data2.zip › Figure 5-source data 2/Figure 5C/5C Gel 2_Phosphop70S6K.tif]

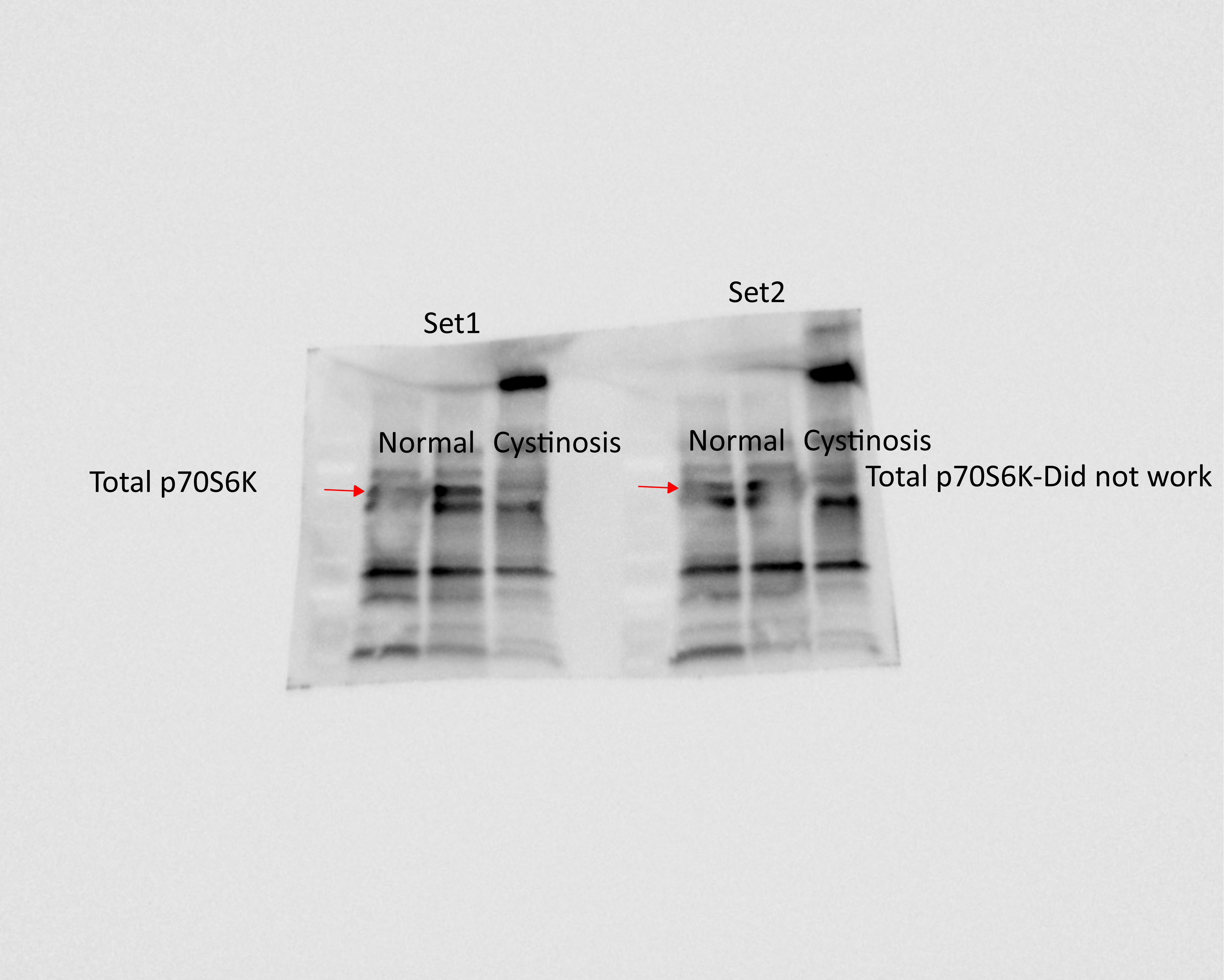

Supplement: Figure 5—source data 2. [file elife-94169-fig5-data2.zip › Figure 5-source data 2/Figure 5C/5C Gel 2_Totalp70S6K.tif]

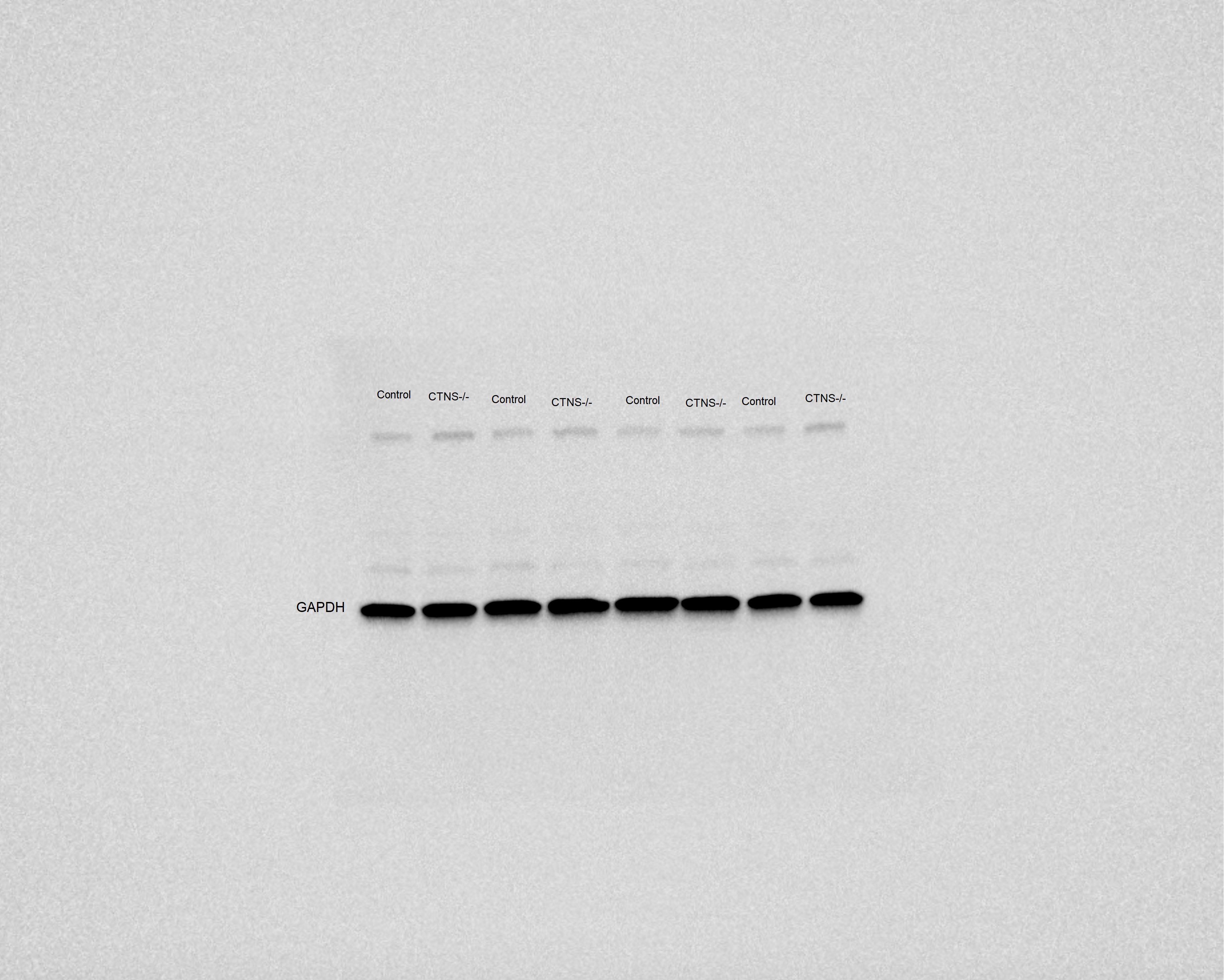

Supplement: Figure 5—source data 2. [file elife-94169-fig5-data2.zip › Figure 5-source data 2/Figure 5D/Figure5D_GAPDH.tif]

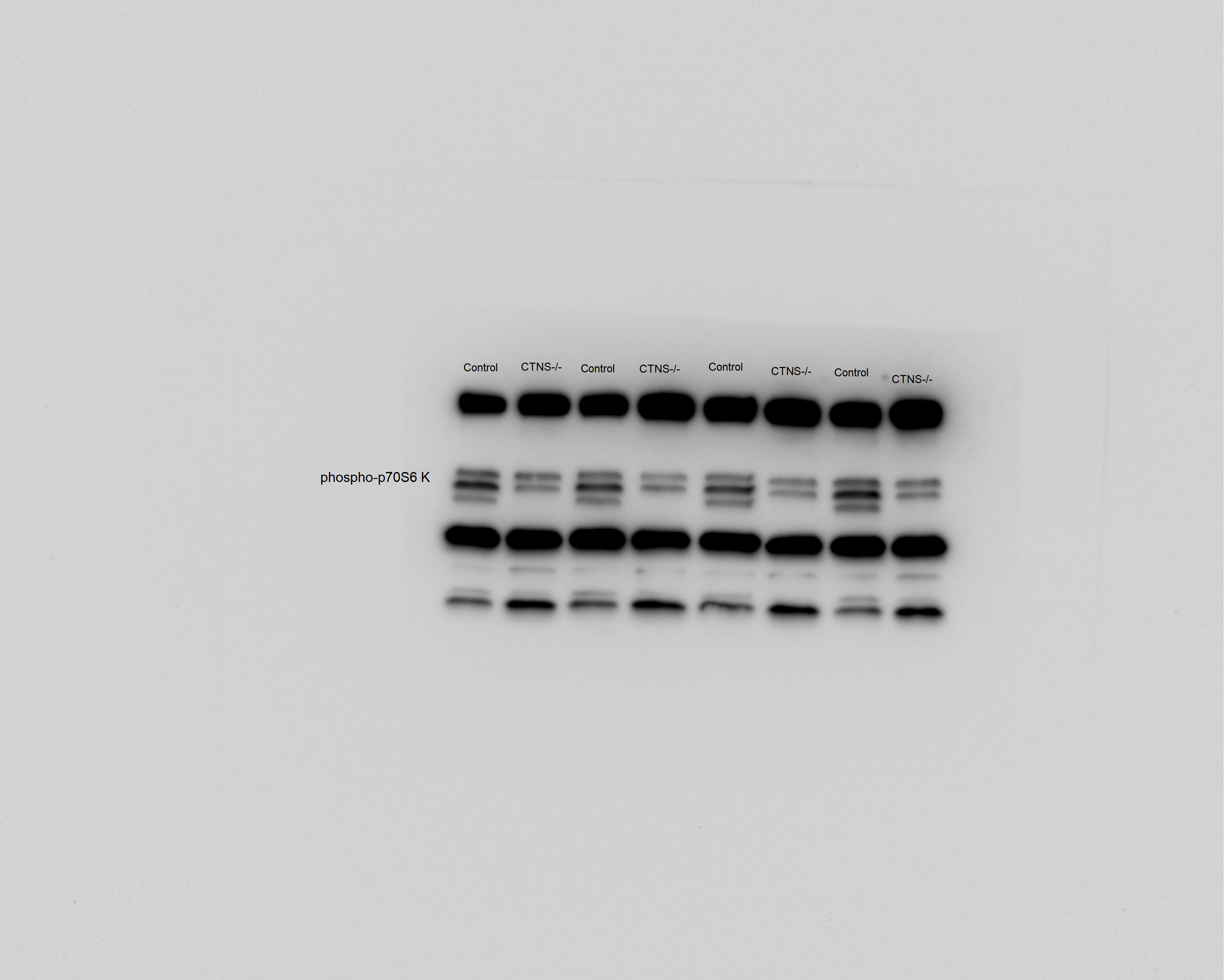

Supplement: Figure 5—source data 2. [file elife-94169-fig5-data2.zip › Figure 5-source data 2/Figure 5D/Figure5D_phosphop70S6K.tif]

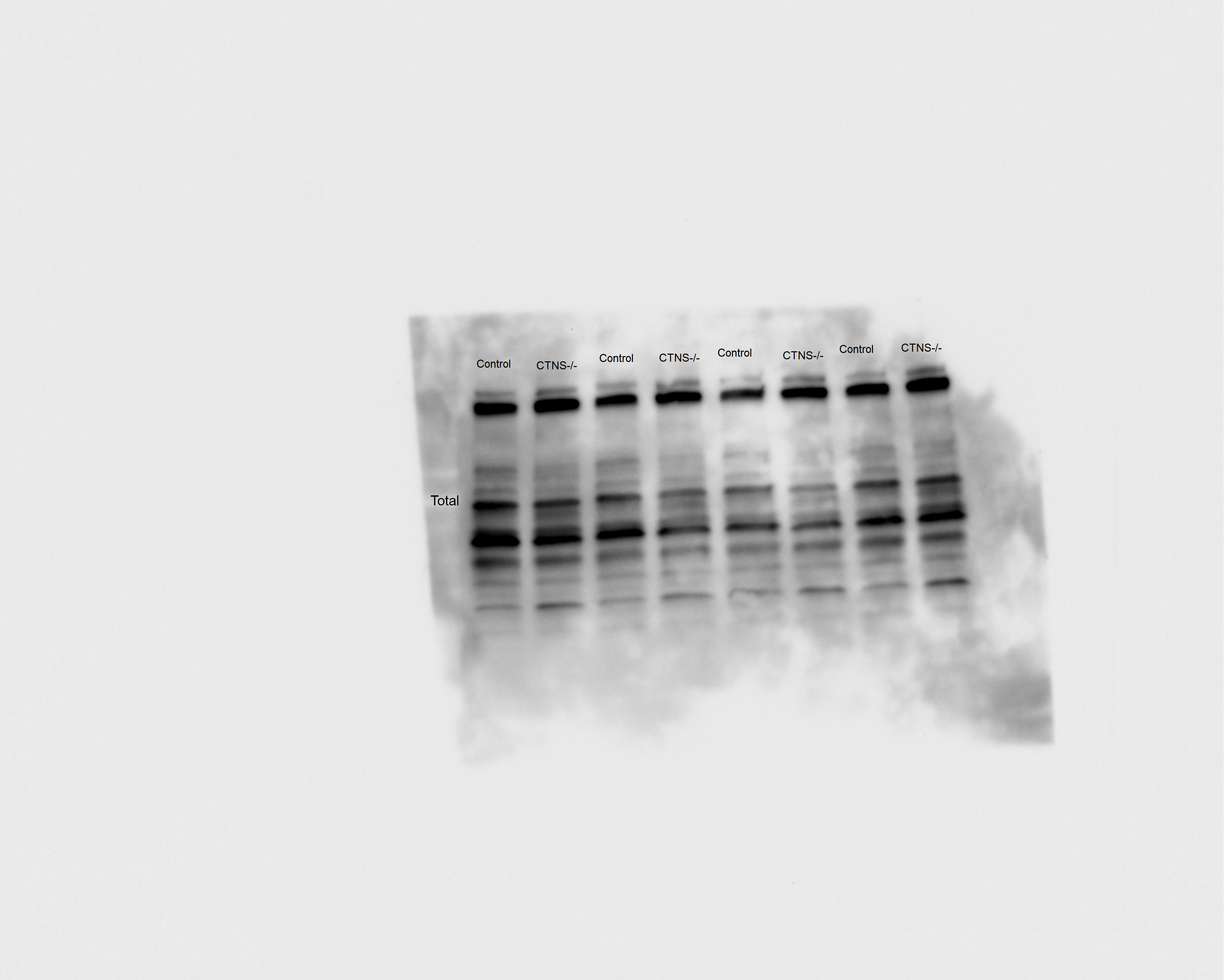

Supplement: Figure 5—source data 2. [file elife-94169-fig5-data2.zip › Figure 5-source data 2/Figure 5D/Figure5D_Total p70S6K.tif]

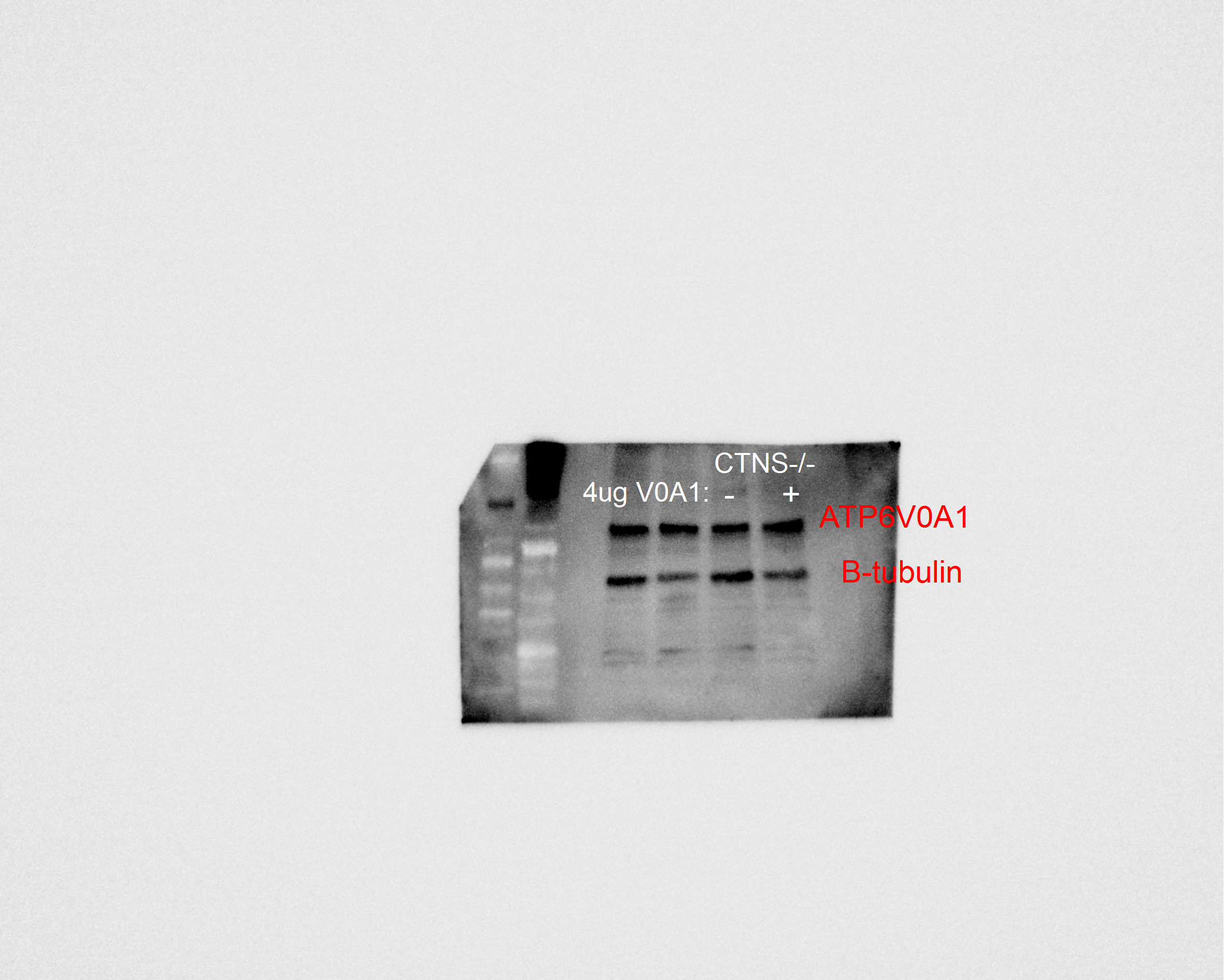

Supplement: Figure 7—source data 2. [file elife-94169-fig7-data2.zip › Figure 7-source data 2/Figure7A/ATP6V0A1/7A - Gel 1_ATP6V0A1_B-tubulin.tif]

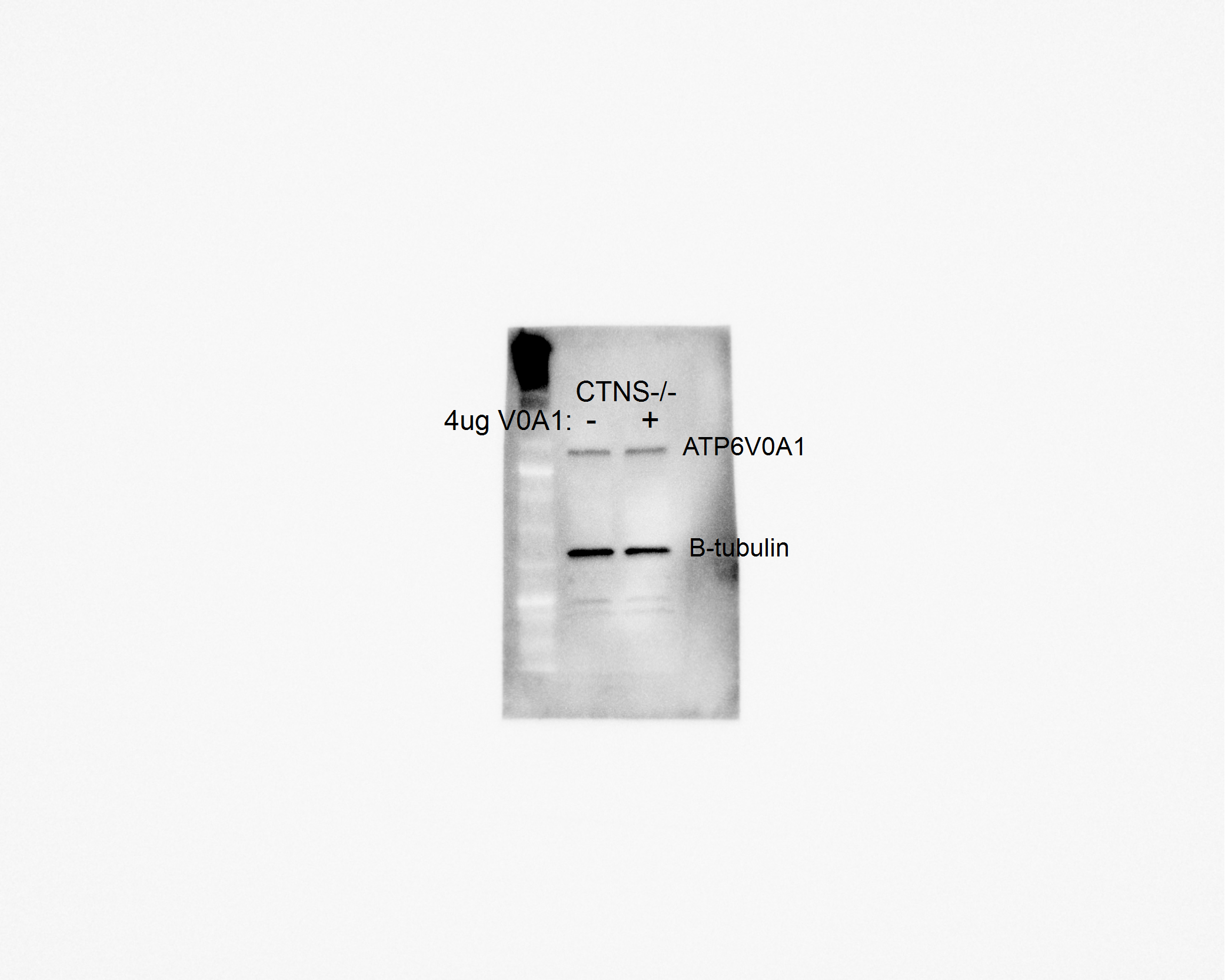

Supplement: Figure 7—source data 2. [file elife-94169-fig7-data2.zip › Figure 7-source data 2/Figure7A/ATP6V0A1/7A - Gel 2_ATP6V0A1_B-tubulin.tif]

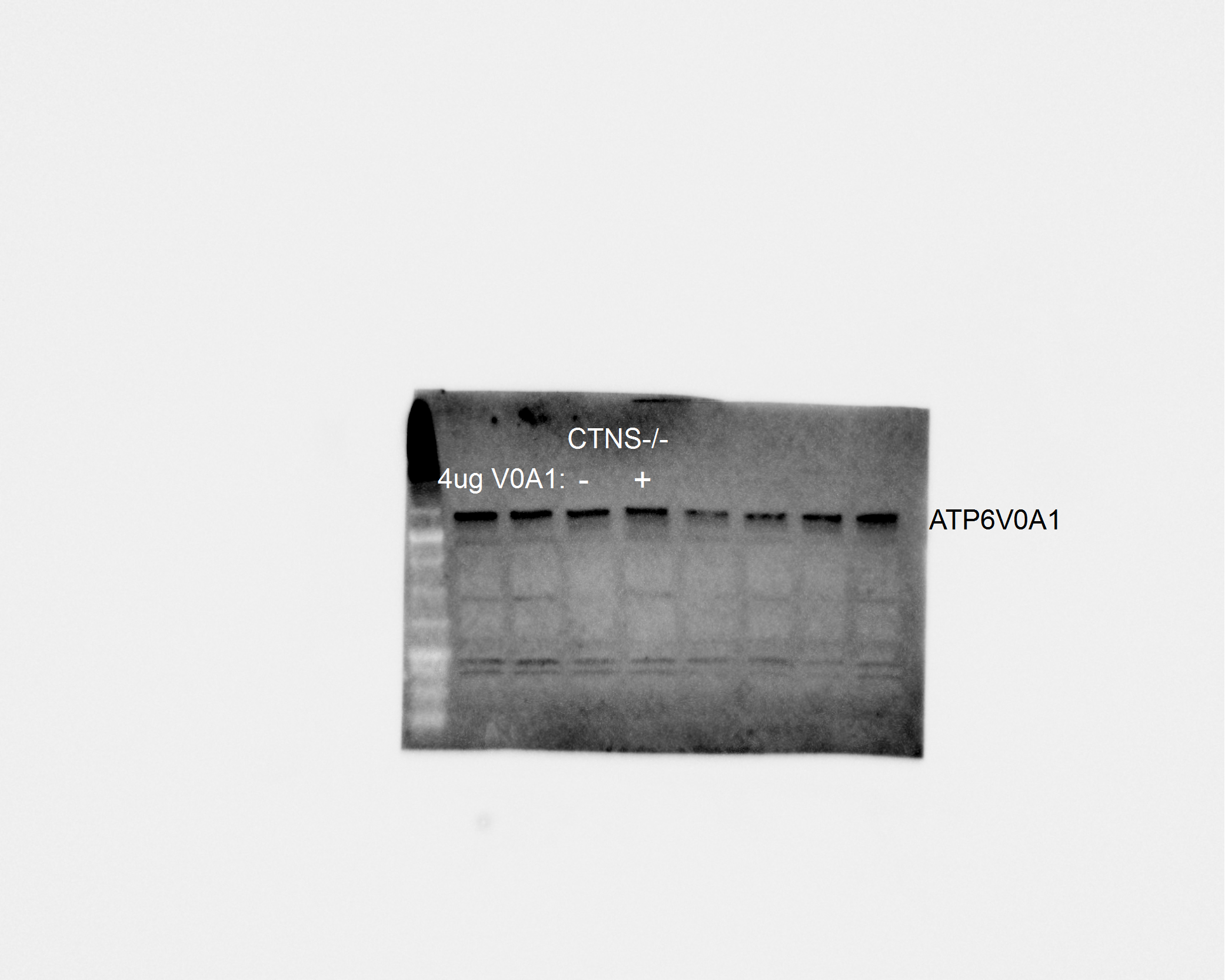

Supplement: Figure 7—source data 2. [file elife-94169-fig7-data2.zip › Figure 7-source data 2/Figure7A/ATP6V0A1/7A - Gel 3_ATP6V0A1.tif]

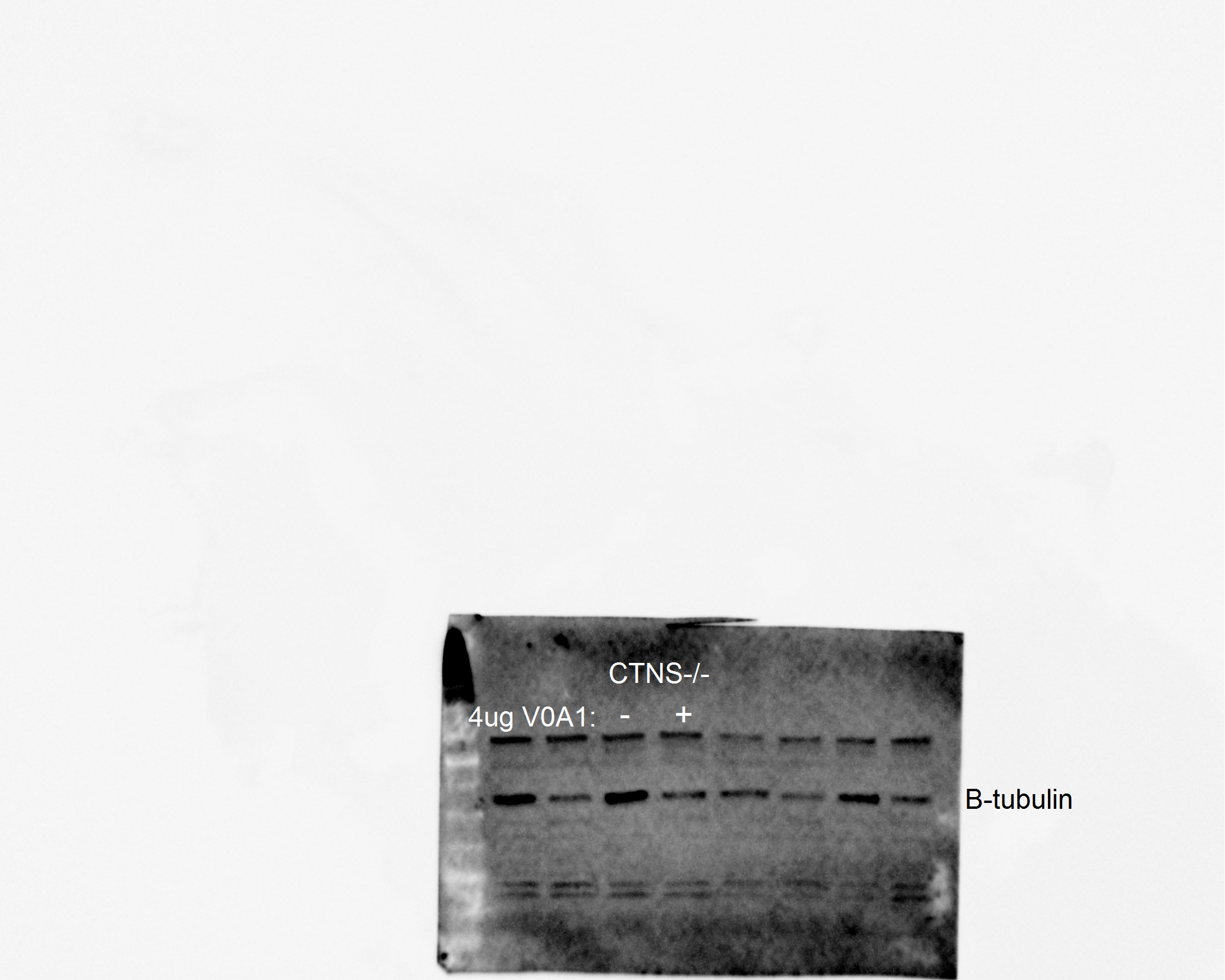

Supplement: Figure 7—source data 2. [file elife-94169-fig7-data2.zip › Figure 7-source data 2/Figure7A/ATP6V0A1/7A - Gel 3_B-tubulin.tif]

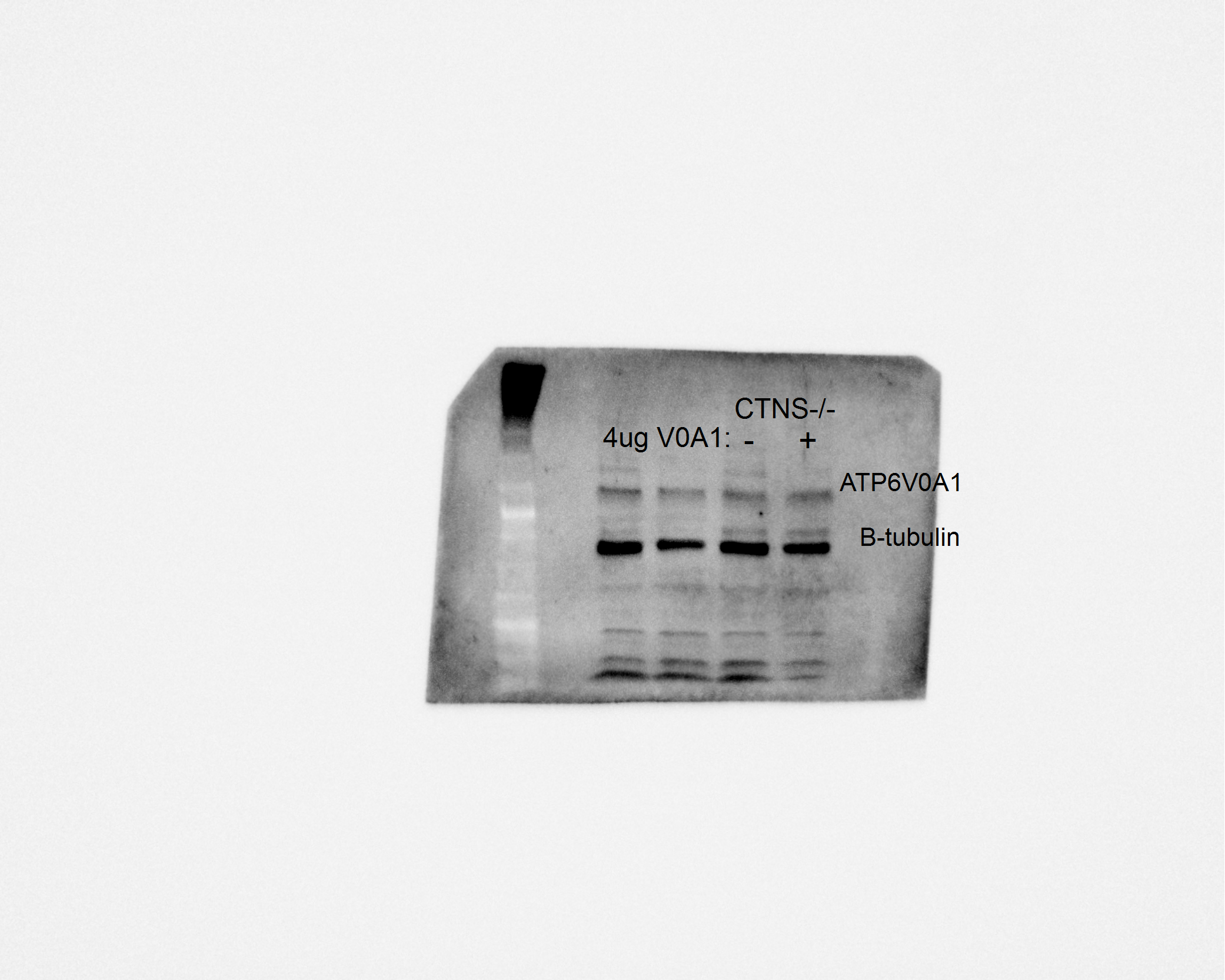

Supplement: Figure 7—source data 2. [file elife-94169-fig7-data2.zip › Figure 7-source data 2/Figure7A/ATP6V0A1/7A - Gel 4_ATP6V0A1_B-tubulin.tif]

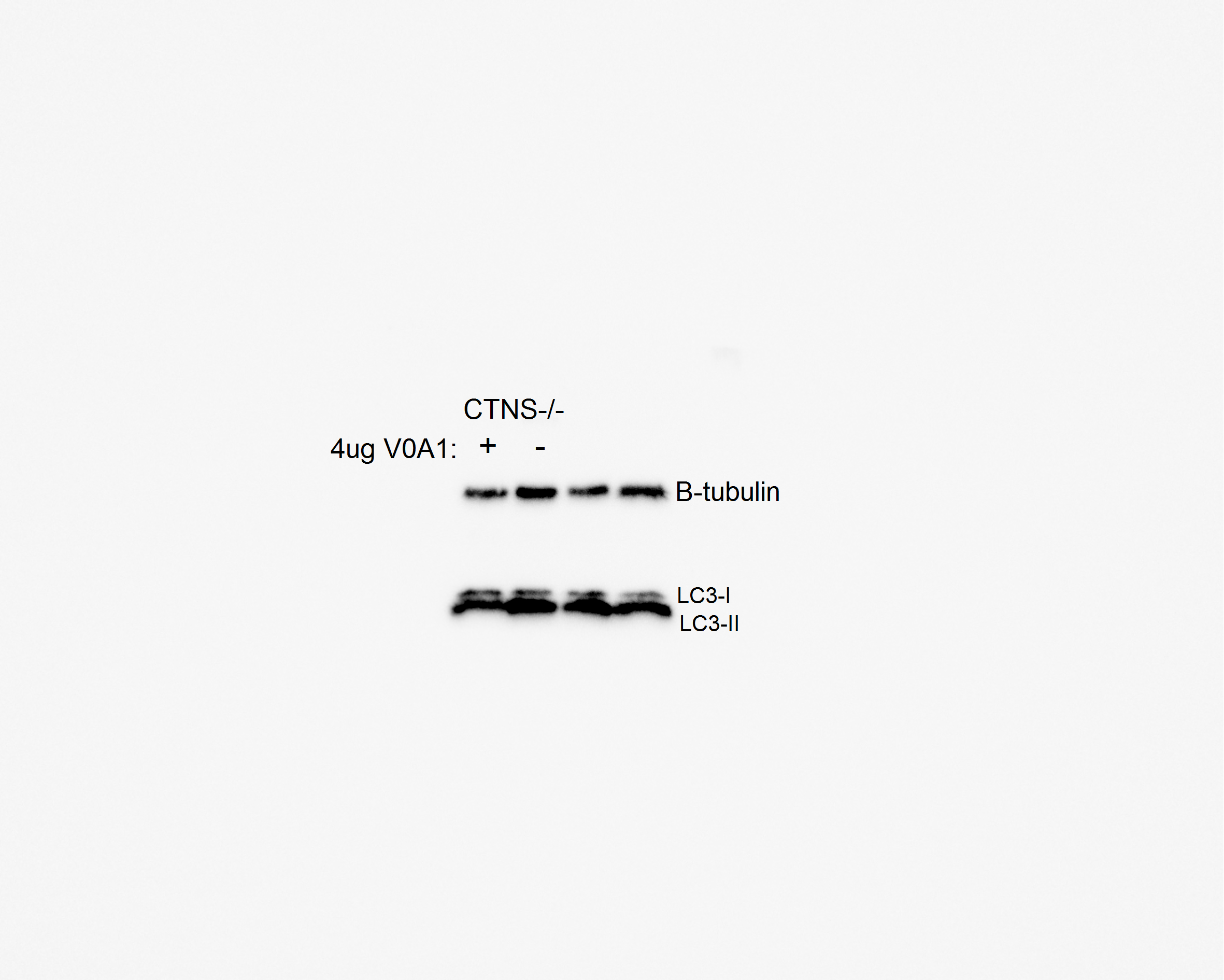

Supplement: Figure 7—source data 2. [file elife-94169-fig7-data2.zip › Figure 7-source data 2/Figure7A/LC3/7A - Gel 1_LC3_B-tubulin.tif]

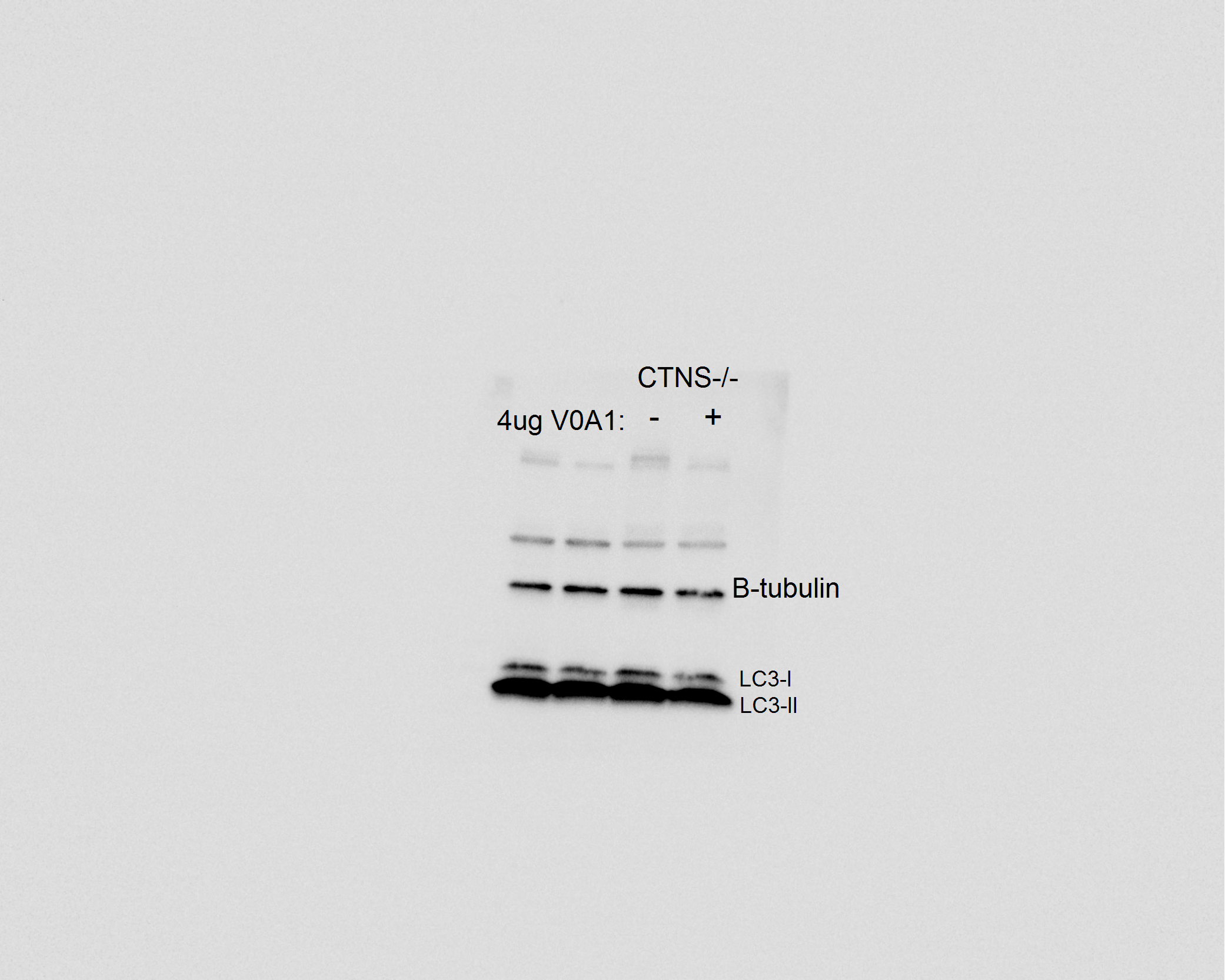

Supplement: Figure 7—source data 2. [file elife-94169-fig7-data2.zip › Figure 7-source data 2/Figure7A/LC3/7A - Gel 2_LC3_B-tubulin.tif]

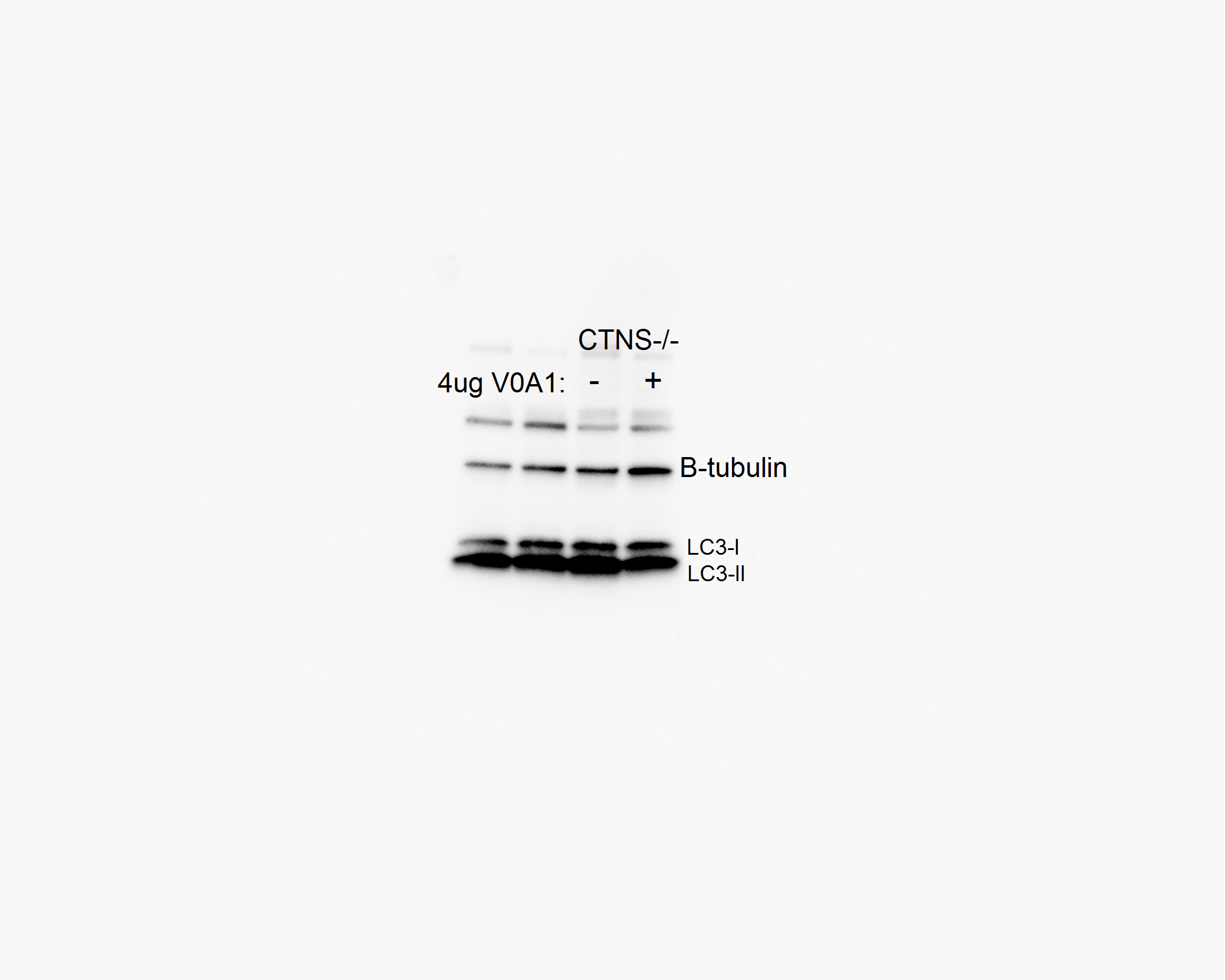

Supplement: Figure 7—source data 2. [file elife-94169-fig7-data2.zip › Figure 7-source data 2/Figure7A/LC3/7A - Gel 3_LC3_B-tubulin.tif]

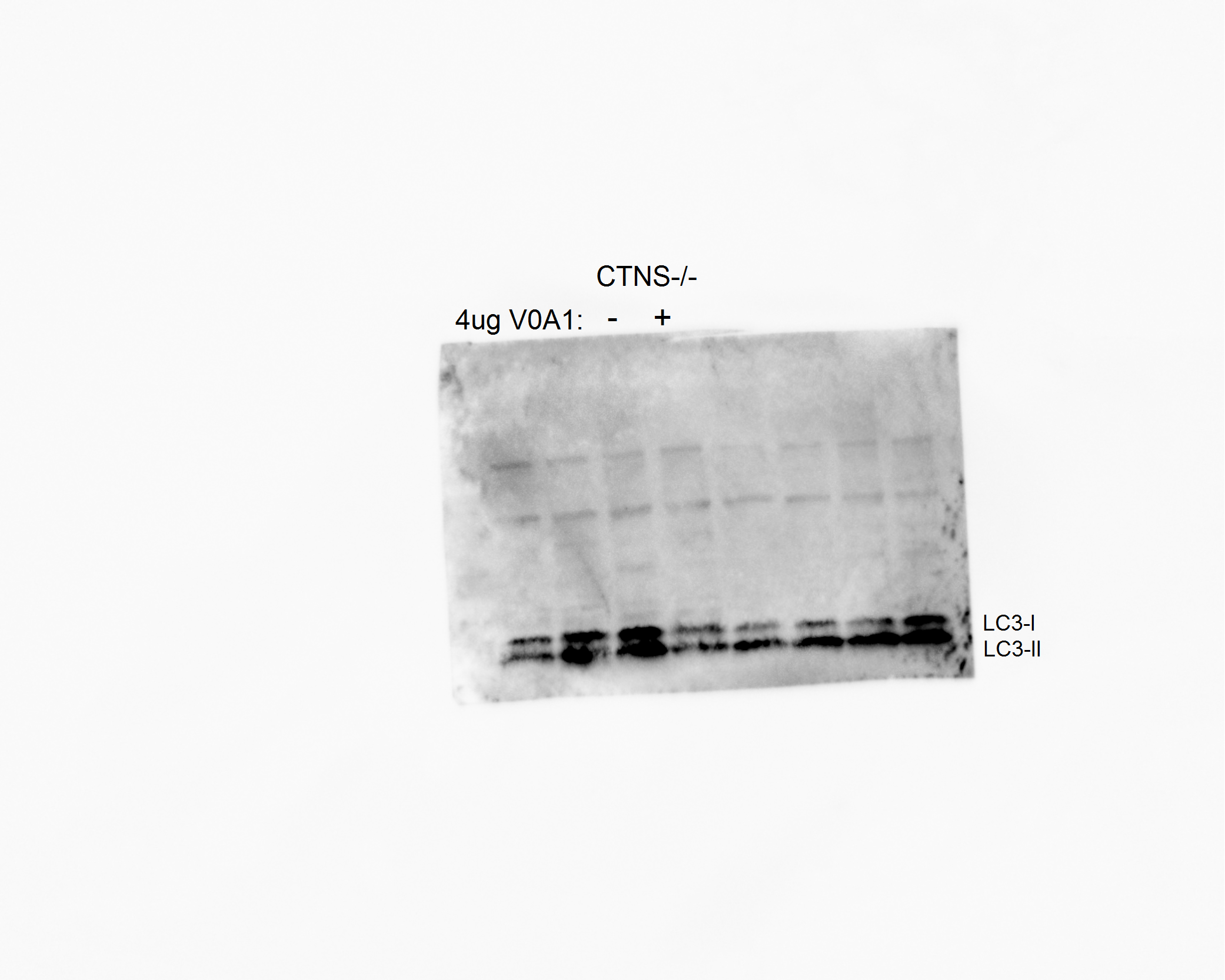

Supplement: Figure 7—source data 2. [file elife-94169-fig7-data2.zip › Figure 7-source data 2/Figure7A/LC3/7A - Gel 4_LC3.tif]

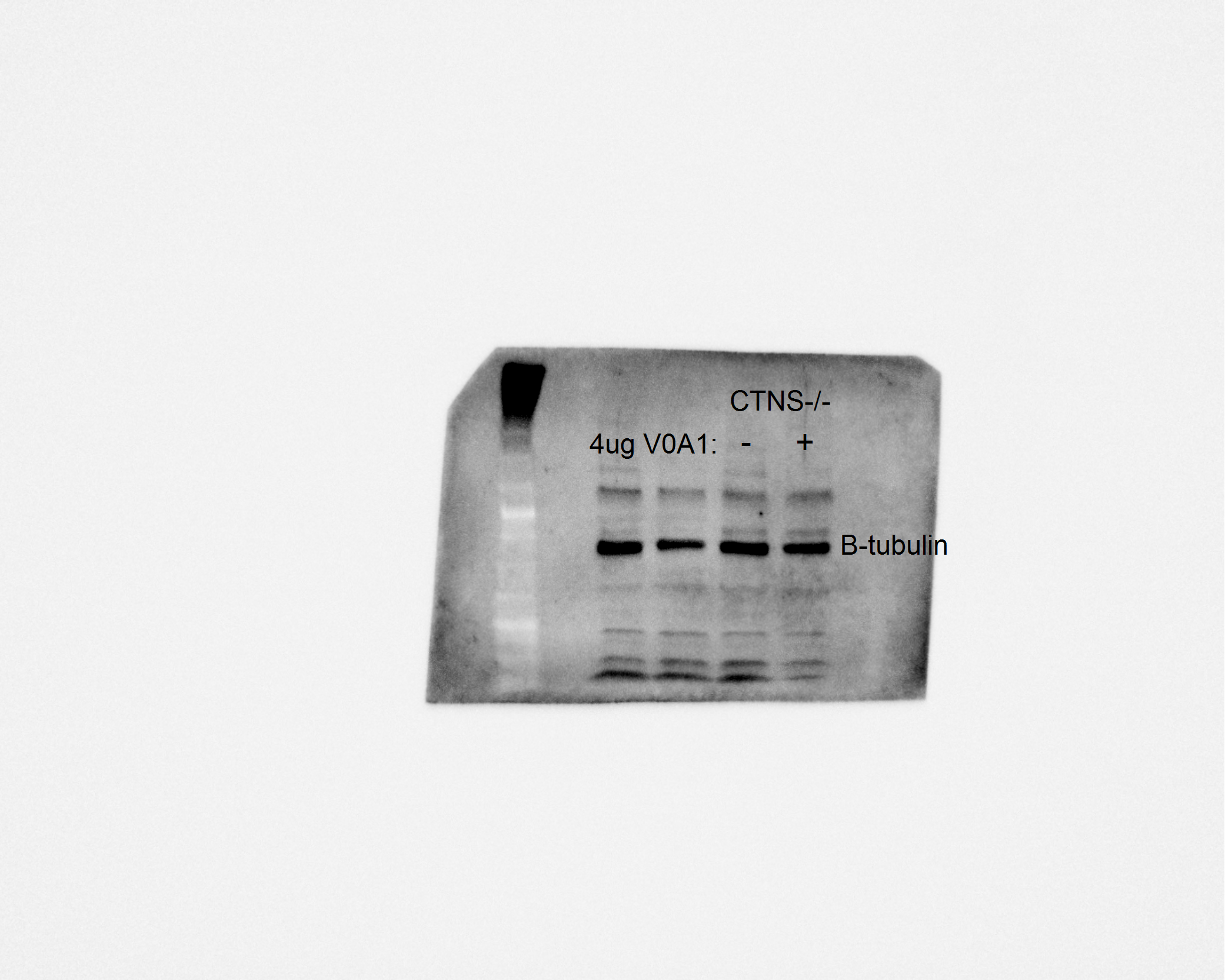

Supplement: Figure 7—source data 2. [file elife-94169-fig7-data2.zip › Figure 7-source data 2/Figure7A/LC3/7A - Gel 5_B-tubulin.tif]

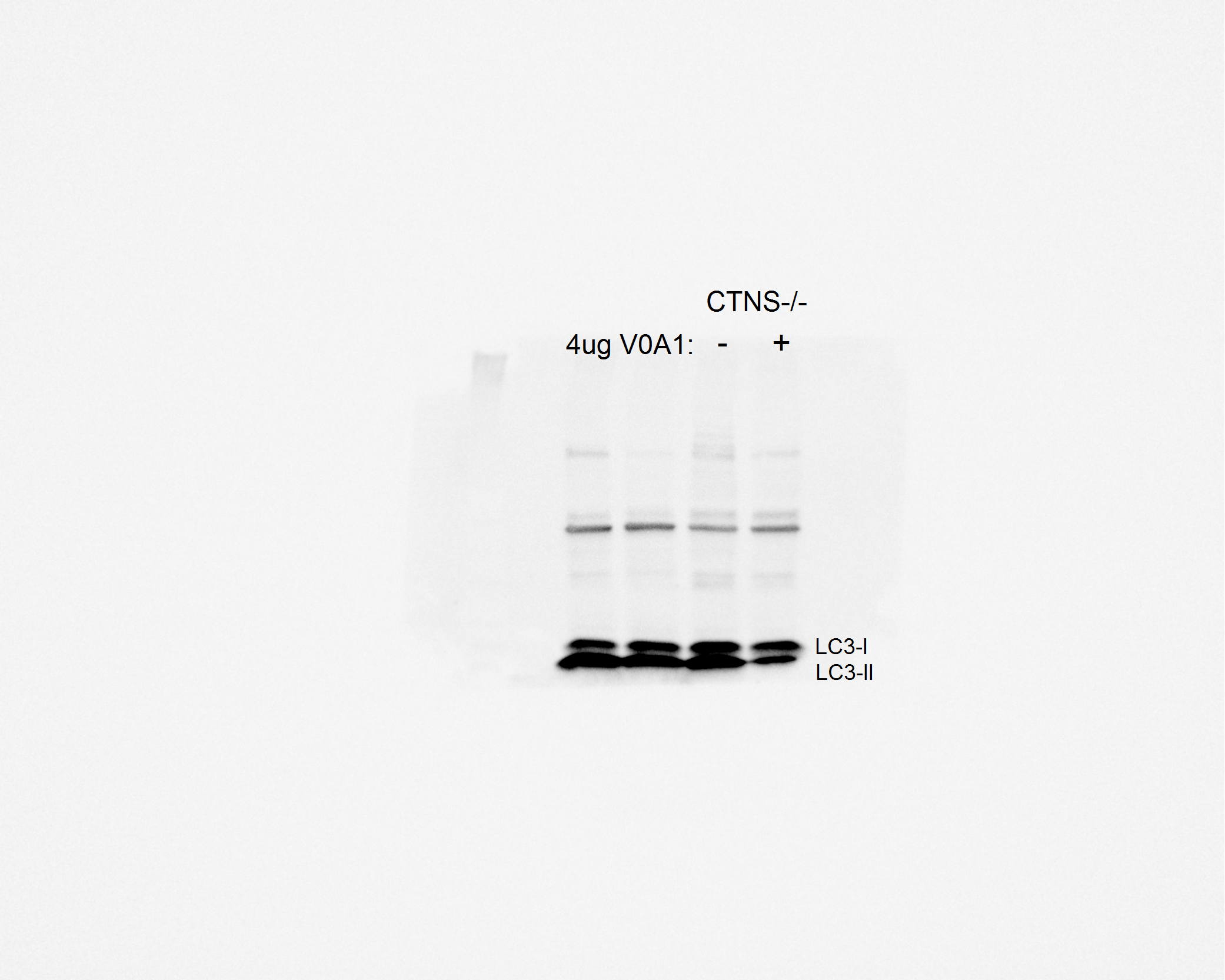

Supplement: Figure 7—source data 2. [file elife-94169-fig7-data2.zip › Figure 7-source data 2/Figure7A/LC3/7A - Gel 5_LC3.tif]

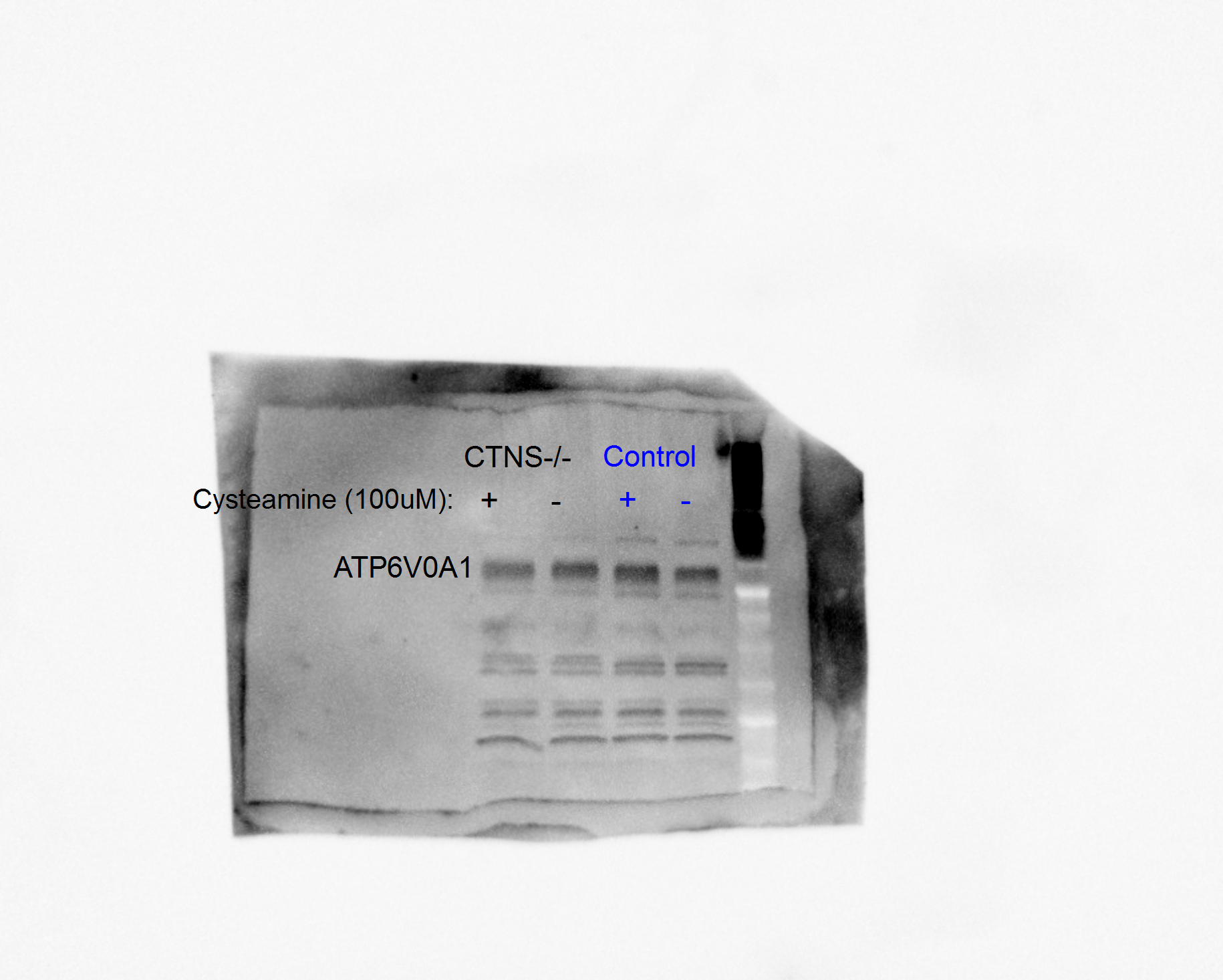

Supplement: Figure 8—source data 2. [file elife-94169-fig8-data2.zip › Figure 8-source data 2/Figure8A/8A - Gel 1_ATP6V0A1.tif]

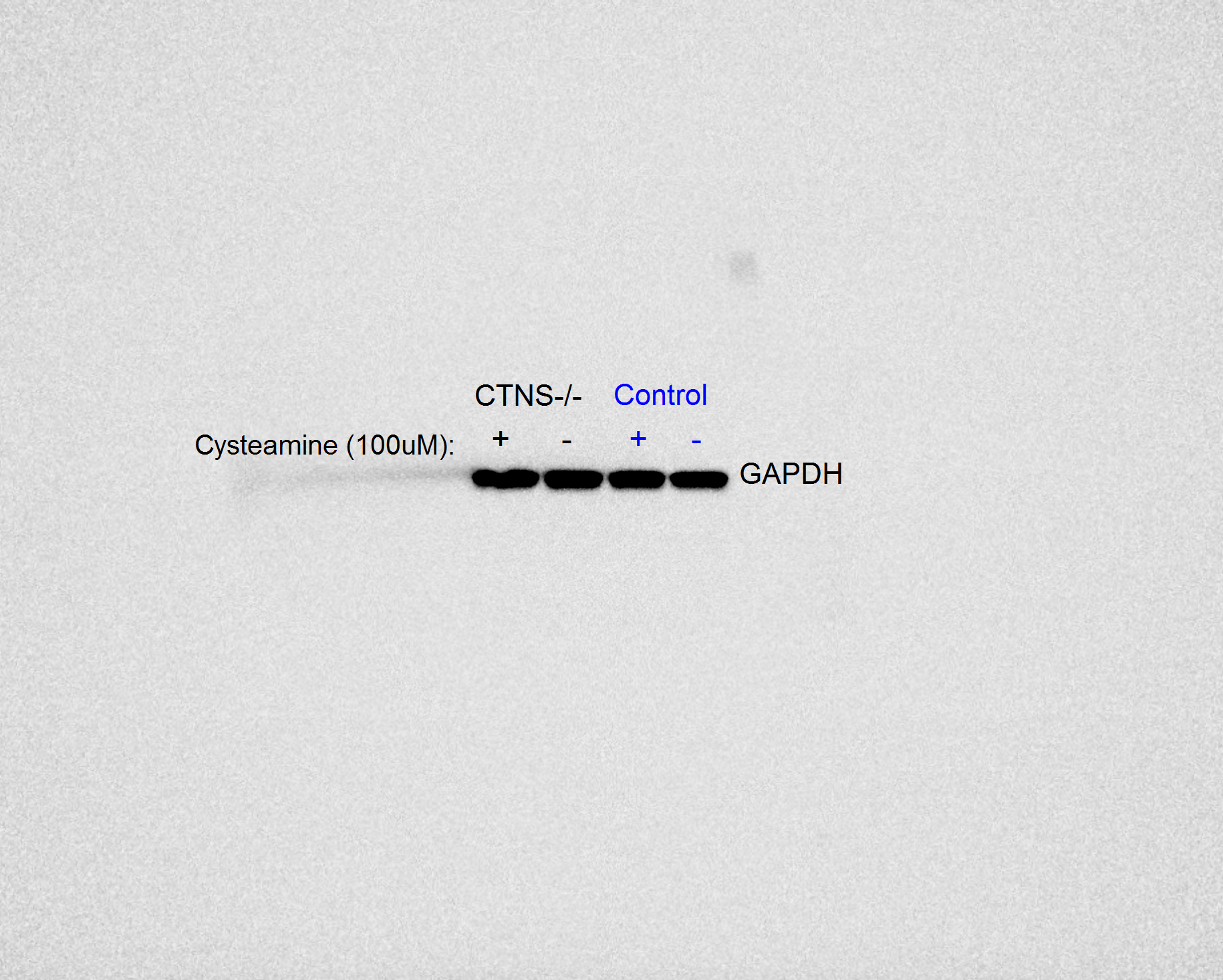

Supplement: Figure 8—source data 2. [file elife-94169-fig8-data2.zip › Figure 8-source data 2/Figure8A/8A - Gel 1_GAPDH.tif]

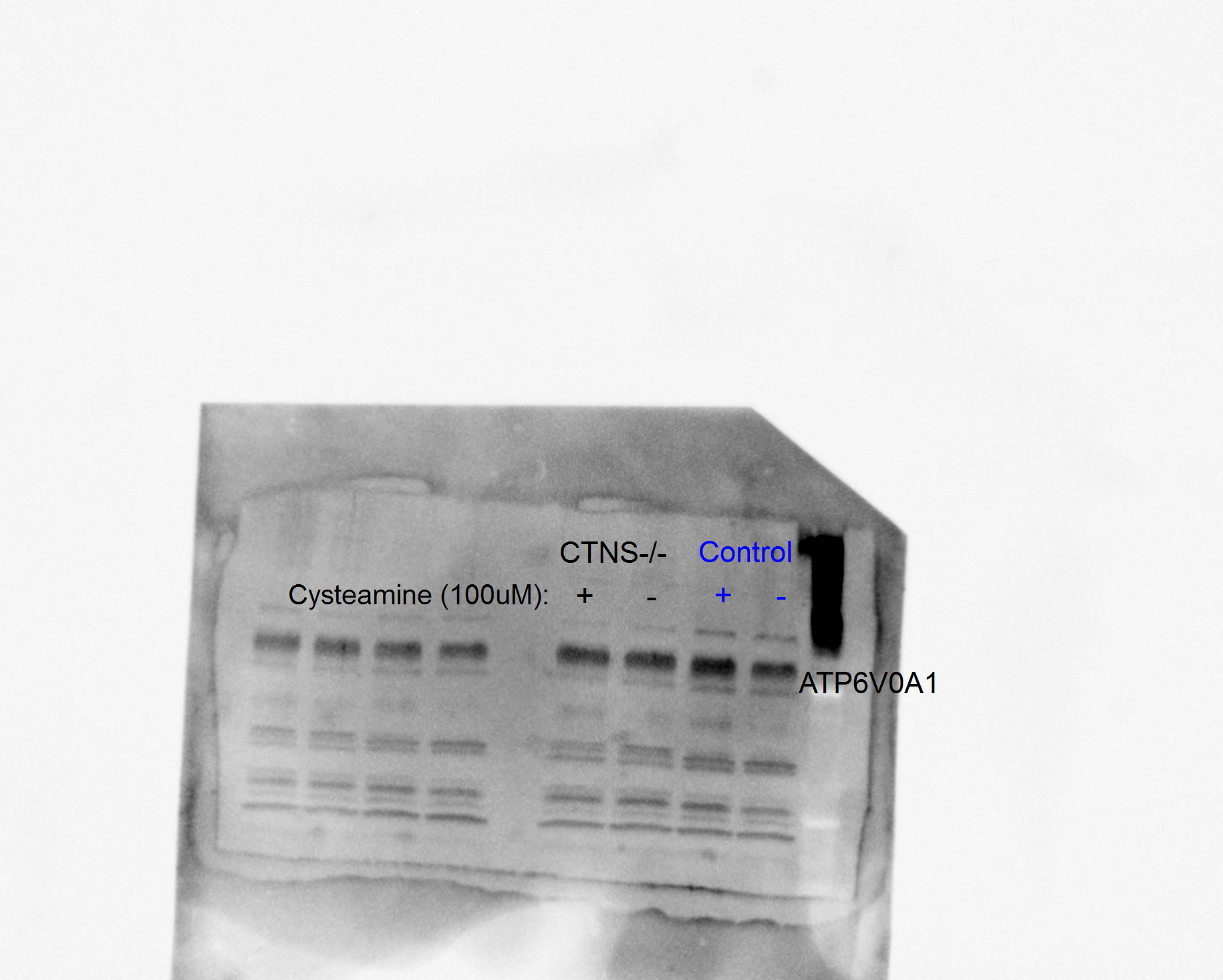

Supplement: Figure 8—source data 2. [file elife-94169-fig8-data2.zip › Figure 8-source data 2/Figure8A/8A - Gel 2_ATP6V0A1.tif]

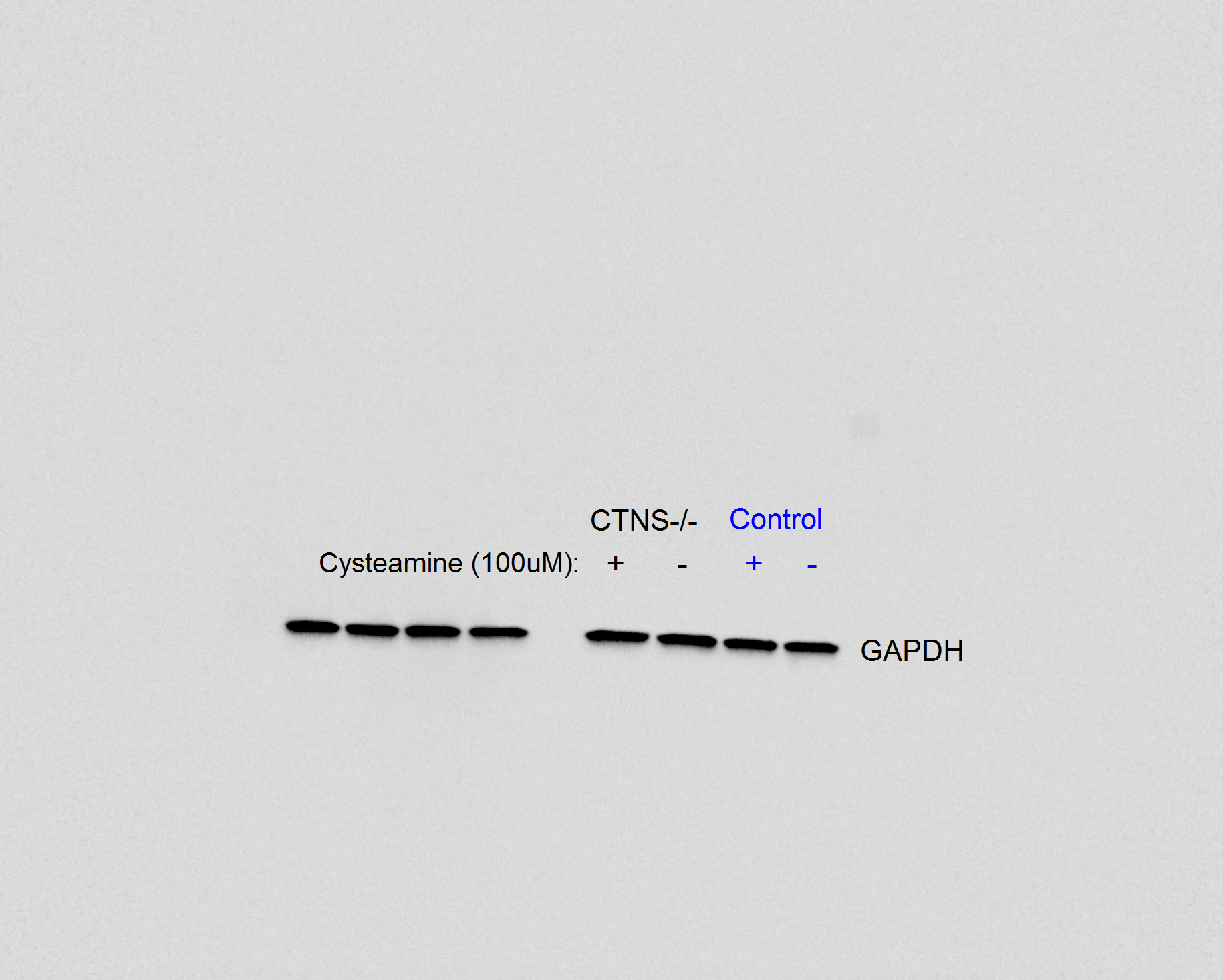

Supplement: Figure 8—source data 2. [file elife-94169-fig8-data2.zip › Figure 8-source data 2/Figure8A/8A - Gel 2_GAPDH.tif]

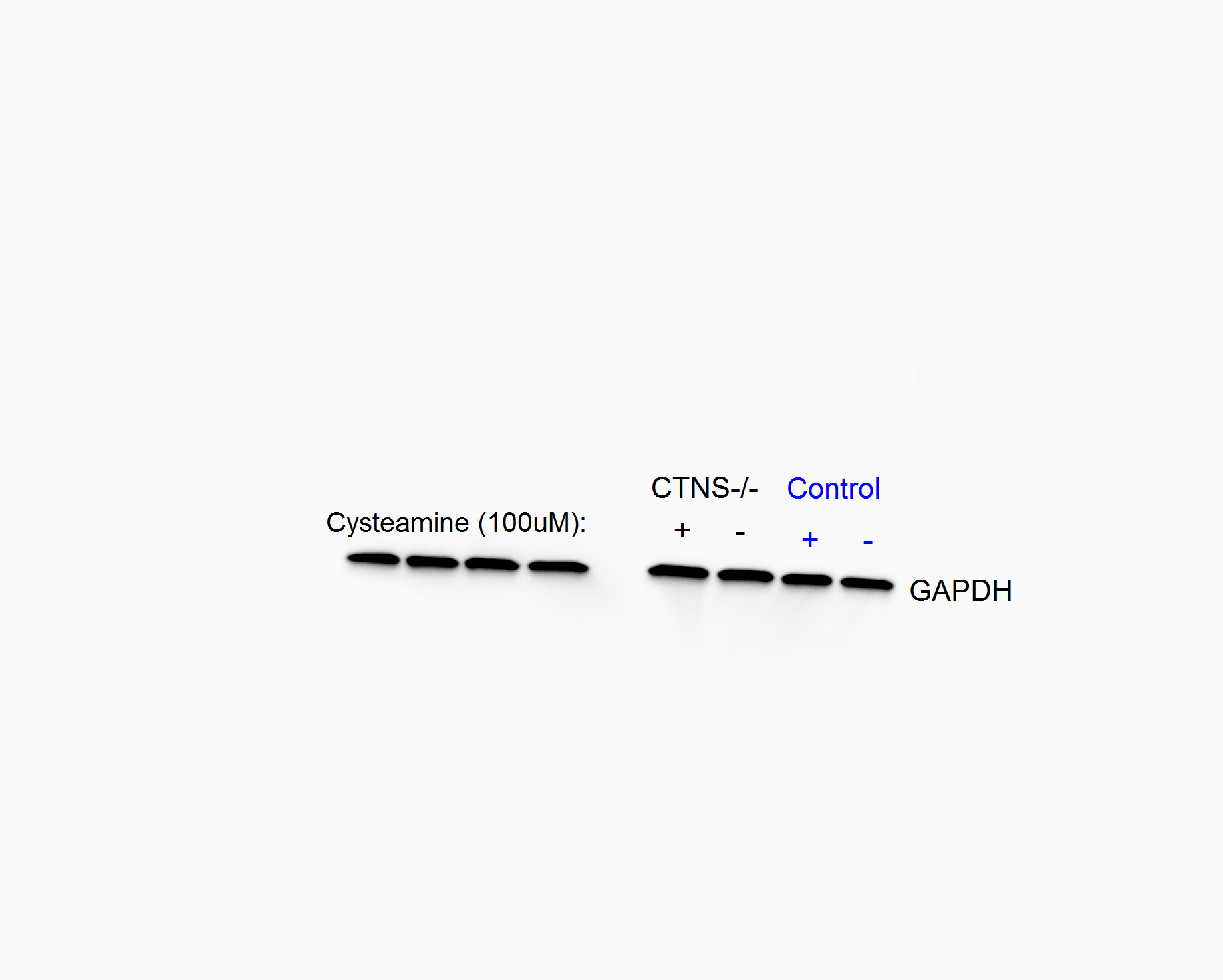

Supplement: Figure 8—source data 2. [file elife-94169-fig8-data2.zip › Figure 8-source data 2/Figure8A/8A - Gel 3_GAPDH.tif]

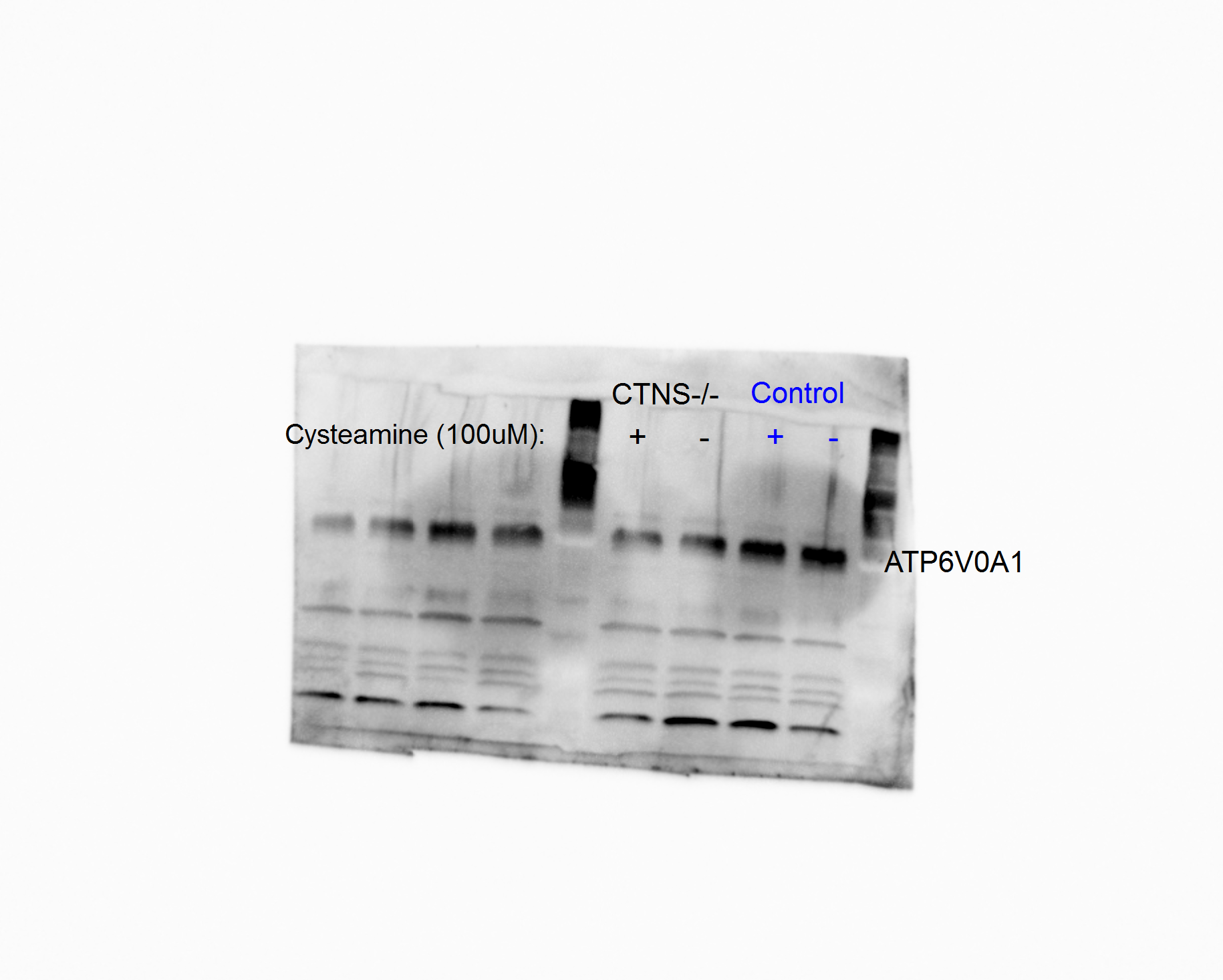

Supplement: Figure 8—source data 2. [file elife-94169-fig8-data2.zip › Figure 8-source data 2/Figure8A/8A Gel 3_ATP6V0A1.tif]

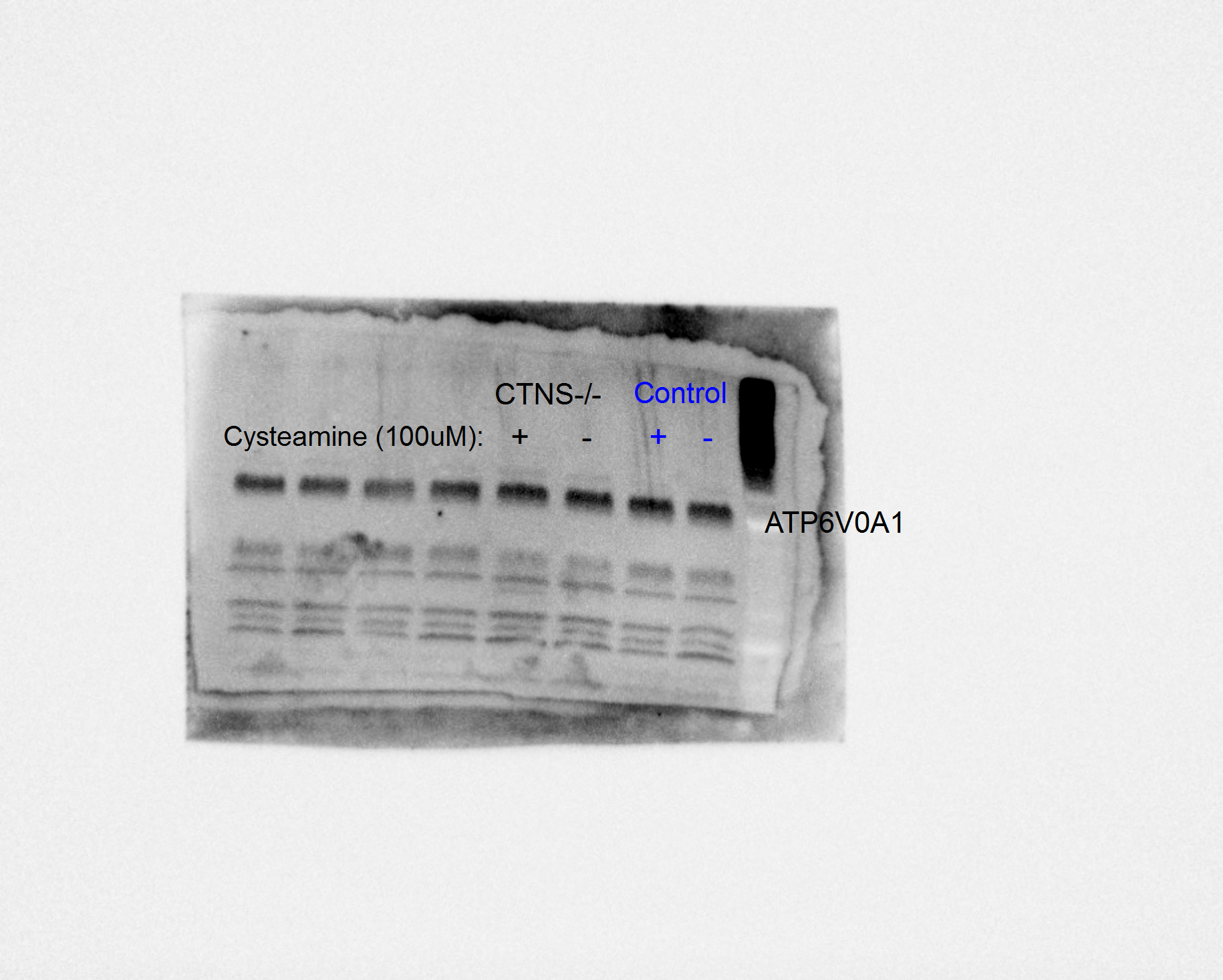

Supplement: Figure 8—source data 2. [file elife-94169-fig8-data2.zip › Figure 8-source data 2/Figure8A/8A Gel 4_ATP6V0A1.tif]

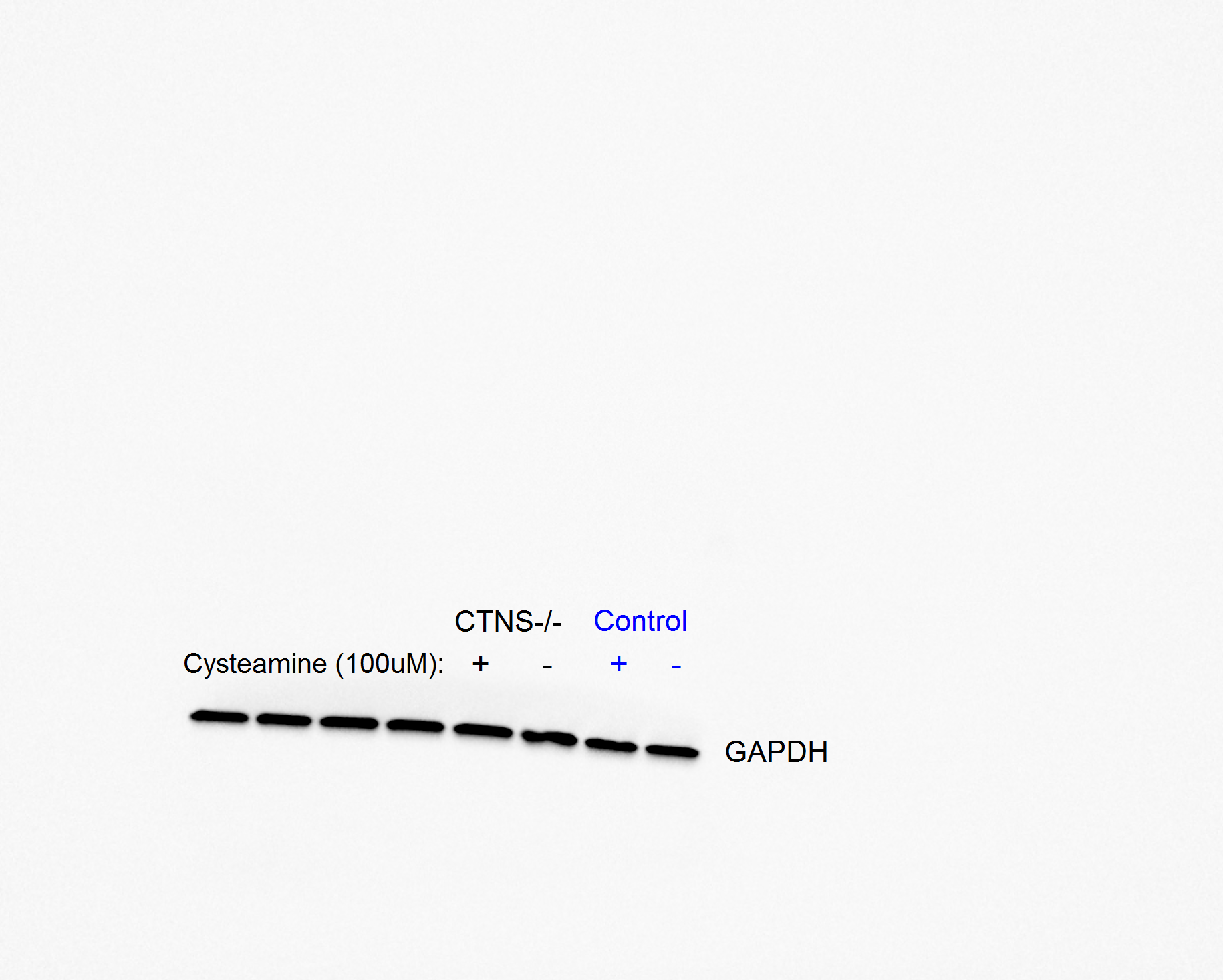

Supplement: Figure 8—source data 2. [file elife-94169-fig8-data2.zip › Figure 8-source data 2/Figure8A/8A Gel 4_GAPDH.tif]

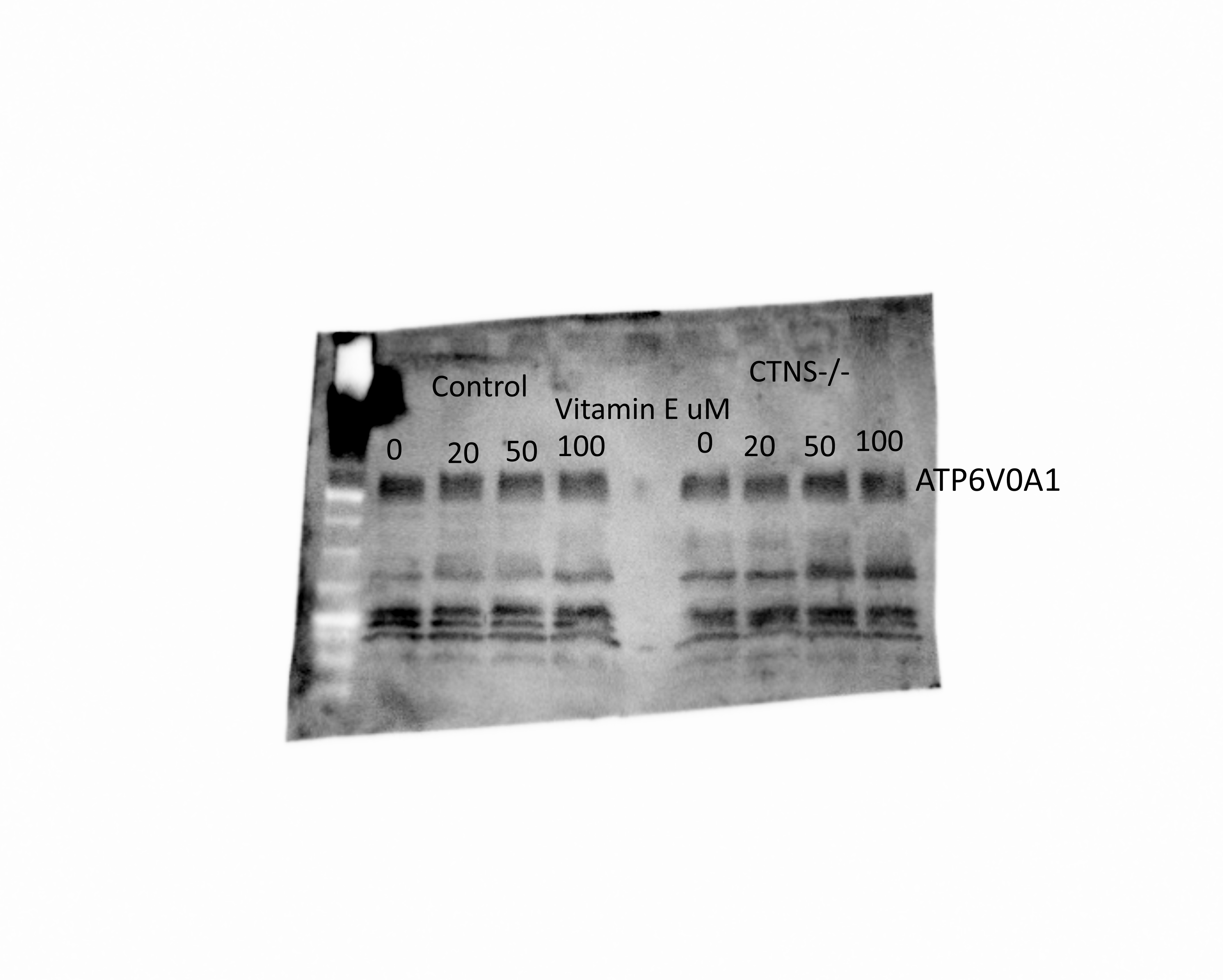

Supplement: Figure 8—source data 2. [file elife-94169-fig8-data2.zip › Figure 8-source data 2/Figure8B/8B Gel 1_ATP6V0A1.tif]

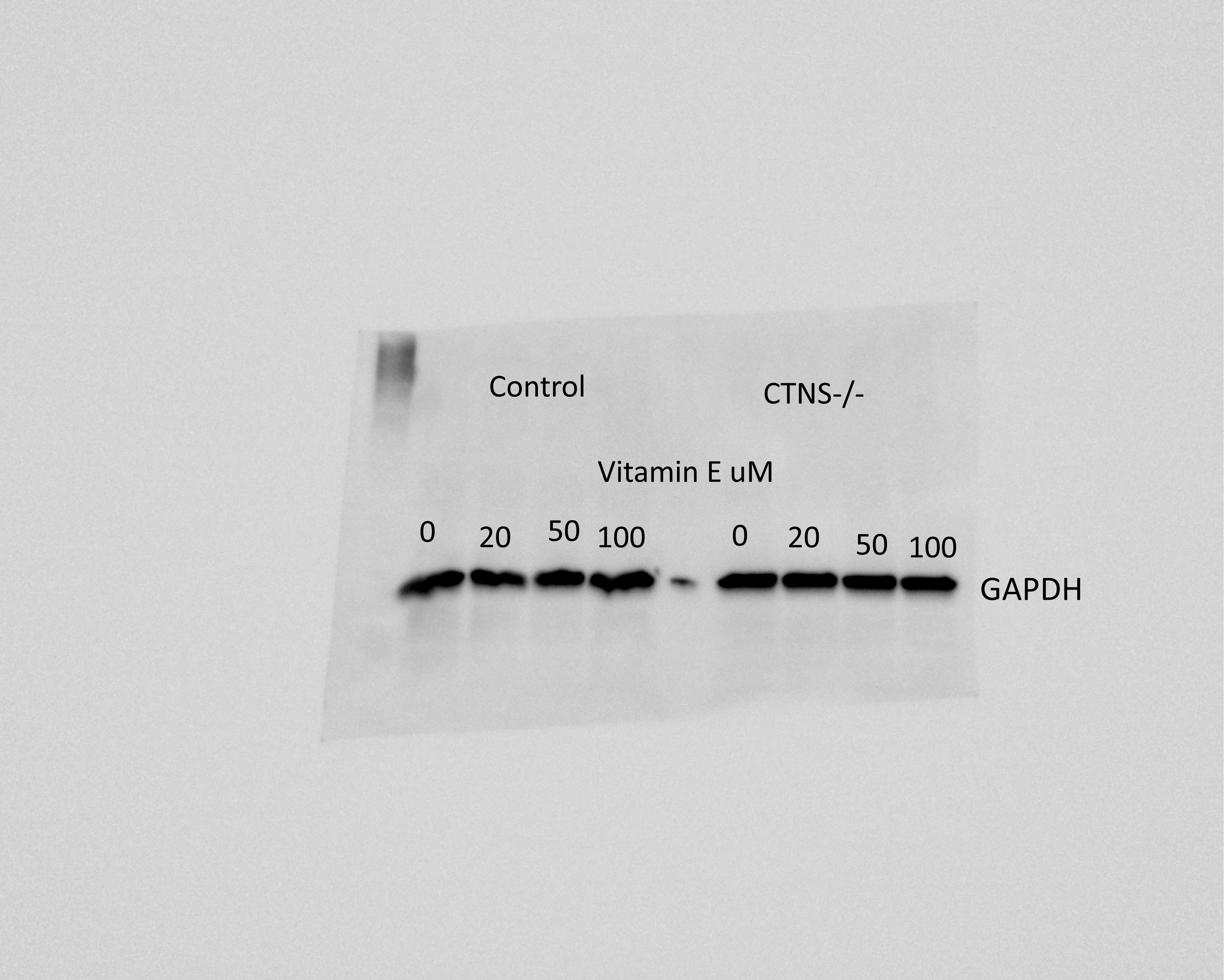

Supplement: Figure 8—source data 2. [file elife-94169-fig8-data2.zip › Figure 8-source data 2/Figure8B/8B Gel 1_GAPDH.tif]

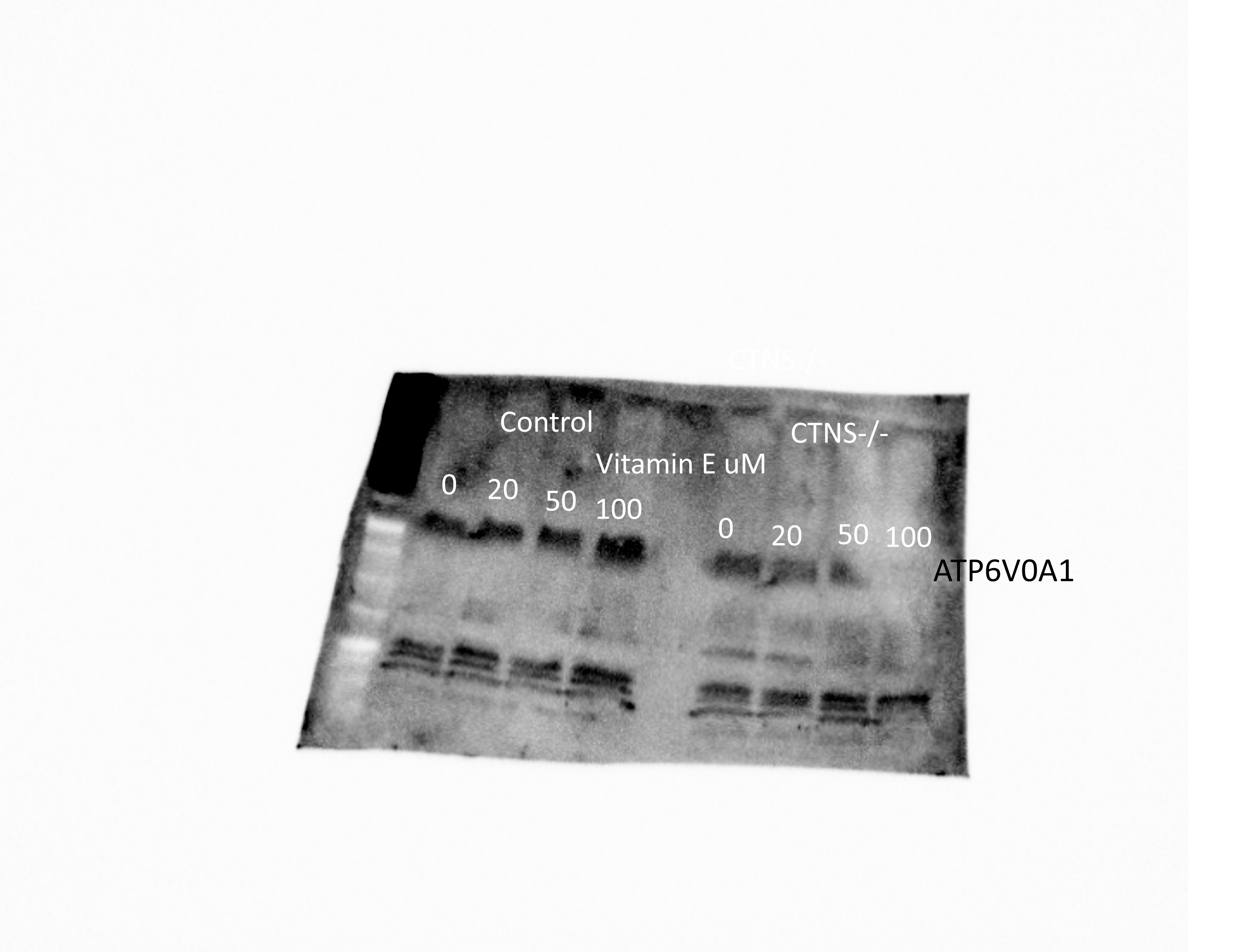

Supplement: Figure 8—source data 2. [file elife-94169-fig8-data2.zip › Figure 8-source data 2/Figure8B/8B Gel 2_ATP6V0A1.tif]

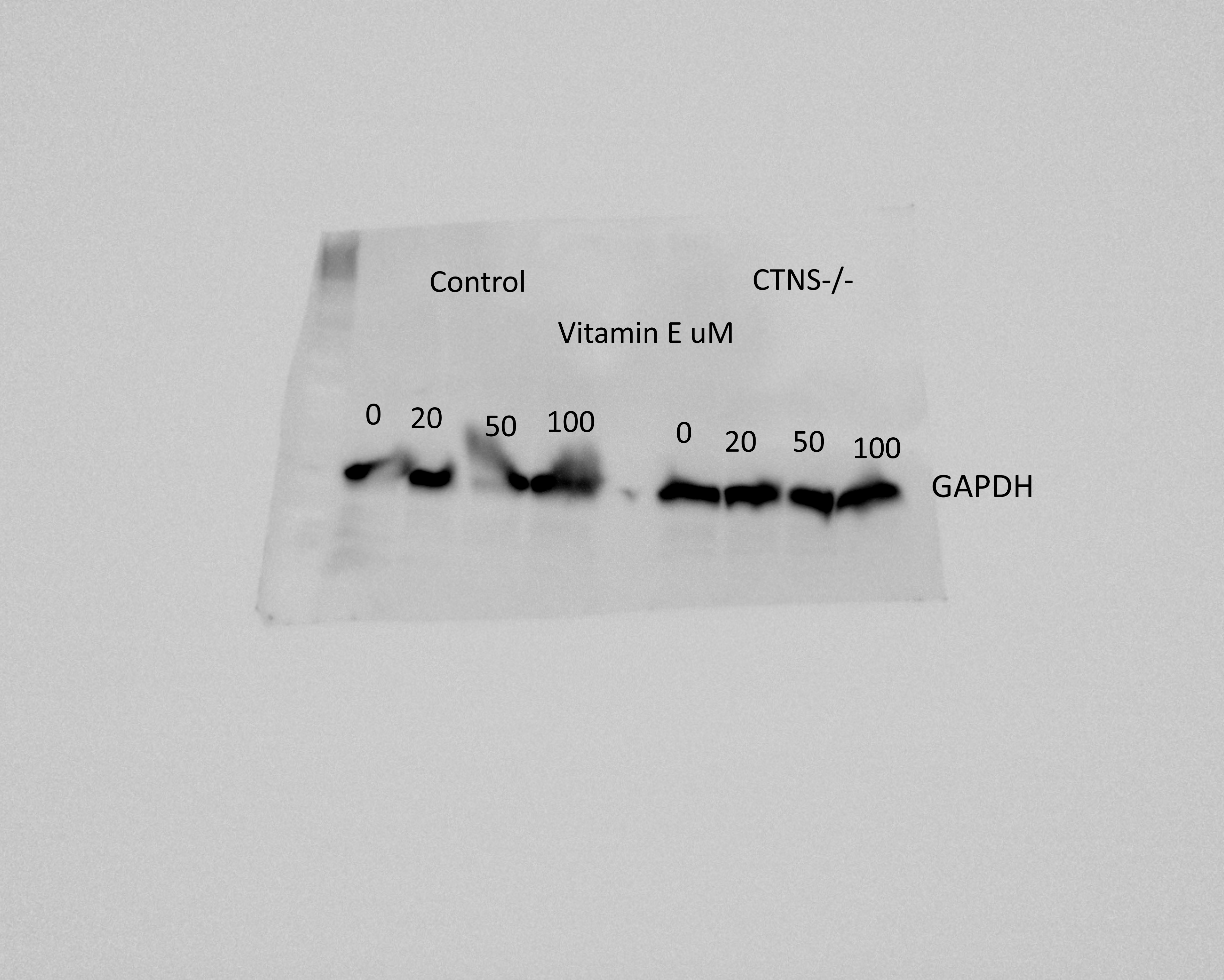

Supplement: Figure 8—source data 2. [file elife-94169-fig8-data2.zip › Figure 8-source data 2/Figure8B/8B Gel 2_GAPDH.tif]

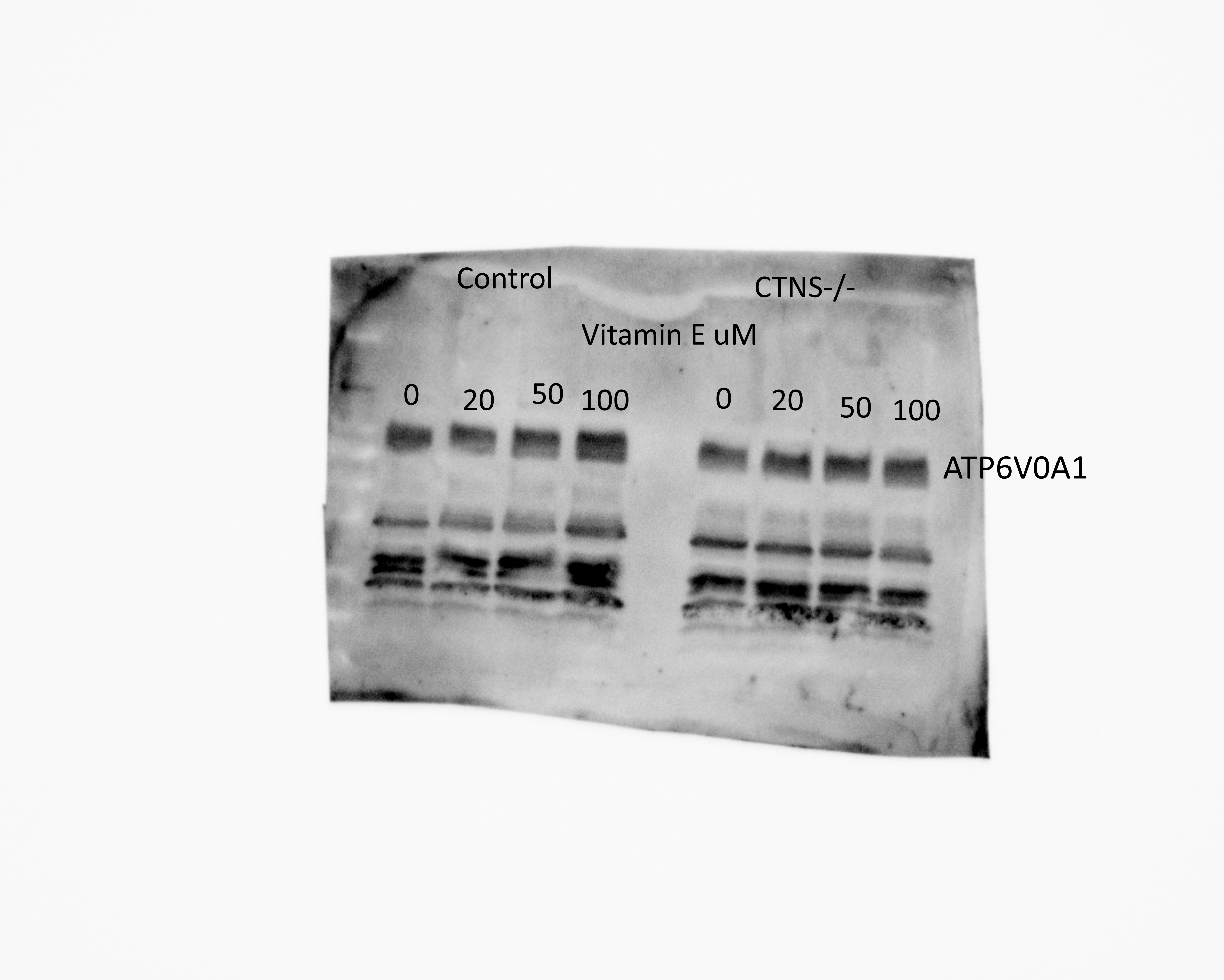

Supplement: Figure 8—source data 2. [file elife-94169-fig8-data2.zip › Figure 8-source data 2/Figure8B/8B Gel 3_ATP6V0A1.tif]

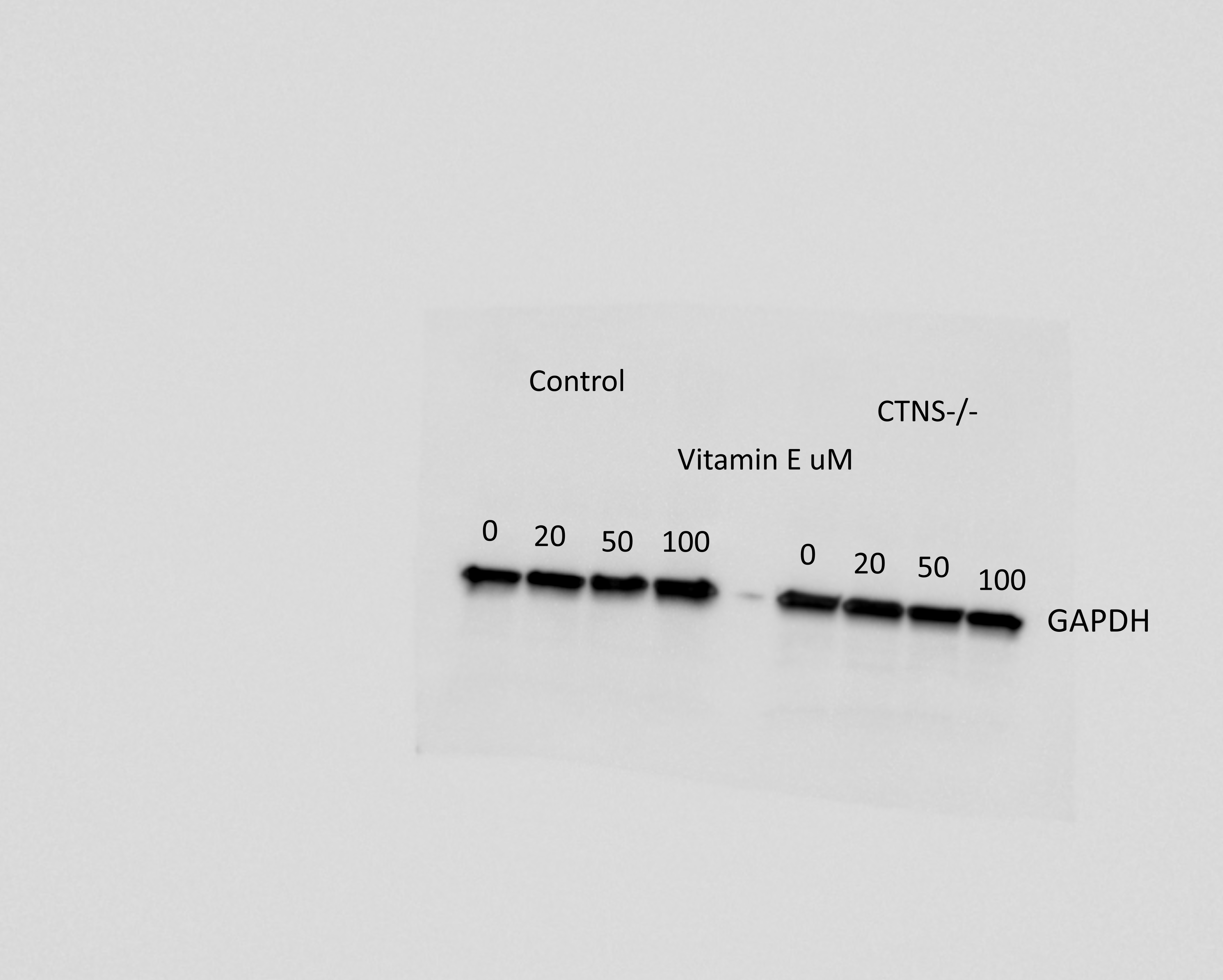

Supplement: Figure 8—source data 2. [file elife-94169-fig8-data2.zip › Figure 8-source data 2/Figure8B/8B Gel 3_GAPDH.tif]

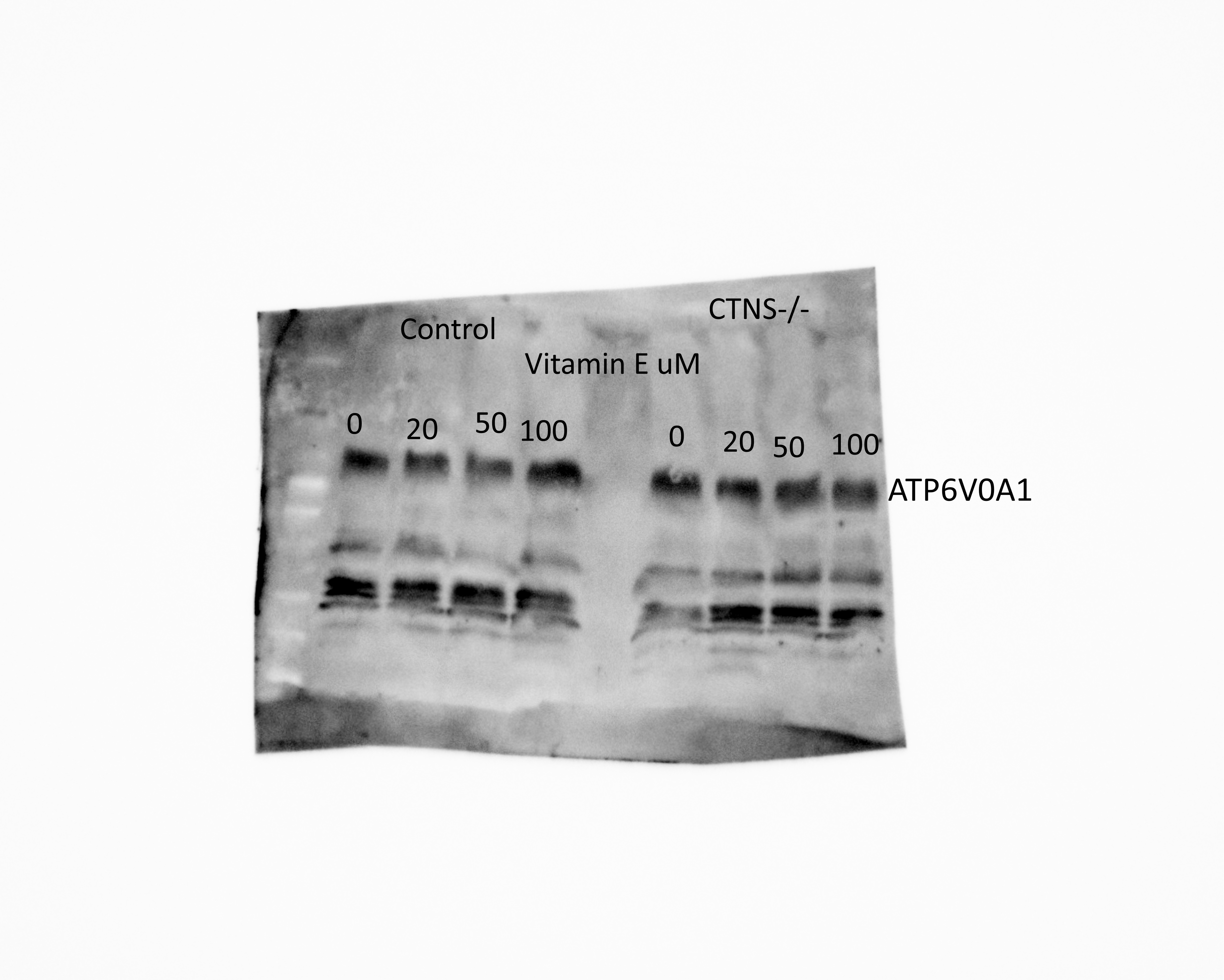

Supplement: Figure 8—source data 2. [file elife-94169-fig8-data2.zip › Figure 8-source data 2/Figure8B/8B Gel 4_ATP6V0A1.tif]

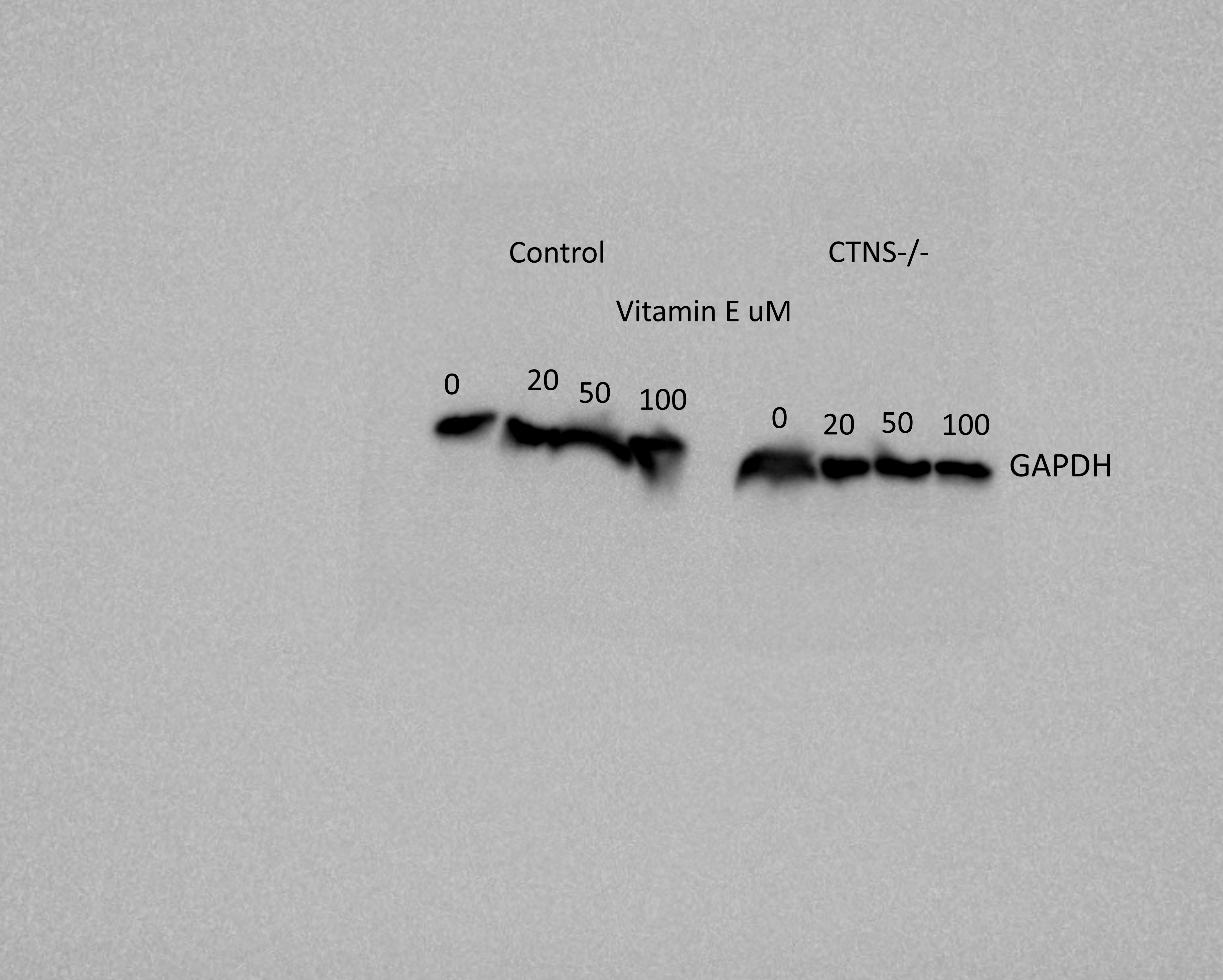

Supplement: Figure 8—source data 2. [file elife-94169-fig8-data2.zip › Figure 8-source data 2/Figure8B/8B Gel 4_GAPDH.tif]

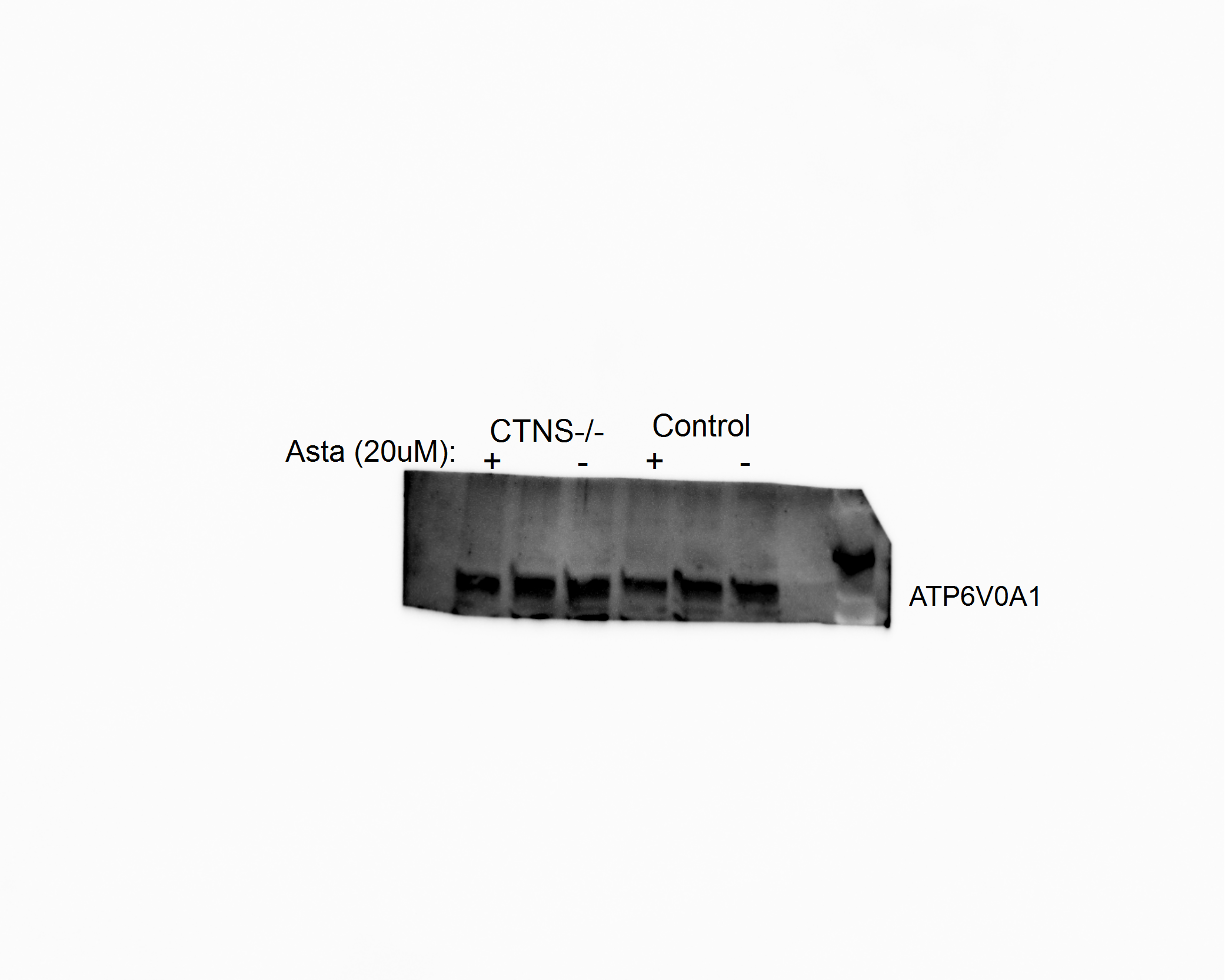

Supplement: Figure 8—source data 2. [file elife-94169-fig8-data2.zip › Figure 8-source data 2/Figure8C/ATP6V0A1/8C - Gel 1_ATP6V0A1.tif]

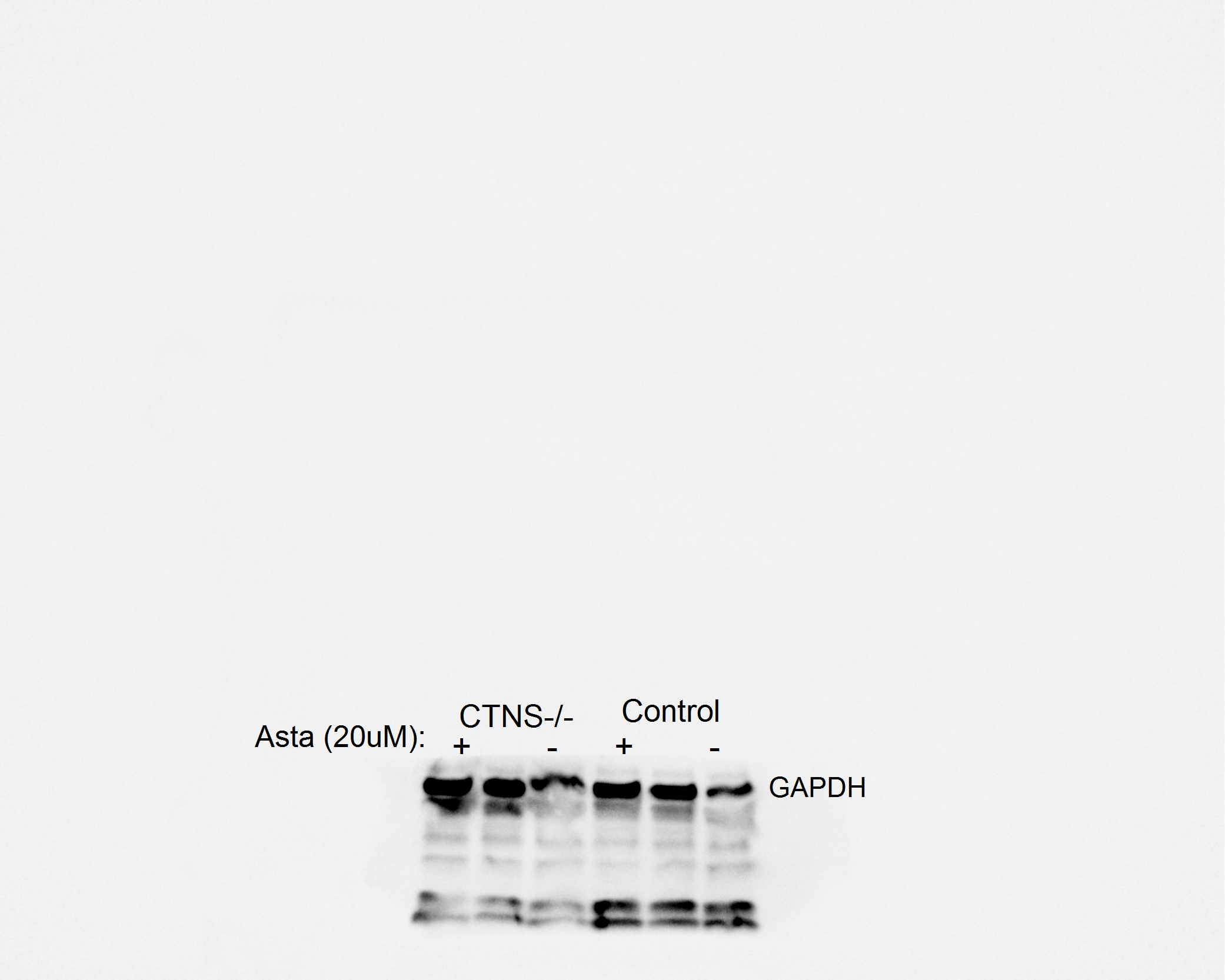

Supplement: Figure 8—source data 2. [file elife-94169-fig8-data2.zip › Figure 8-source data 2/Figure8C/ATP6V0A1/8C - Gel 1_GAPDH.tif]

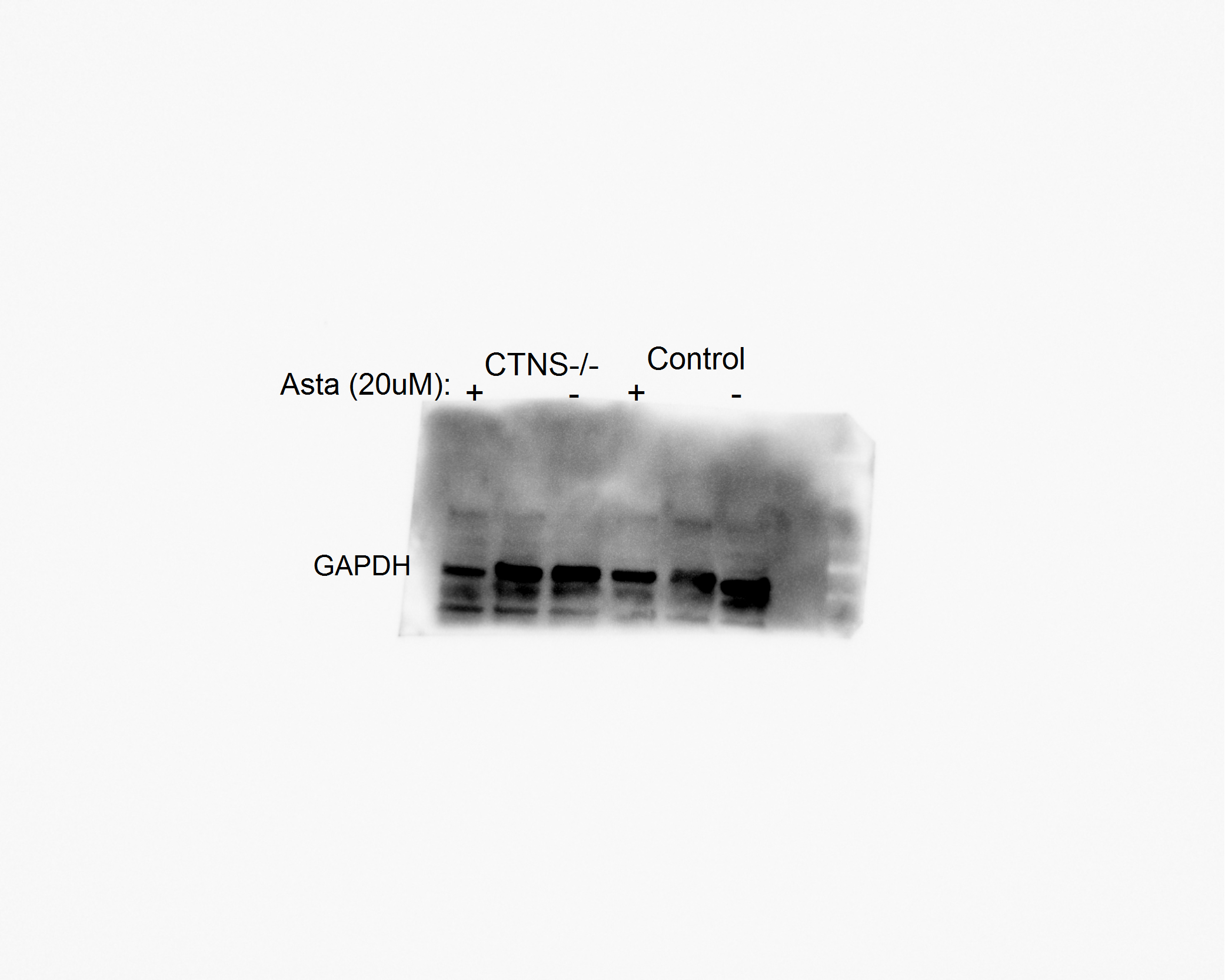

Supplement: Figure 8—source data 2. [file elife-94169-fig8-data2.zip › Figure 8-source data 2/Figure8C/ATP6V0A1/8C - Gel 2_GAPDH.tif]

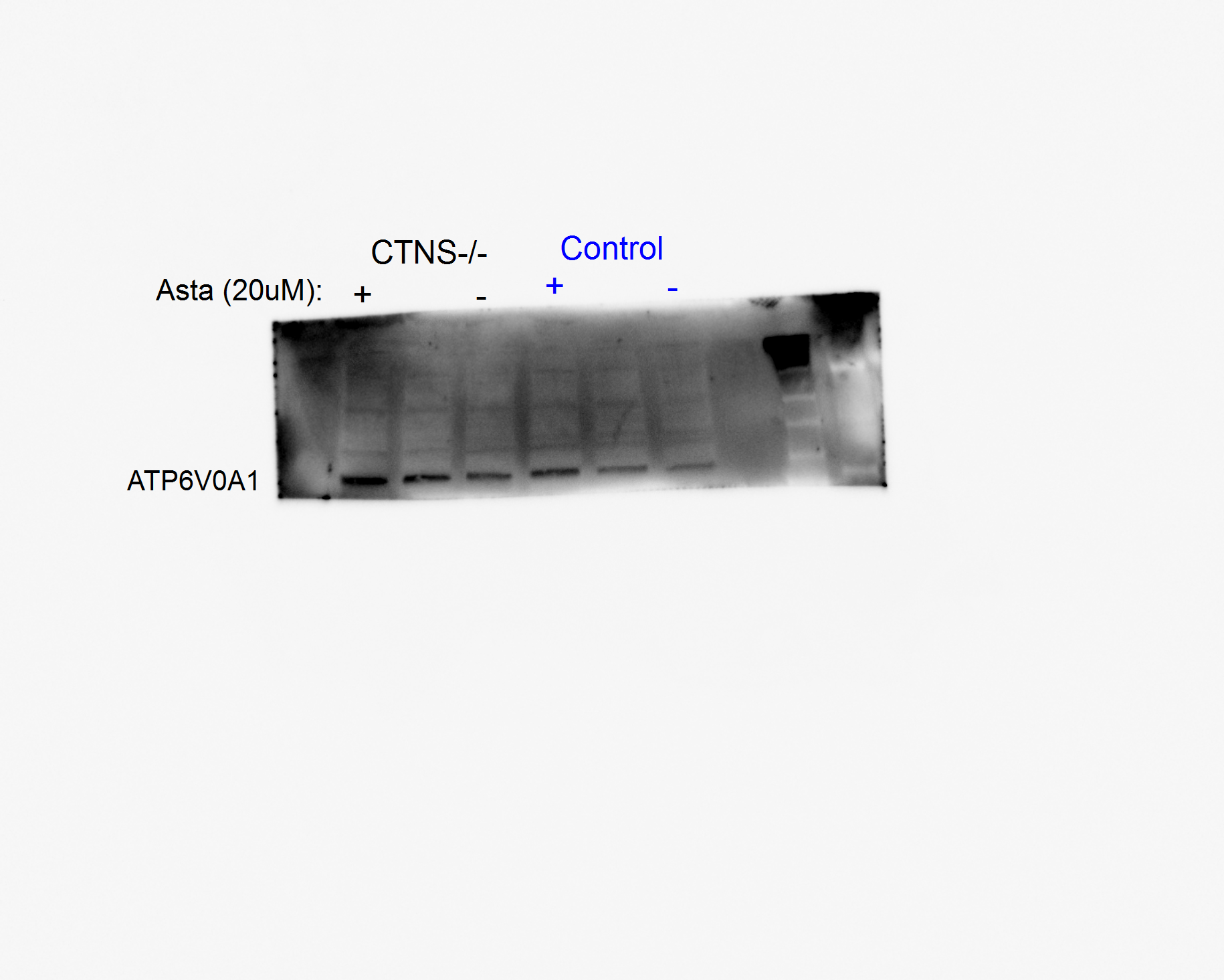

Supplement: Figure 8—source data 2. [file elife-94169-fig8-data2.zip › Figure 8-source data 2/Figure8C/ATP6V0A1/8C - Gel 3_ATP6V0A1.tif]

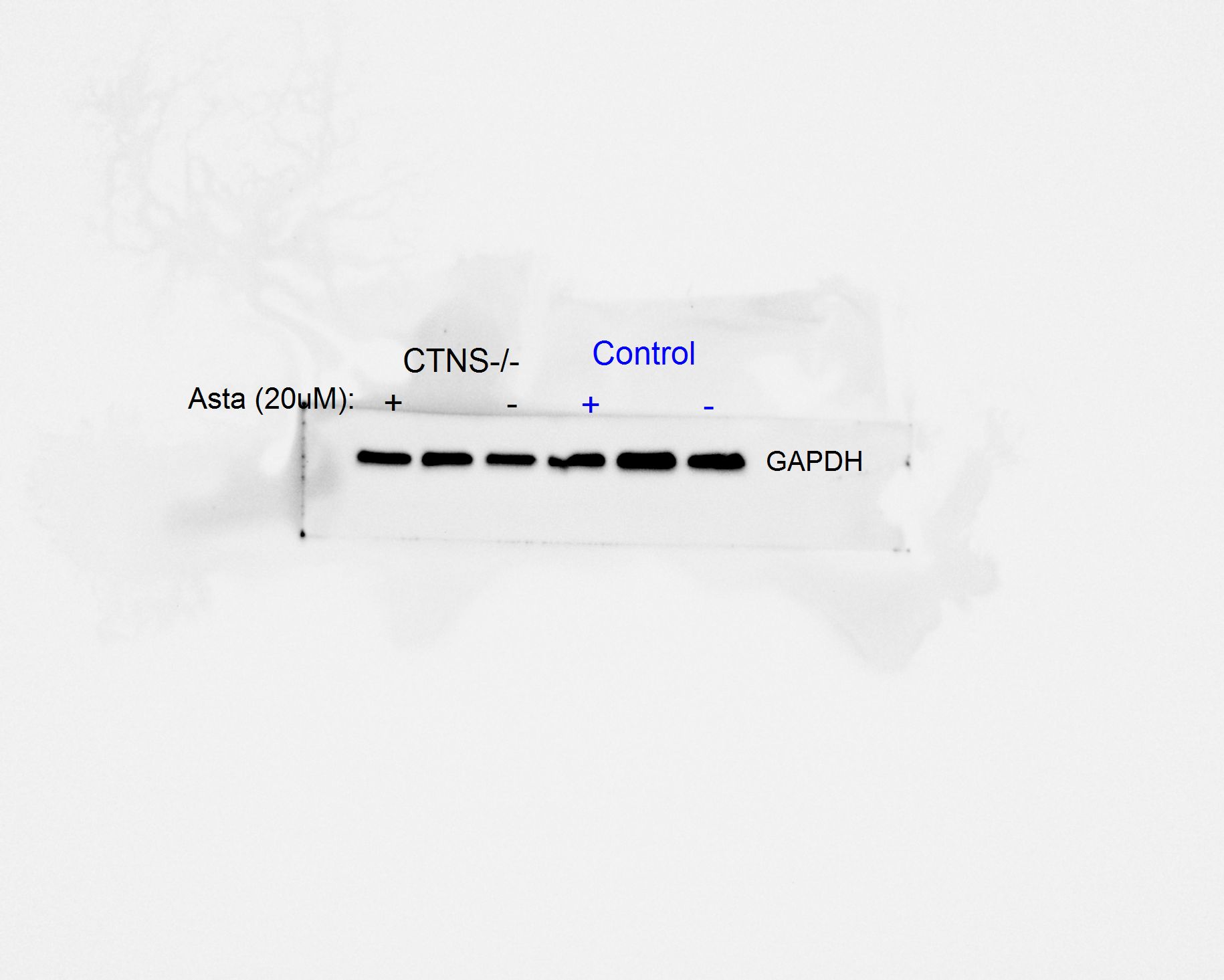

Supplement: Figure 8—source data 2. [file elife-94169-fig8-data2.zip › Figure 8-source data 2/Figure8C/ATP6V0A1/8C - Gel 3_GAPDH.tif]

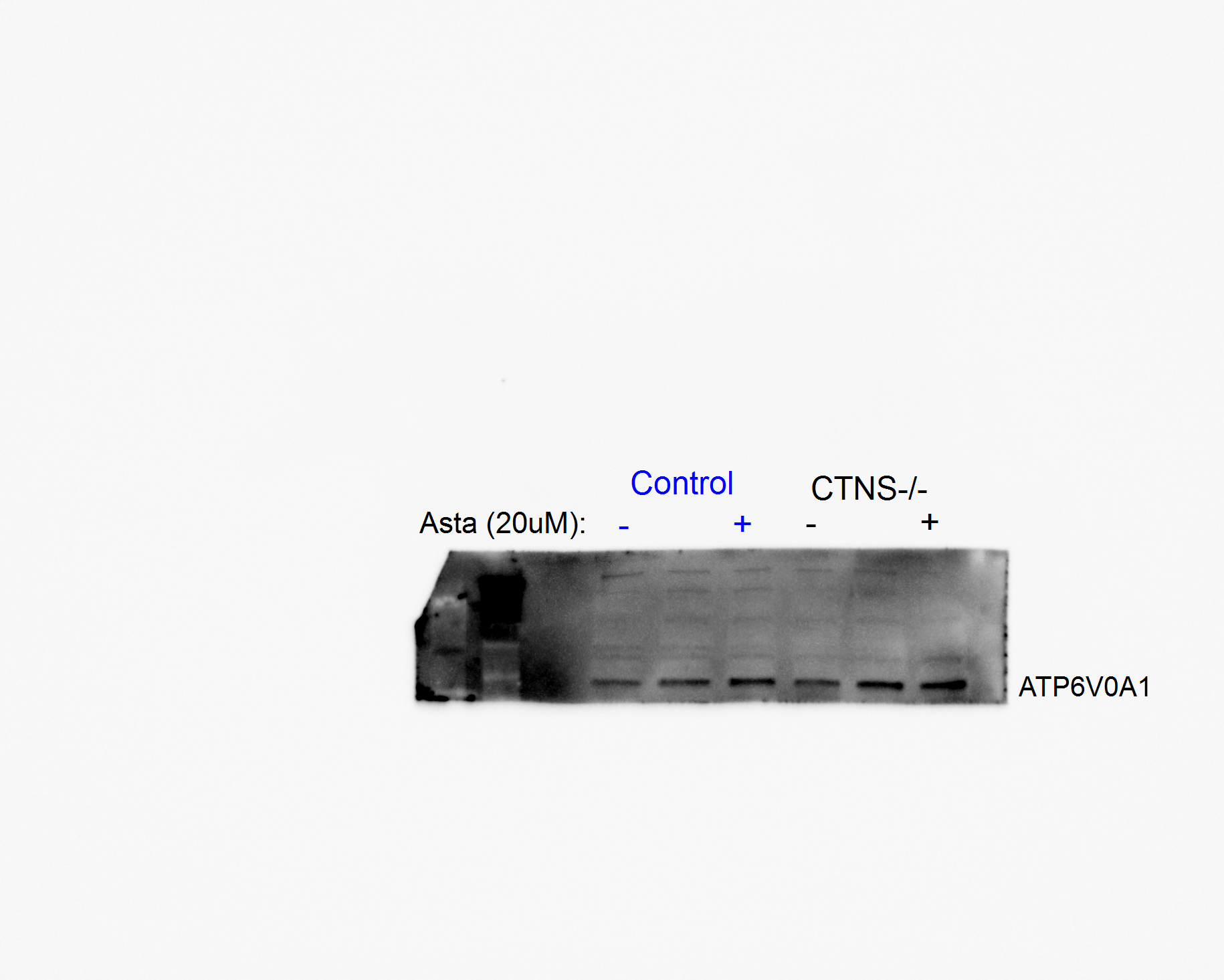

Supplement: Figure 8—source data 2. [file elife-94169-fig8-data2.zip › Figure 8-source data 2/Figure8C/ATP6V0A1/8C - Gel 4_ATP6V0A1.tif]

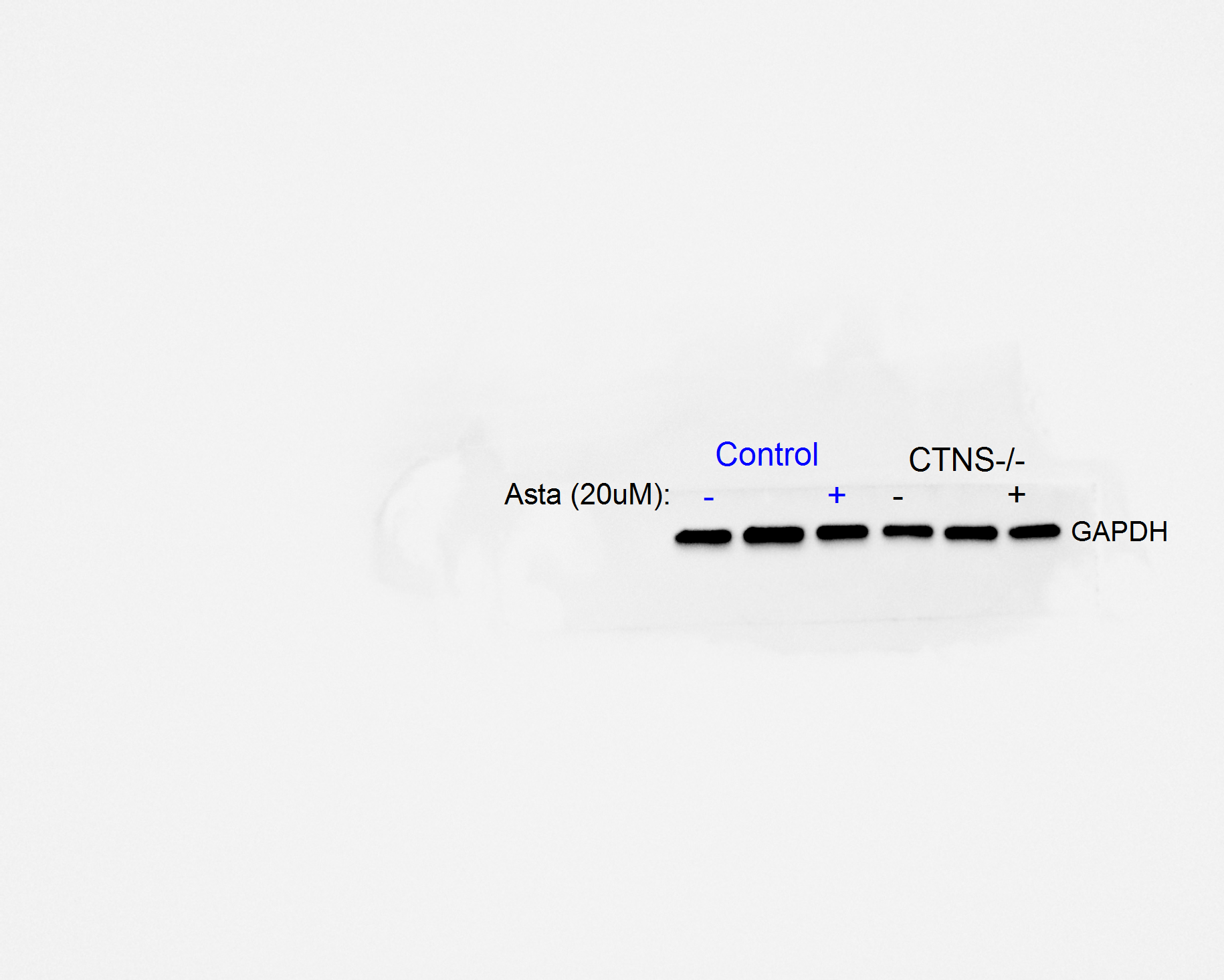

Supplement: Figure 8—source data 2. [file elife-94169-fig8-data2.zip › Figure 8-source data 2/Figure8C/ATP6V0A1/8C - Gel 4_GAPDH.tif]

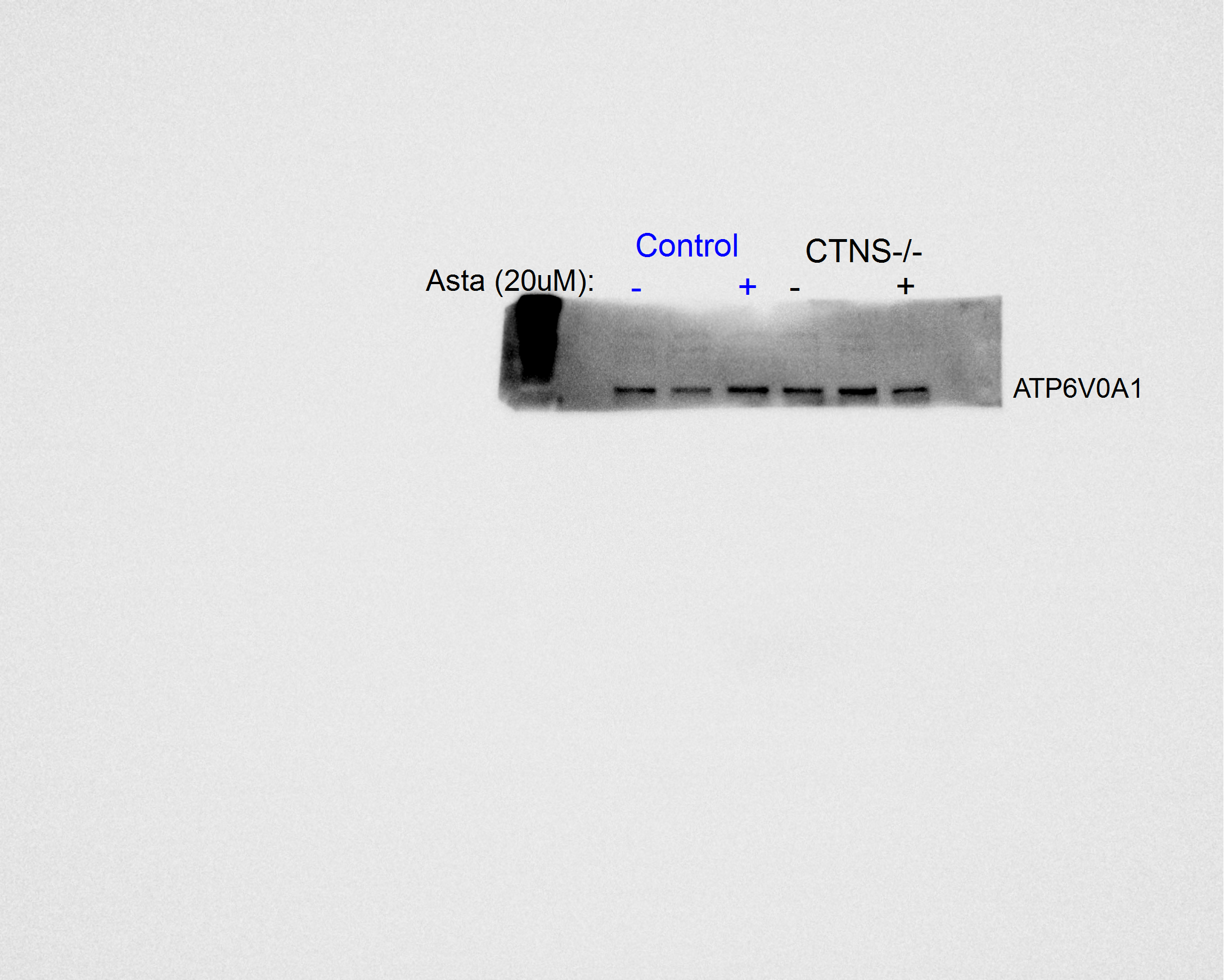

Supplement: Figure 8—source data 2. [file elife-94169-fig8-data2.zip › Figure 8-source data 2/Figure8C/ATP6V0A1/8C - Gel 5_ATP6V0A1.tif]

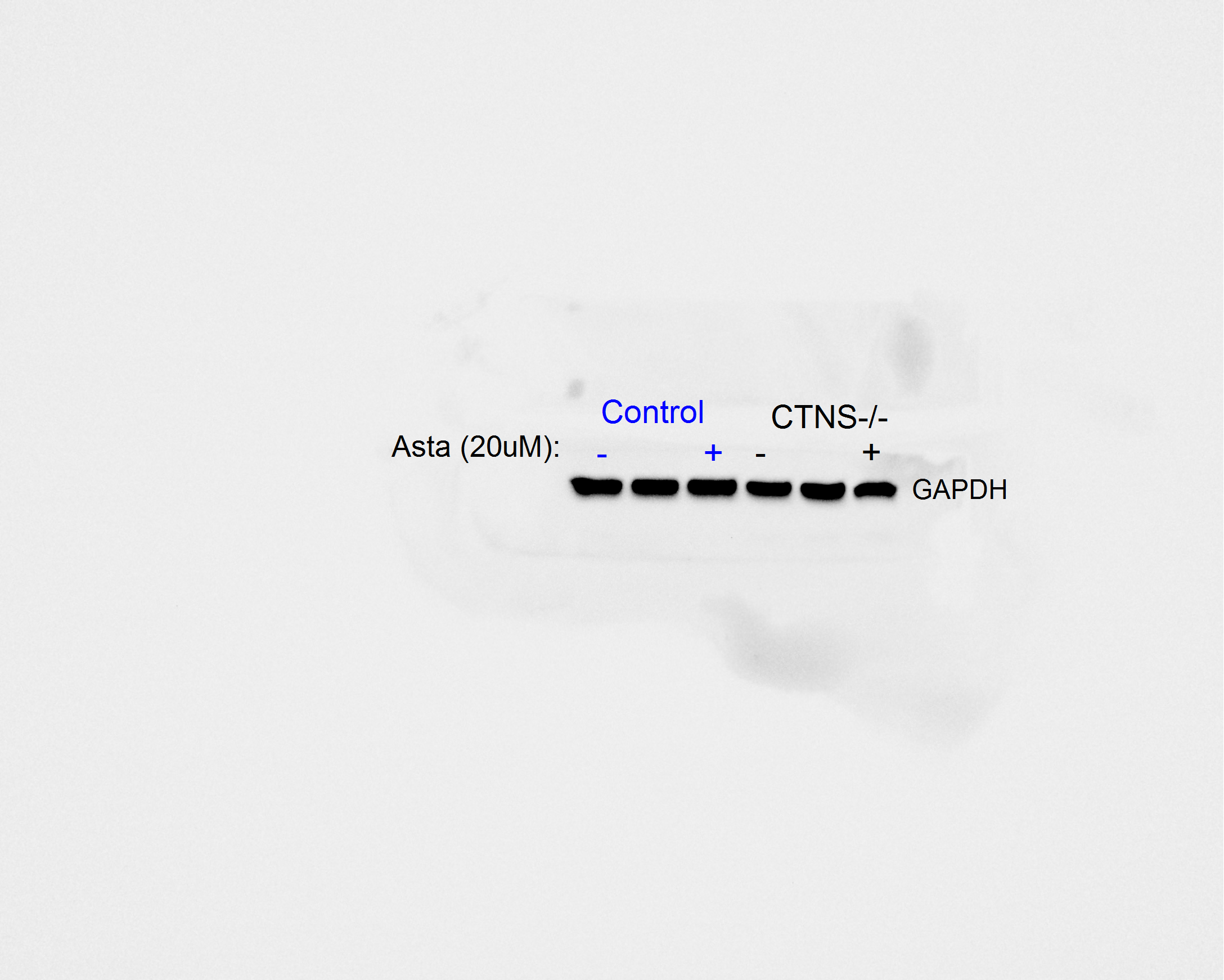

Supplement: Figure 8—source data 2. [file elife-94169-fig8-data2.zip › Figure 8-source data 2/Figure8C/ATP6V0A1/8C - Gel 5_GAPDH.tif]

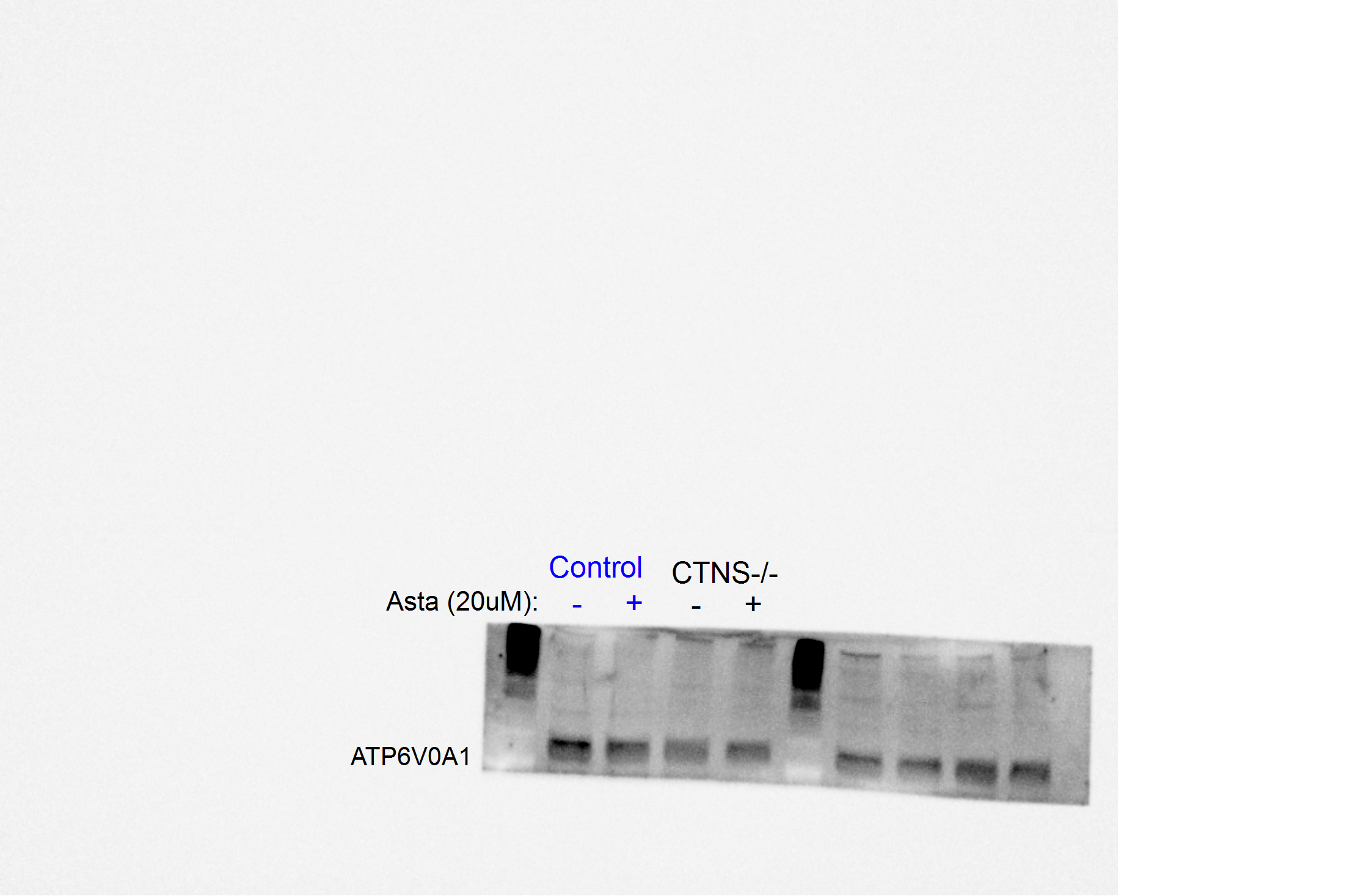

Supplement: Figure 8—source data 2. [file elife-94169-fig8-data2.zip › Figure 8-source data 2/Figure8C/ATP6V0A1/8C - Gel 6_ATP6V0A1.tif]

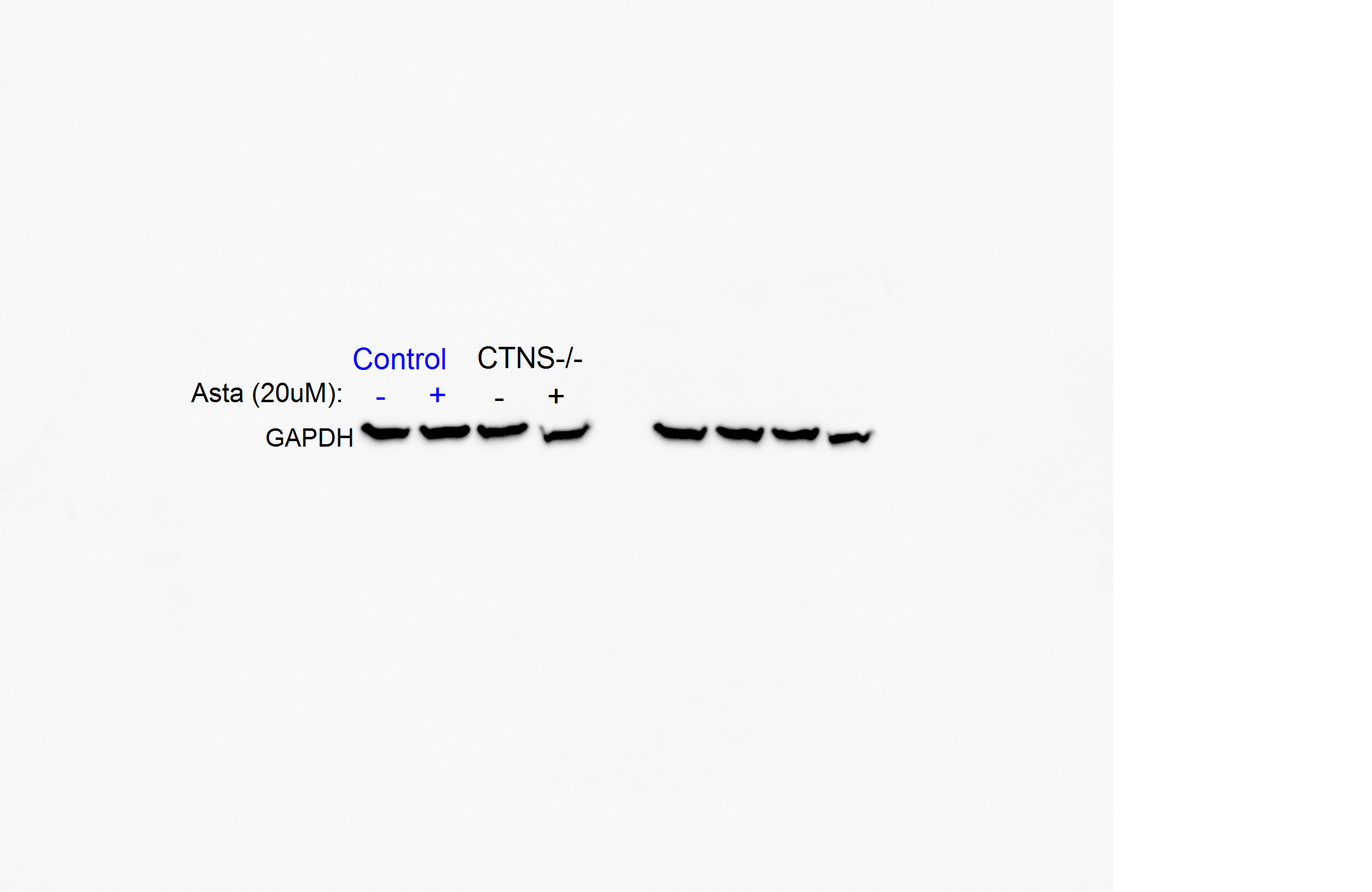

Supplement: Figure 8—source data 2. [file elife-94169-fig8-data2.zip › Figure 8-source data 2/Figure8C/ATP6V0A1/8C - Gel 6_GAPDH.tif]

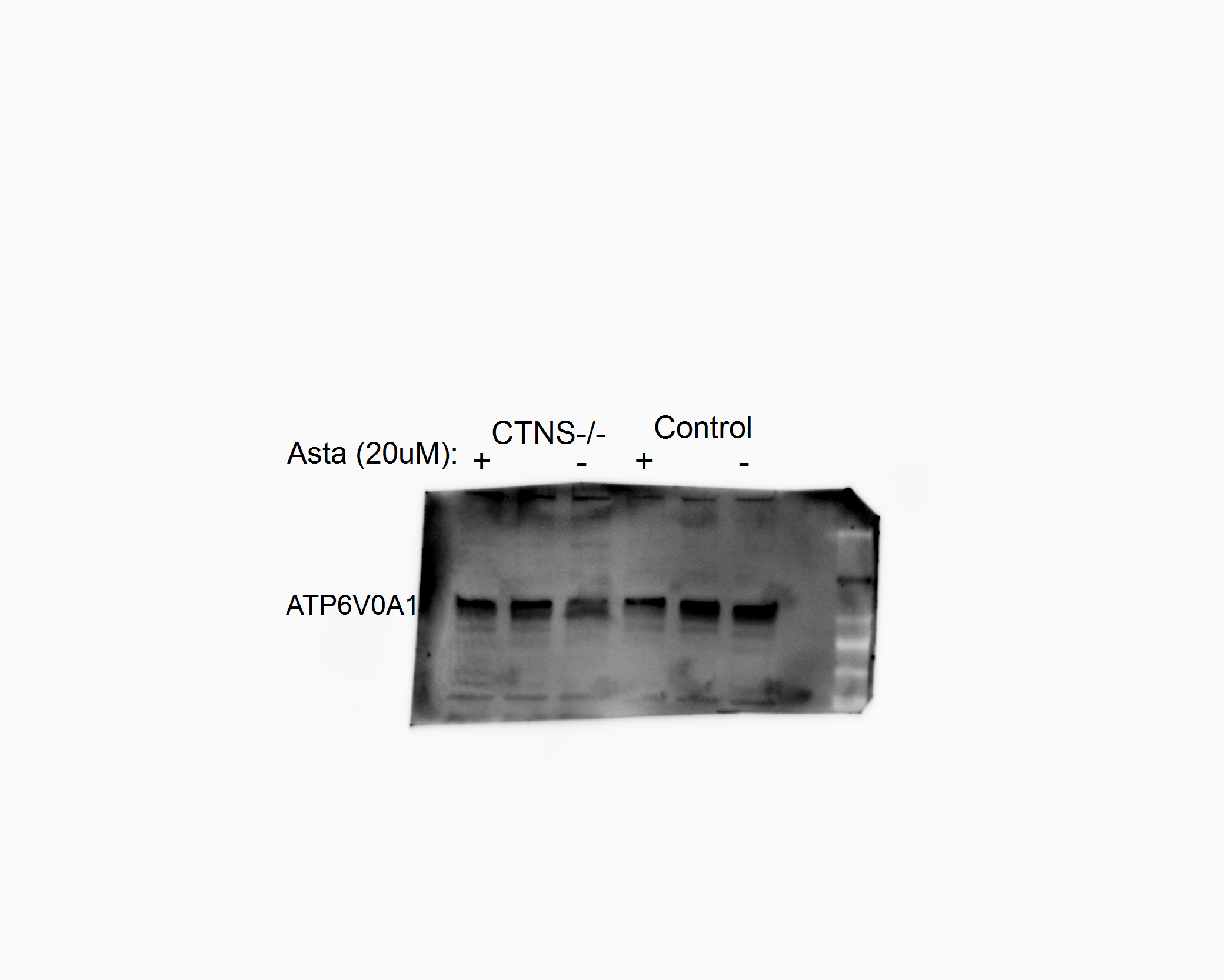

Supplement: Figure 8—source data 2. [file elife-94169-fig8-data2.zip › Figure 8-source data 2/Figure8C/ATP6V0A1/8C- Gel 2_ATP6V0A1.tif]

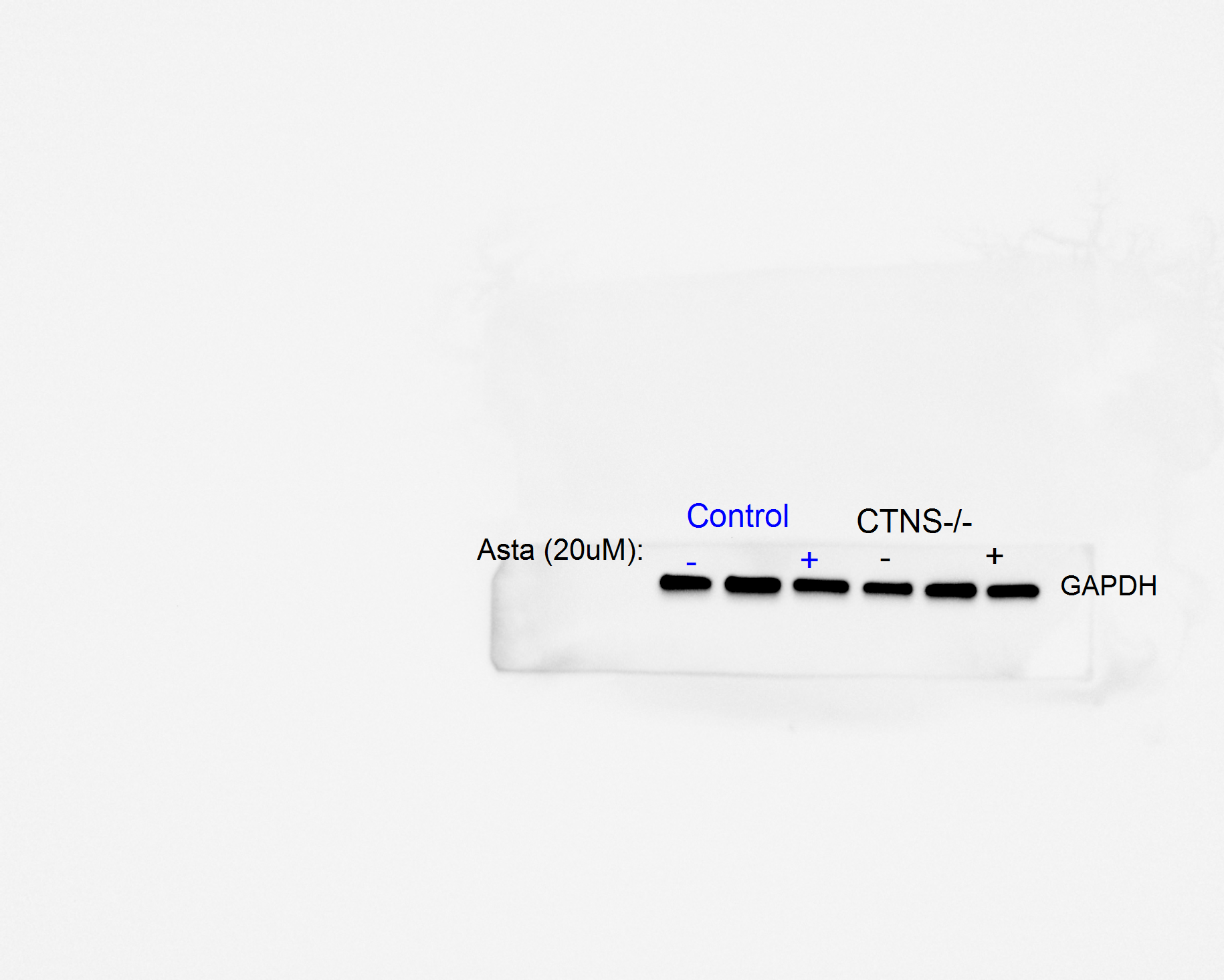

Supplement: Figure 8—source data 2. [file elife-94169-fig8-data2.zip › Figure 8-source data 2/Figure8C/LC3/8C - Gel 1_GAPDH.tif]

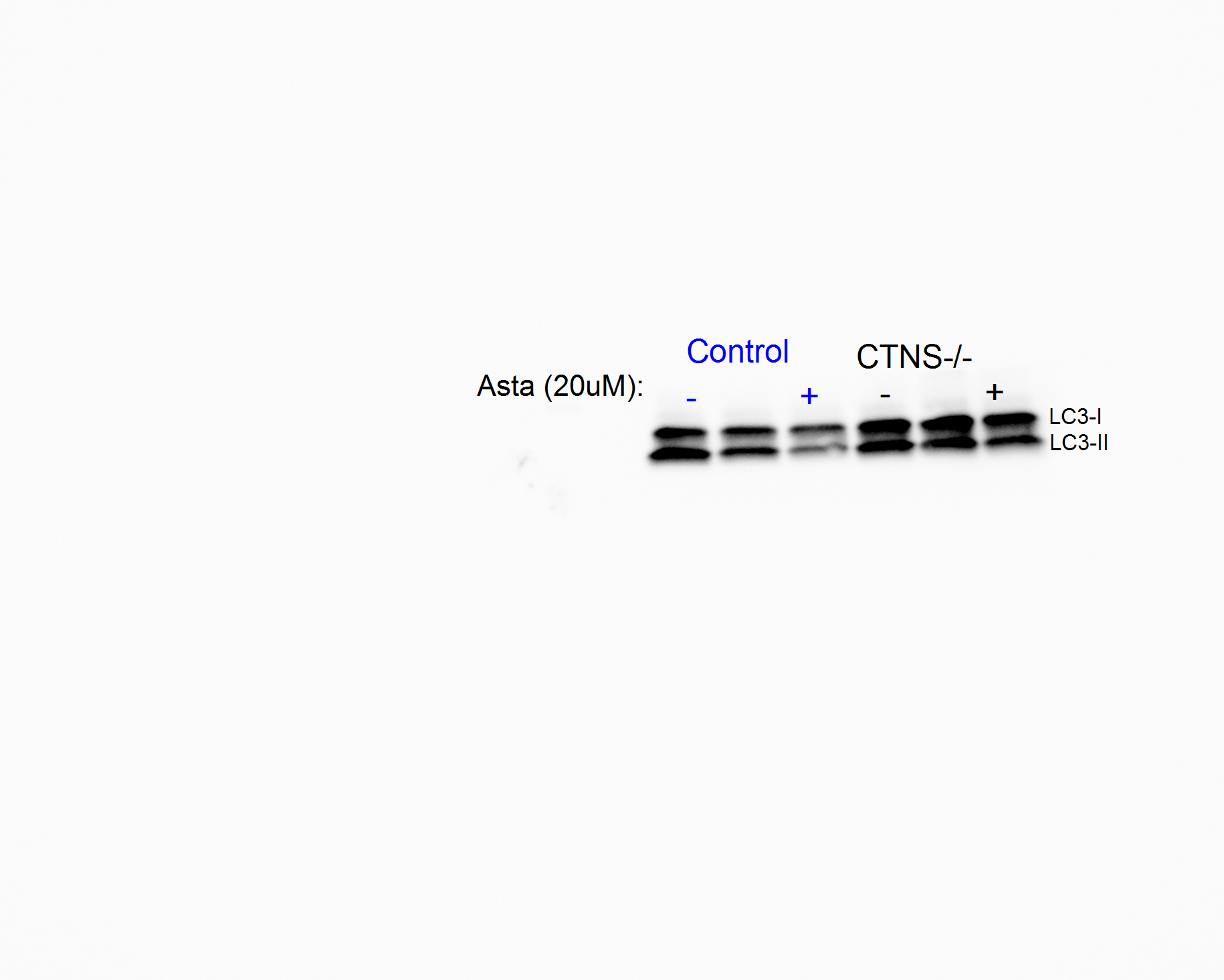

Supplement: Figure 8—source data 2. [file elife-94169-fig8-data2.zip › Figure 8-source data 2/Figure8C/LC3/8C - Gel 1_LC3.tif]

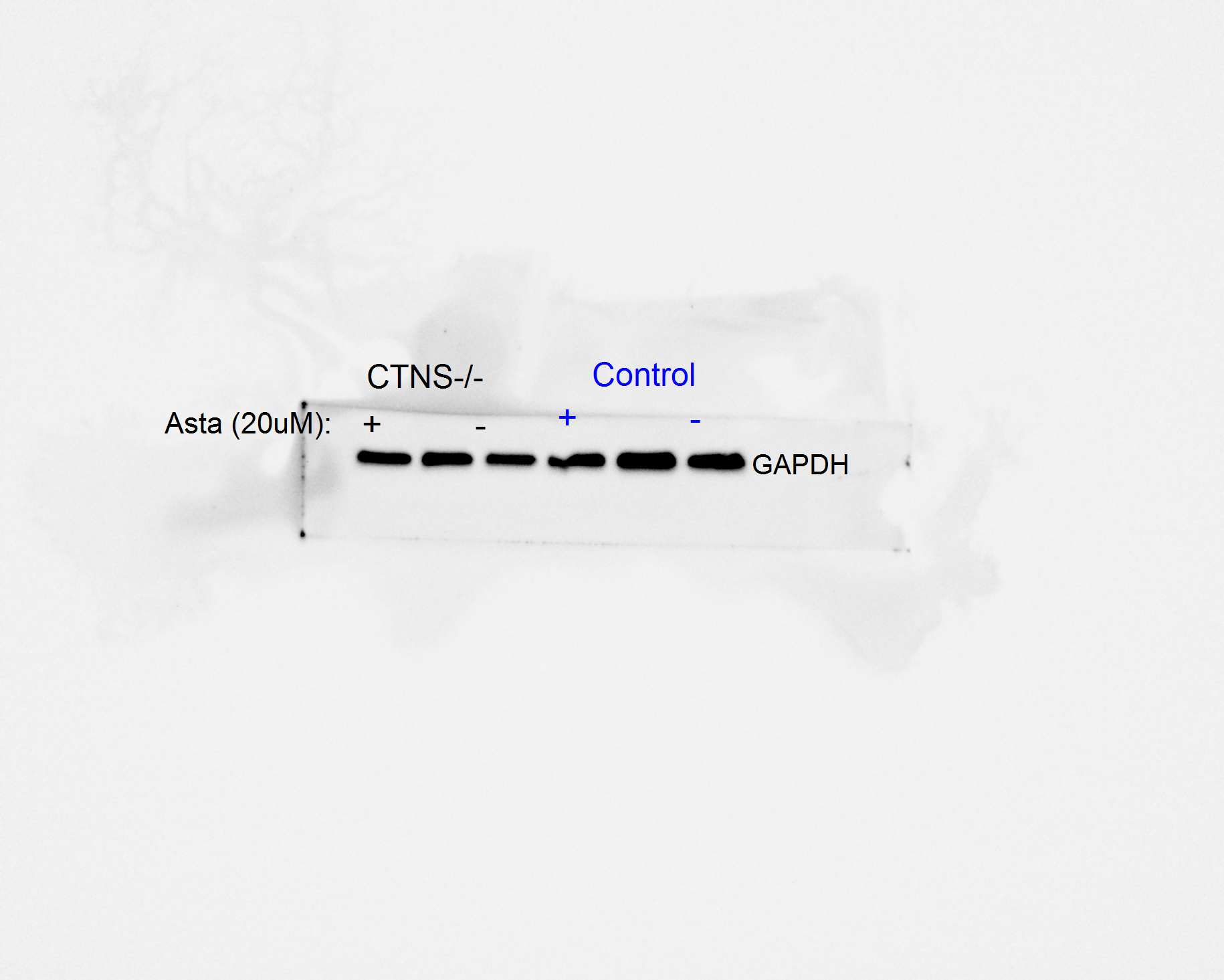

Supplement: Figure 8—source data 2. [file elife-94169-fig8-data2.zip › Figure 8-source data 2/Figure8C/LC3/8C - Gel 2_GAPDH.tif]

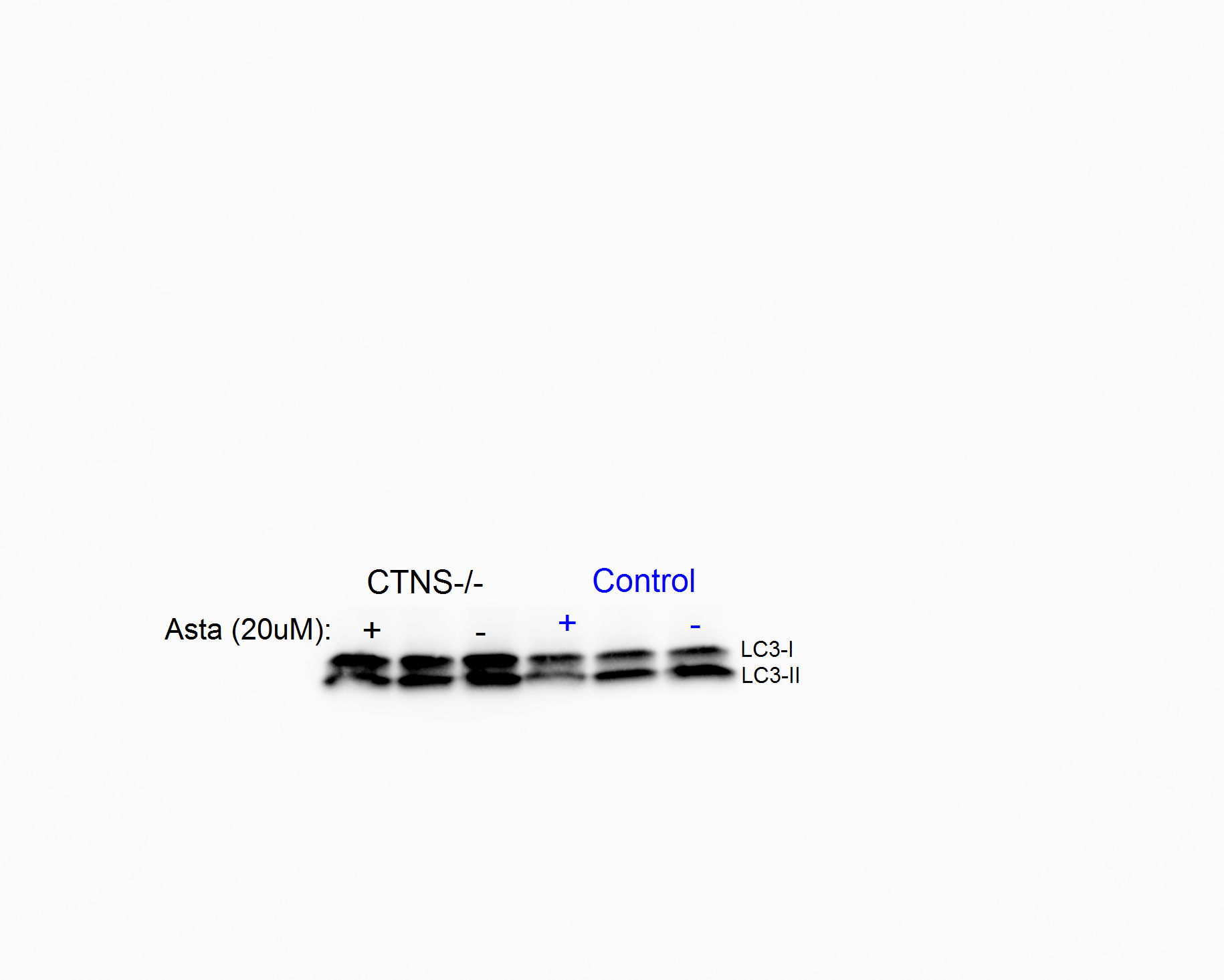

Supplement: Figure 8—source data 2. [file elife-94169-fig8-data2.zip › Figure 8-source data 2/Figure8C/LC3/8C - Gel 2_LC3.tif]

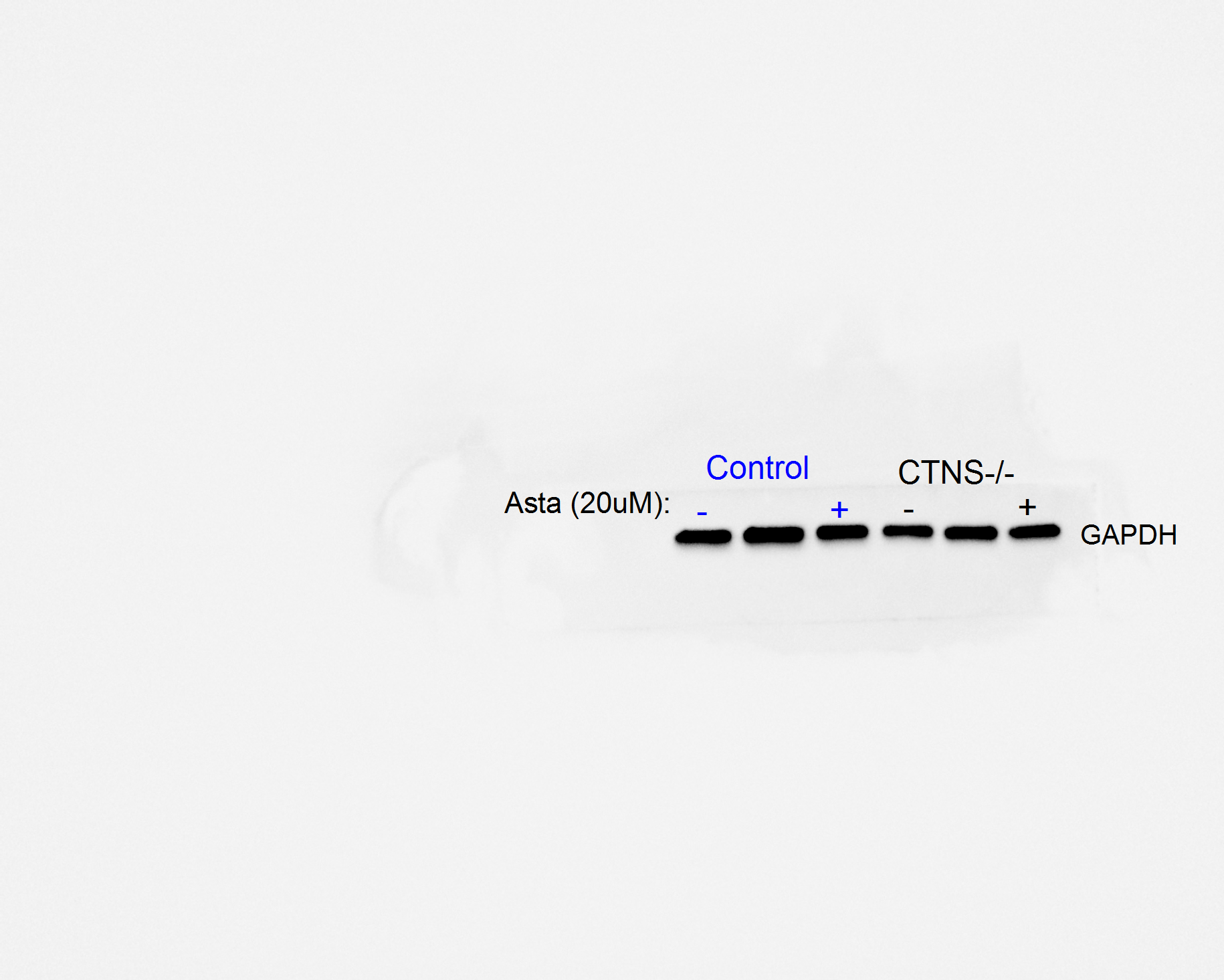

Supplement: Figure 8—source data 2. [file elife-94169-fig8-data2.zip › Figure 8-source data 2/Figure8C/LC3/8C - Gel 3_GAPDH.tif]

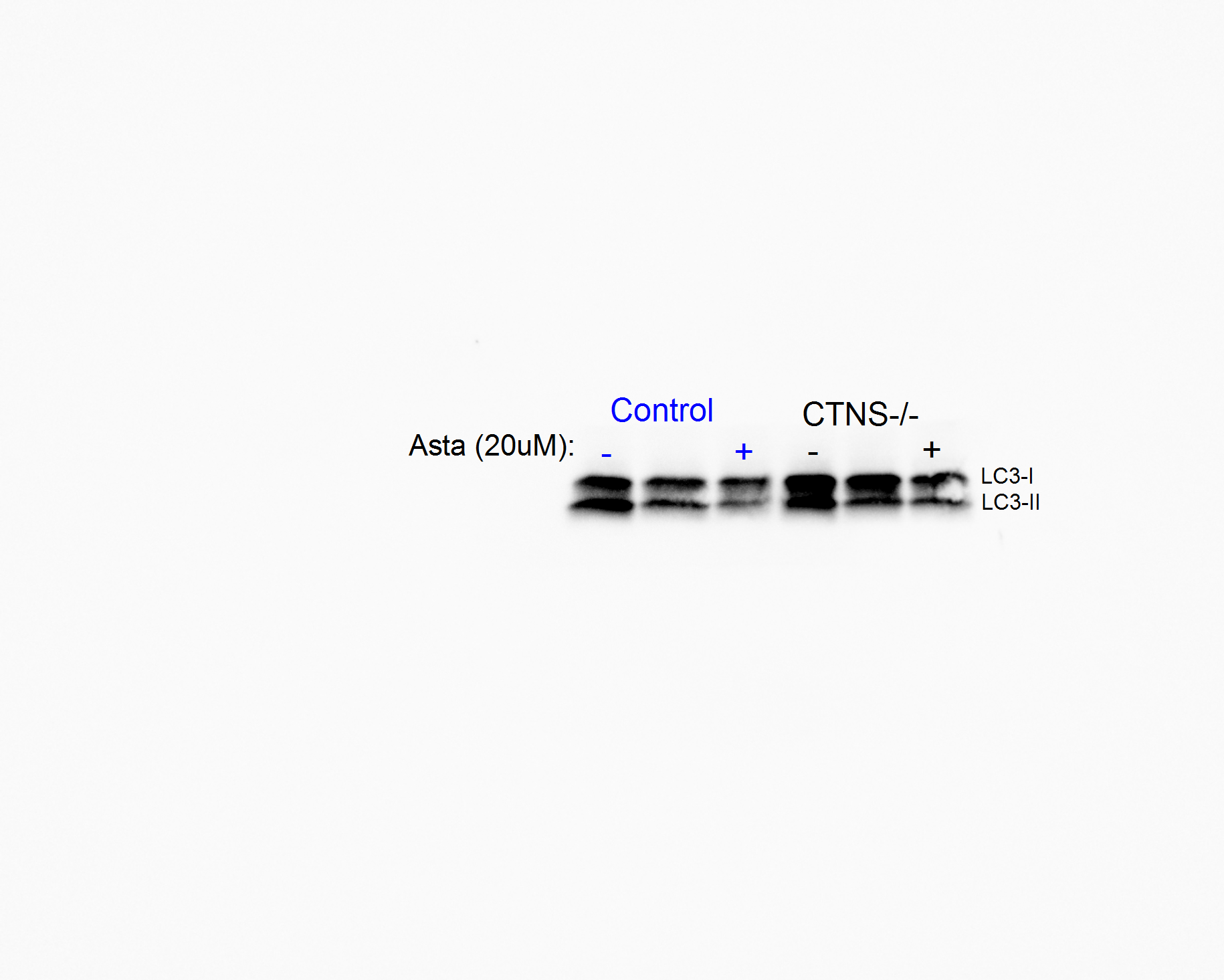

Supplement: Figure 8—source data 2. [file elife-94169-fig8-data2.zip › Figure 8-source data 2/Figure8C/LC3/8C - Gel 3_LC3.tif]

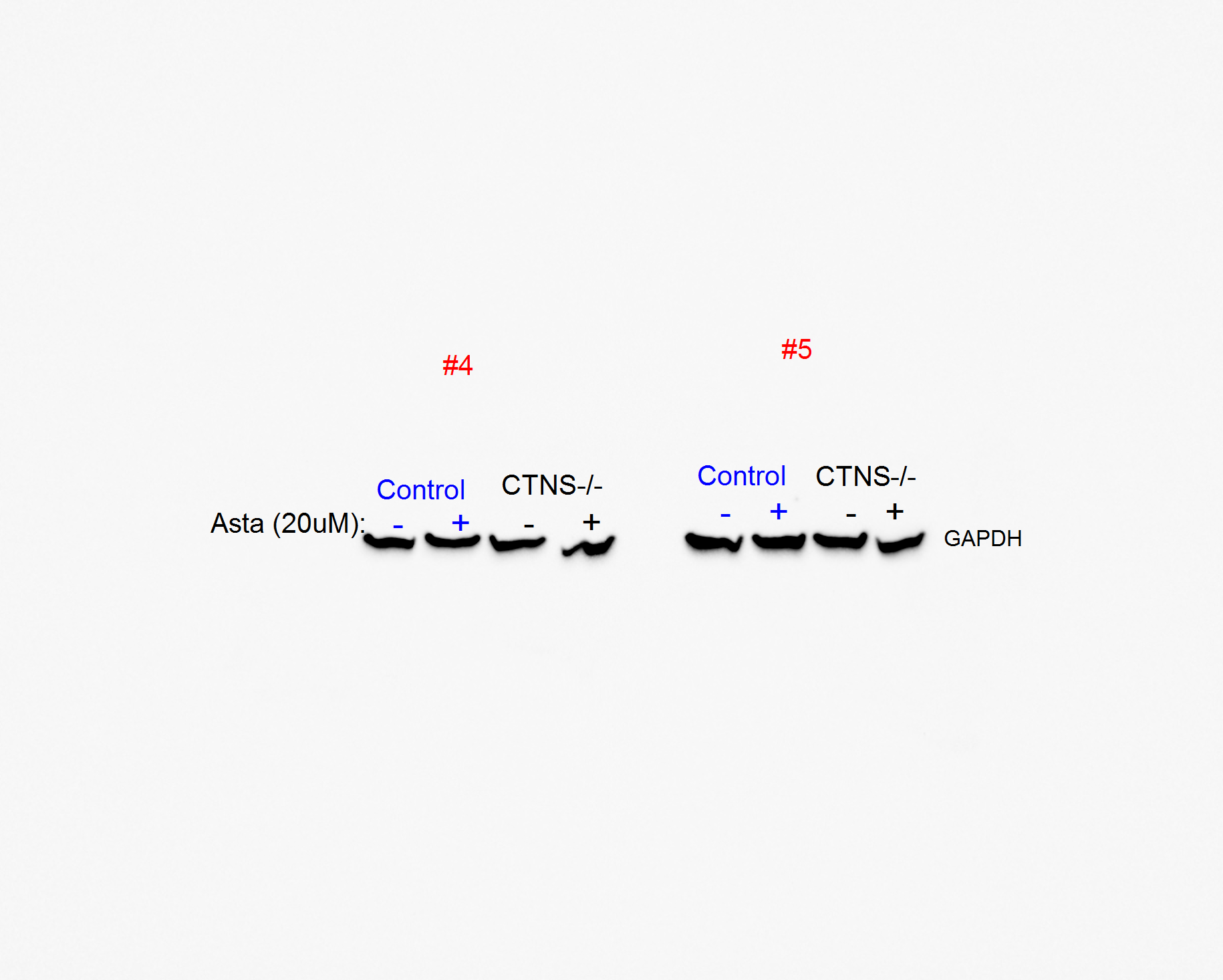

Supplement: Figure 8—source data 2. [file elife-94169-fig8-data2.zip › Figure 8-source data 2/Figure8C/LC3/8C - Gel 4&5_GAPDH.tif]

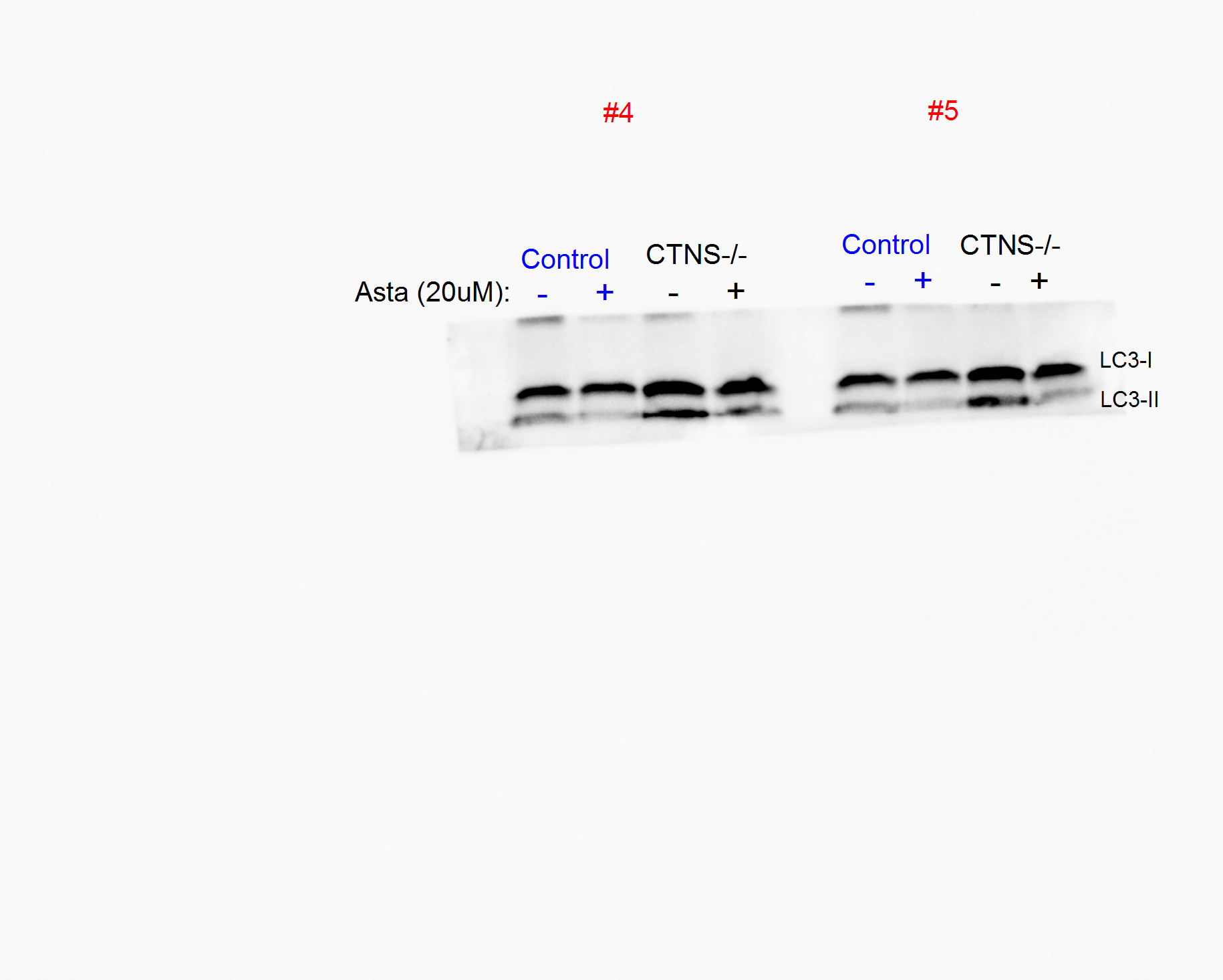

Supplement: Figure 8—source data 2. [file elife-94169-fig8-data2.zip › Figure 8-source data 2/Figure8C/LC3/8C - Gel 4&5_LC3.tif]
